# Photochemical Synthesis of Ynones from Aryl Aldehydes and Sulfone-Based Alkynes

**DOI:** 10.1021/acs.joc.5c01804

**Published:** 2025-09-08

**Authors:** Adam Cruise, Marcus Baumann

**Affiliations:** School of Chemistry, O’Brien Centre for Science, 8797 University College Dublin, Dublin 4, Belfield D04 N2E5, Ireland

## Abstract

Ynones are attractive molecular building blocks owing to their electrophilic character, which can be exploited in a variety of functionalization strategies, giving rise to valuable reaction products. This work presents a photochemical strategy for the direct generation of ynones from aldehydes and substituted alkynes bearing radicofugal groups, such as sulfones. Using TBADT (tetrabutylammonium decatungstate) as a photocatalyst, the direct photochemical synthesis of a variety of ynones is achieved in high yields and short reaction times. Exploiting a continuous flow reactor setup thereby provides for higher photon flux and scalability to generate gram quantities of the desired products. The beneficial effect of acetone as a cosolvent is reported, which minimizes double addition of the acyl radical generated from the aldehyde building blocks. Overall, this method represents an efficient and chemoselective route toward valuable ynones that may find further applications in industrial settings.

## Graphical abstract

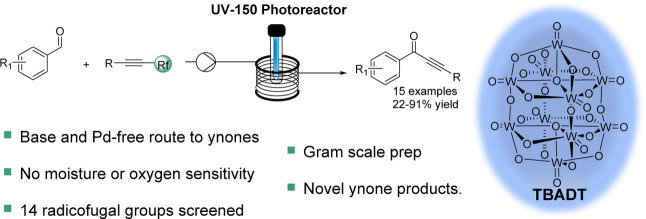

## Introduction

The field of photochemical C–H activation has seen tremendous progress over the past decade, which is largely driven by the development of a plethora of modern photocatalysts based on organic dyes and transition metal complexes.^1^ With numerous academic studies published, recent years have witnessed a shift in focus to impart more control and efficiency over these photochemical processes in view of sustainability and scalability, ultimately leading to applications in industrial settings.^2^ Of particular value has been the union of continuous flow processing and photochemistry, which overcomes limitations of batch photochemistry relating to mixing, heat transfer, and photon transfer.^3^ Moreover, flow chemistry offers spatiotemporal resolution, which in the context of photochemical reactions means that side reactions due to overirradiation of products can be avoided, thus increasing reaction selectivity and yield. While this has opened new avenues for the exploration and intensification of photochemical reactions toward their use in industry,^4^ flow photochemistry has also become a fertile area for the discovery of new chemical reactions.^5^

Many studies have reported the value of continuous flow approaches targeting the photochemical activation of C–H bonds in the presence of HAT (hydrogen atom transfer) catalysts.^6^ Prominent examples include the use of TBADT (tetrabutylammonium decatungstate) for the generation of radicals from light hydrocarbons (e.g., methane, ethane, propane),^7^ ethers (e.g., THF, dioxane, etc.),^8^ or aldehydes (including alkyl and aryl aldehydes).^9^ Further expansion has been seen by the introduction of radicofugal groups such as hypervalent iodine species, allowing for the alkynylation of alkyl radicals (Scheme 1A).^10^

**Scheme 1:**
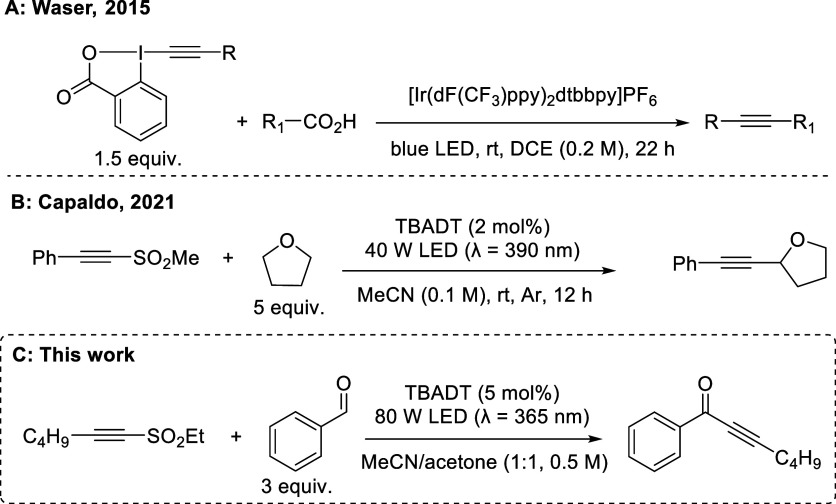
Overview of Photochemical Alkynylation Processes

A complementary yet underexplored approach uses radicofugal groups^11^ such as halides or sulfones that act as leaving groups on the π-acceptor (i.e., alkene or alkyne), ultimately enabling the formal coupling to an electron-rich π-system (Scheme 1B). A recent report by Capaldo and Ravelli demonstrates the use of TBADT (2 mol %) and alkynyl sulfones to forge new C–C bonds using ethers, acetals, and different hydrocarbons in the presence of UV-A light (390 nm).^12^ In a different study, Xu and Ma use blue light (450 nm) for an analogous process whereby alcohols are first activated via *N*-heterocyclic carbenes prior to photolysis with 4CzIPN as a photocatalyst (5 mol %) and coupling with alkynes bearing a methyl sulfonyl as a radicofugal group.^13^

Due to our interest in developing photochemical flow reactions for the scalable generation of valuable building blocks, we embarked on a study using aryl aldehydes as readily available radical precursors toward versatile ynone products^14^ (Scheme 1C). Specifically, the use of TBADT as a HAT catalyst in combination with different alkynyl sulfones was envisaged to generate the desired ynone products in the absence of Pd catalysts or strong inorganic bases, which would offer a more effective route toward these targets.

## Results and Discussion

Motivated by a lack of reported data on the effect of different sulfone moieties, our efforts commenced with a comparative study of various substituted alkyne sulfones and their reactivity toward the acyl radical generated in situ from benzaldehyde. The alkyne sulfones were prepared through deprotonation of the parent alkyne using ^n^BuLi followed by nucleophilic attack of a chosen disulfide (Scheme 2). The resulting thioether was purified by extraction, followed by its subsequent oxidation to the desired sulfone using hydrogen peroxide in methanol in the presence of catalytic amounts of ammonium molybdate. To evaluate whether extended conjugation impacts the performance of the radicofugal group, both *n*-hexyne and phenylacetylene were used in this study, affording the target compounds in very good yields of 60–80% (see Supporting Information for full details). In addition, a phosphonate ester (11a/b) and a trityl group (12a/b) were also explored as alternatives to the sulfone moiety.

**Scheme 2:**
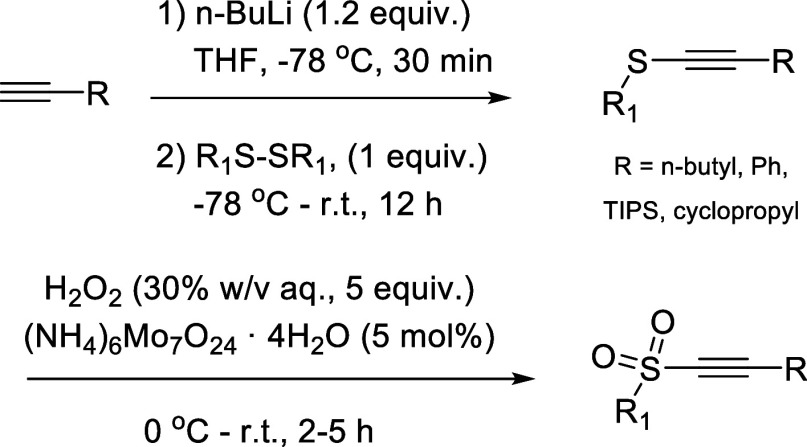
Synthesis of the Alkyne Sulfone Species Used in the Initial Performance Screen

To perform the initial test reactions, stock solutions containing benzaldehyde (3 equiv), the desired alkyne sulfone (1 equiv), and TBADT (5 mol %) were prepared in MeCN (0.5 M) and pumped through a Vapourtec E-Series photoreactor equipped with a LED emitting at 365 nm (90 W, *t*_Res_ = 30 min). The results for this screening are summarized in Figure 1.

**Figure 1:**
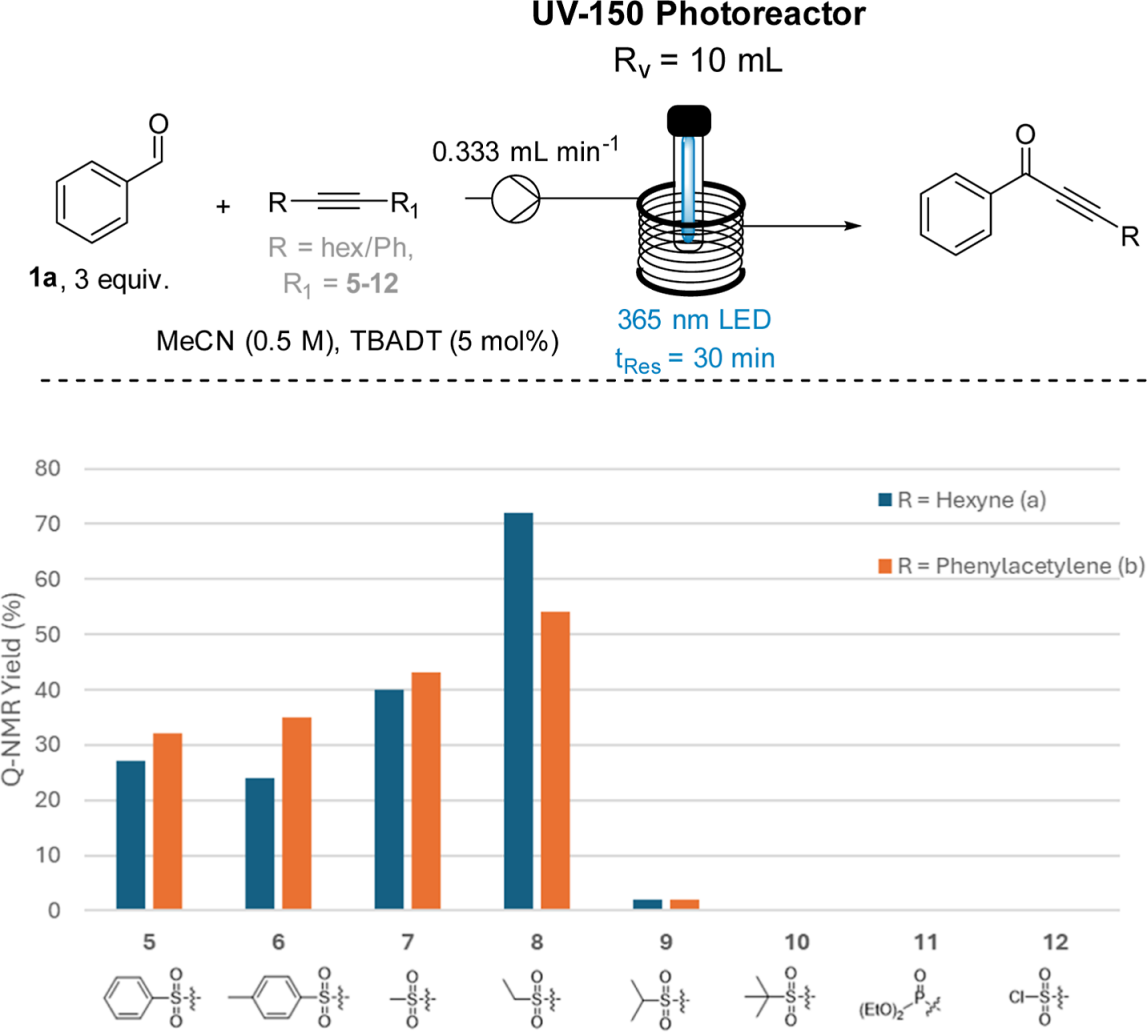
Performance of different sulfone-based radicofugal groups in the TBADT-catalyzed reaction of benzaldehyde with alkynes bearing sulfones and phosphonates.

To our surprise, sulfones bearing a phenyl (**5a**, **5b**) or *p*-tosyl (**6a**, **6b**) group performed worse than the corresponding methyl (**7a**, **7b**) and ethyl sulfones (**8a**, **8b**), despite past computational studies reporting that they would offer greater radical stability.^11^ This suggests that greater electronic stabilization likely makes for more stable radical leaving groups; however, steric hindrance and competing light absorbance must also be taken into consideration. This is supported by the observation that ethyl sulfones (**8a**, **8b**) show a better performance than the larger and more electron-rich isopropyl sulfones (**9a**, **9b**) that yielded only trace amounts of products for both *n*-hexyne and phenylacetylene. Even larger *t*-butyl sulfones (**10a**, **10b**) failed to generate any product. Moreover, alkynes bearing a phosphonate (**11a**, **11b**) also failed to give any target products, underpinning the importance of steric factors and the presence of competing chromophores. Chloro-substituted sulfones (**12**) were computationally shown to be favorable radical leaving groups in past studies;^11^ however, their preparation using the method described herein was not successful, leading to competitive decomposition. In conclusion and contrary to several previous studies employing methyl sulfones, alkyne sulfones bearing an ethyl group (**8a**, **8b**) emerged as the best radicofugal group examined, which appears to highlight the importance of balancing electronic stabilization and steric hindrance. Further computational studies would be needed to validate these principal findings but are beyond the scope of this work.

Having identified alkynes bearing ethyl sulfones as the best radicofugal group, a thorough optimization was conducted using benzaldehyde (**1a**) as the model substrate (see Supporting Information for full details). One noteworthy observation was that secondary adducts were obtained under unoptimized conditions, showing the competitive addition of a second benzaldehyde molecule to the newly formed ynone via a Giese-type process. It was found that the undesired side reaction can be suppressed by addition of acetone to the solvent system. Acetone acting as a potential triplet sensitizer can alter photochemical reaction paths as previously observed by us^15^ and others,^16^ marking a simple solution to the observed side reaction. Therefore, a solvent system comprised of MeCN and acetone (1:1 by volume) at an alkyne concentration of 0.5 M was employed, as it gave the highest yield of the desired ynone product **3a** (isolated yield of 77%, 1 mmol scale, Scheme 3) without any side product formation observed. Although this addition of acetone did decrease the rate of the reaction (72% in 30 min with 100% MeCN, 77% in 40 min with 1:1 MeCN/acetone), this adjustment was favored going forward as it consistently led to cleaner reaction mixtures.

**Scheme 3:**
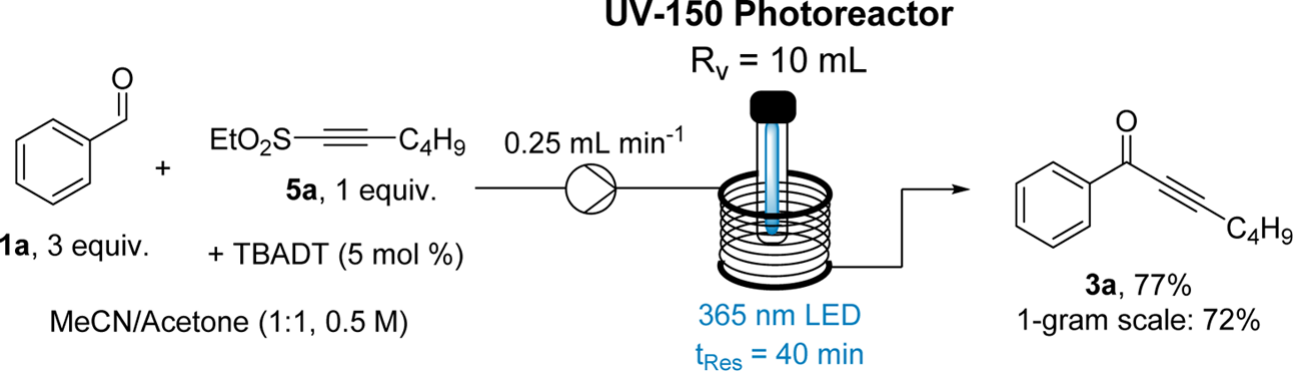
Optimized Conditions for Formation of Ynone **3a** and Reaction Scale-Up

Employing these optimized conditions, a gram-scale reaction was conducted using 7.5 mmol of the limiting alkyne-sulfone reagent (**5a**), which produced 1 g of the desired ynone (**3a**) with a yield of 72%, affording a space-time yield of 0.54 mmol L^–1^ h^–1^. These promising results demonstrate the facile scalability of this methodology as 1.0 g of ynone product could be generated using a 10 mL reactor coil in a total processing time of 1 h.

Having demonstrated the scalability of the method, its substrate scope was explored next, initially varying the aldehyde component in the presence of the hexyne-derived ethyl sulfone **5a** (Scheme 4). Both electron-deficient and electron-rich aldehydes worked well in this process; however, better yields were typically observed when using electron-rich aromatic systems. This can be seen when comparing the outcome for 4-methoxybenzaldehyde, yielding 91% of the target ynone **3b**, while the electron-deficient 4-trifluoromethyl-substituted benzaldehyde **1c** gave a lower yield of 59%. Halides (e.g., Cl and Br) were tolerated despite the use of UV-A light, and the ynone derived from 4-bromobenzaldehyde was obtained in a yield of 59%, demonstrating good chemoselectivity, which would be very challenging in a traditional acyl Sonogashira reaction. Similarly, boronic esters such as **1f** afforded the desired product in high yield, further highlighting the orthogonal nature of this method to the traditional acyl Sonogashira reaction. Heteroaromatic systems (**1g** and **1i**) can be employed, yet further optimization is required to increase the yields of the respective products. Reactor fouling was observed for (**1k**) due to precipitation of reaction intermediates and subsequent reactor clogging. Alkyl-bearing substrates such as cyclohexanecarboxaldehyde (**1j**) were also tolerated under the standard conditions. Unfortunately, 2-methylbenzaldehyde failed to give the target ynone **3n,** likely due to steric hindrance as well as the preferable photoenolization under the reaction conditions. 4-Cyanobenzaldehyde as well as aldehydes containing styryl and naphthyl systems demonstrated reduced conversion of the limiting sulfonated alkyne while returning a complex reaction mixture, indicating their propensity for competing side reactions instead of affording products **3l**, **3m**, and **3o**.

**Scheme 4:**
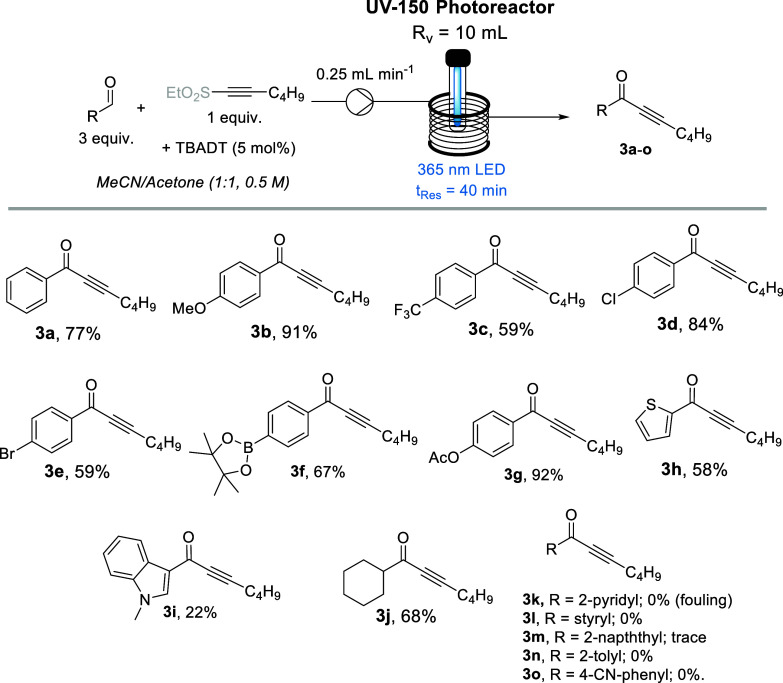
Substrate Scope of Aldehydes and Ethyl Sulfone-Substituted Hexyne

Following the variation of the aldehyde, a small selection of alternative alkynes was explored, as summarized in Scheme 5. This demonstrated that the use of phenylacetylene substituted with an ethyl sulfone gave good results for both electron-rich and electron-poor aryl aldehydes (**4a**–**c**). Use of a cyclopropane moiety on the alkyne is tolerated with no evidence for decomposition of the cyclopropane ring, affording product **4d** in good yield. Use of heptanal also allowed for a comparison of our method with prior work.^11^ Using our standard conditions, a slightly lower yield of 50% was observed in comparison with the previously reported 67% yield. However, the increased concentration and shortened reaction time achieved by the implementation of continuous flow technology allowed for a space-time yield of 0.38 mol L^–1^ h^–1^, which represents a significant increase by a factor of 2700 when compared to the previous batch study. Unfortunately, the use of a TIPS-protected alkyne showed no reactivity toward the formation of target ynone **4f**, which may highlight that this acceptor is sterically too crowded as the acyl radical approaches the alkyne moiety.

**Scheme 5:**
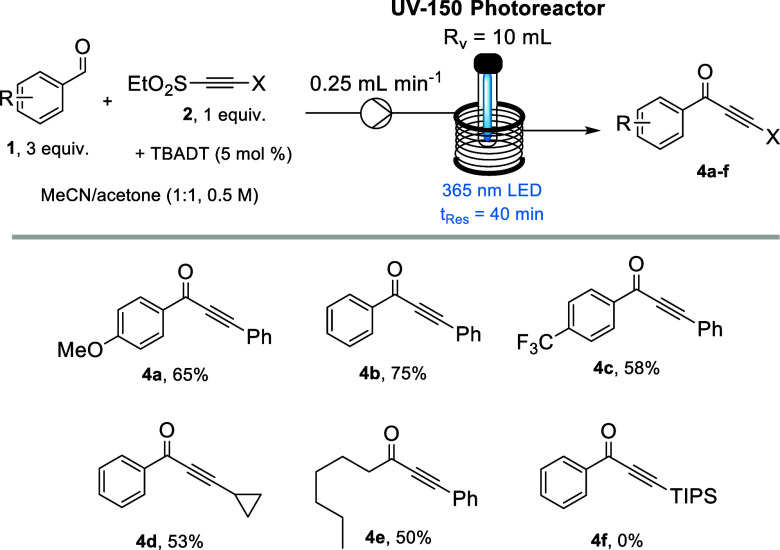
Substrate Scope for Aldehydes Reacting with Different Alkyne Sulfones

## Conclusion

In summary, we report the development of a photochemical process allowing the use of aldehydes and sulfone-bearing alkynes for the construction of C_sp_2__–C_sp_ bonds using TBADT as a readily available HAT catalyst. By screening different radicofugal leaving groups, our study revealed that ethyl sulfones provide the best reactivity for this transformation, which tolerates a variety of functional handles that would be challenging using alternative conditions (i.e., aryl bromides, boronic esters, cyclopropanes, etc.). Moreover, we have found that acetone as a cosolvent suppresses the double addition of the acyl radical species to the desired alkyne product. The developed continuous flow process allows us to prepare these versatile ynone species on a gram scale, thus demonstrating the potential of this process for industrial exploitation.

## Supplementary Material



## Data Availability

The data underlying this study are available in the published article and its Supporting Information.

## Notes

Optimization tables; characterization data; experimental and spectroscopic data; copies of ^1^H, ^13^C, and ^19^F NMR spectra; and pictures of the flow equipment (PDF)

## References

[1] Nagar B., Dhar B. (2022). Photochemical C-H Arylation of Naphthoquinones
Using Eosin Y. ACS Omega.

[2] Williams J. D., Kappe C. O. (2020). Recent advances toward sustainable
flow photochemistry. Curr. Opin. Green Sustainable
Chem..

[3] Buglioni L., Raymenants F., Slattery A., Zondag S. D. A., Noël T. (2022). Technological Innovations in Photochemistry for Organic
Synthesis: Flow Chemistry, High-Throughput Experimentation, Scale-up,
and Photoelectrochemistry. Chem. Rev..

[4] Moschetta E. G., Cook G. C., Edwards L. J., Ischay M. A., Lei Z., Buono F., Lévesque F., Garber J. A. O., MacTaggart M., Sezen-Edmonds M., Cole K. P., Beaver M. G., Doerfler J., Opalka S. M., Liang W., Morse P. D., Miyake N. (2024). Photochemistry
in Pharmaceutical Development: A Survey of Strategies and Approaches
to Industry-wide Implementation. Org. Process
Res. Dev..

[5] Alfano A. I., García-Lacuna J., Griffiths O. M., Ley S. V., Baumann M. (2024). Continuous
flow synthesis enabling reaction discovery. Chem. Sci..

[6] Capaldo L., Ravelli D., Fagnoni M. (2022). Direct Photocatalyzed
Hydrogen Atom Transfer (HAT) for Aliphatic C–H Bonds Elaboration. Chem. Rev..

[7] Laudadio G., Deng Y., van der
Wal K., Ravelli D., Nuño M., Fagnoni M., Guthrie D., Sun Y., Noël T. (2020). C­(sp^3^)–H functionalizations of light
hydrocarbons using decatungstate
photocatalysis in flow. Science.

[8] Wan T., Capaldo L., Laudadio G., Nyuchev A. V., Rincon J. A., García-Losada P., Mateos C., Frederick M. O., Nuño M., Noël T. (2021). Decatungstate-Mediated
C­(sp^3^)–H Heteroarylation via Radical-Polar Crossover
in Batch and Flow. Angew. Chem., Int. Ed..

[9] Cruise A., Baumann M. (2023). TBADT-Mediated, C–C
Bond Formation
Exploiting Aryl Aldehydes in a Photochemical Flow Reactor. ChemCatChem.

[10] Le
Vaillant F., Courant T., Waser J. (2015). Room-Temperature Decarboxylative
Alkynylation of Carboxylic Acids Using Photoredox Catalysis and EBX
Reagents. Angew. Chem., Int. Ed..

[11] Wang P. F., Feng Y., Cheng Z., Wu Q., Wang G., Liu L., Dai J., Xu J., Xu H. (2015). Transition-Metal-Free Synthesis of Ynones via Decarboxylative Alkynylation
of α-Keto Acids under Mild Conditions. J. Org. Chem..

[12] Capaldo L., Ravelli D. (2021). Decatungstate as Direct
Hydrogen Atom Transfer Photocatalyst
for SOMOphilic Alkynylation. Org. Lett..

[13] Xu P., Ma C. (2024). Scalable deoxygenative
alkynylation of alcohols via flow photochemistry. Commun. Chem..

[14] Whittaker R. E., Dermenci A., Dong G. (2016). Synthesis of Ynones
and Recent Application in Transition-Metal-Catalyzed Reactions. Synthesis.

[15] O’Hanlon D., Davin S., Glennon B., Baumann M. (2025). Metal-free [2 + 2]-photocycloaddition of unactivated
alkenes enabled by continuous flow processing. Chem. Commun..

[16] Ho C., Chung W. (1997). Photochemistry of acetone
in the presence of exocyclic olefins: an unexpected competition between
the photo-Conia and Paternó-Büchi reactions. Chem. Commun..

