# Supplementary material for: Photochemical Synthesis of Ynones from Aryl Aldehydes and Sulfone-Based Alkynes
Source: J Org Chem. 2025 Sep 8;90(37):13175–80. doi: 10.1021/acs.joc.5c01804 (PMC12455645; doi:10.1021/acs.joc.5c01804)
Supplement: Supplementary file 1 [file jo5c01804_si_001.pdf]

Supporting Information

Photochemical Synthesis of Ynones from Aryl Aldehydes and Sulfone-Based Alkynes

Adam Cruise,[a] Marcus Baumann*,[a]

[a] School of Chemistry, University College Dublin, O'Brien Centre for Science, Belfield, Dublin 4, Ireland

*Email: marcus.baumann@ucd.ie

Table of Contents:

1. Materials and Methods S2

2. Experimental Procedures S3
   - 2.1. Synthesis of Alkyne Sulfones S3
   - 2.2. Synthesis of Alkyne Phosphonates S4
   - 2.3. Synthesis of TBADT S4
   - 2.4. General Procedure for Alkyne Sulfone Synthesis S5
   - 2.5 Procedure for Gram Scale Reaction for Formation of Ynone **3a** S6

3. Reaction Optimisations S7
   - 2.5. Initial Parameter Screening S7
   - 2.6. Suppression of Second Addition – Solvent Study S9
   - 2.7. Re-Evaluation of Optimization with New Solvent System S9

4. Characterization of Ynone Products S11
5. Characterization of Alkyne Adducts S19
6. Copies of NMR Spectra S25

1. Materials and Methods

Unless otherwise stated, all solvents were purchased from Fisher Scientific and used without further purification. Also, unless otherwise stated, all substrates and reagents were purchased from Fluorochem or Sigma-Aldrich and used as received.

^{1}H NMR spectra were recorded on 400 and 500 MHz instruments and are reported relative to the residual solvent: $CDCl_3$ (δ 7.26 ppm) or DMSO-d_6 (δ 2.50 ppm). $^{13}C\{^{1}H\}$ NMR spectra were recorded on the same instruments (100 and 125 MHz) and are reported relative to $CHCl_3$ (δ 77.16 ppm) or DMSO-d_6 (δ 39.52 ppm). ^{19}F NMR were recorded at 376 MHz. Data for ^{1}H NMR are reported as follows: chemical shift (δ/ ppm) (integration, multiplicity, coupling constant (Hz)). Multiplicities are reported as follows: s = singlet, d = doublet, t = triplet, q = quartet, p = pentet, m = multiplet, br s = broad singlet, app = apparent. Data for $^{13}C\{^{1}H\}$ NMR are reported in terms of chemical shift (δ/ppm) and multiplicity (C, CH, CH_2, or CH_3). COSY and HSQC experiments were used in the structural assignment.

IR spectra were obtained by use of a Bruker Platinum spectrometer (neat, ATR sampling) with the intensities of the characteristic signals being reported as weak (w, <20% of the tallest signal), medium (m, 21–70% of the tallest signal), or strong (s, >71% of the tallest signal).

High-resolution mass spectrometry was performed using the indicated techniques on a micromass LCT orthogonal time-of-flight mass spectrometer with leucine-enkephalin (Tyr-Gly-Phe-Leu) as an internal lock mass. GC–MS was performed on a Waters GCT Premier Agilent 7898 system (column Macherey–Nagel; Optima 5 MS, length 15 m, diameter 0.25 mm).

Continuous flow experiments were performed on a Vapourtec E-series system equipped with peristaltic pumps and a dynamic BPR achieved through utilisation of a peristaltic pump in a reverse direction (1-9 bar, Vapourtec). For photochemical experiments the UV-150 module was used in combination with a high-power LED (365 nm) regulated between 70-100 W and cooled to 25-40 °C by passing a stream of compressed air through the reactor unit. Reactor coils were made of PFA tubing (i.d. 1/16 inch) with a volume of 10 mL.

UV−vis measurements were performed with a Shimadzu UV-1800 UV spectrophotometer.

TLC was performed on Merck pre-coated Silica gel 60 F254 aluminium plates with realisation by UV irradiation at 254 nm, $KMnO_4$ and vanillin stain. Flash chromatography was performed using Macherey-Nagel silica gel 60 M, with a particle range of 0.04 - 0.063 mm.

2. Experimental Procedures

2.1. General procedure for the Synthesis of Alkyne Sulfones

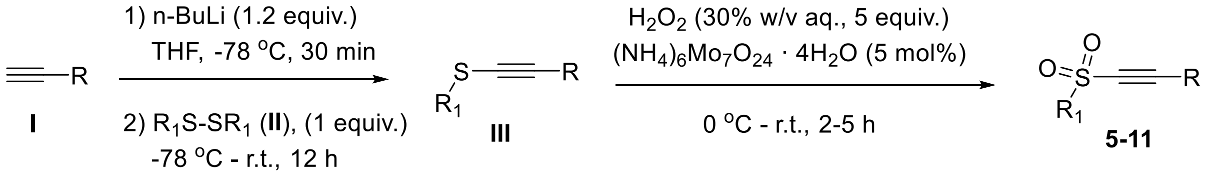

The chosen alkyne **I** (5.2 mmol) in THF (anhydrous, 25 mL) was stirred and cooled to -78 °C under a nitrogen atmosphere. nBuLi (2.1 mL, 2.5 M in hexanes, 5.2 mmol) was added dropwise via syringe to the stirred solution of alkyne followed by stirring for a further 30 minutes. Next, the corresponding disulfide **II** (5 mmol) was added dropwise at -78 °C. The solution was stirred for a further 15 minutes at -78 °C before allowing the reaction mixture to warm to room temperature overnight. A precipitate was typically observed the following morning, if not the reaction was warmed gently in a water bath at 35 °C for a further 4 hours. Once a significant precipitate had formed the reaction mixture was exposed to open atmosphere before a saturated aqueous solution of K_2CO_3 (10 mL) was slowly added. The mixture was washed twice with Et_2O (2 x 15 mL) and concentrated under reduced pressure to afford a crude mixture containing the concentrated alkyne-thioether intermediate (**III**). The retained aqueous layer was treated with domestic grade bleach to oxidise any residual thiols generated from the prior step.

The resulting crude solution containing intermediate **III** was used in the next step without purification to avoid prolonged exposure of thiol intermediates. A solution of the intermediate thioether-alkyne intermediate (**III**) in MeOH (12.5 mL) was prepared and stirred at room temperature. Ammonium molybdate (0.25 mmol, 0.309 g) was added and the reaction mixture was cooled to 0 °C using an ice-bath, before adding H_2O_2 dropwise (10 mmol, 30% w/v, in H_2O, 1.3 mL). Next, the reaction mixture was removed from the ice-bath and stirred at room temperature, while monitoring the reaction progress by TLC (c-Hex/EtOAc, 4:1) with typical completion time within 2h. A second portion of H_2O_2 can be added without the risk of over-reaction of the target product if the reaction has not reached 100% consumption of the starting thioether after 2 hours (5 mmol, 30% w/v, in H_2O, 0.65 mL). The crude solution was then quenched with saturated $NaHCO_3$ solution (50 mL) and the target sulfone-alkyne was extracted into Et_2O (2 x 30 mL) and the combined organic layers washed sequentially with water and brine. $MgSO_4$ was added to the collected organic layers, and the crude mixture was filtered and dried in *vacuo*. The concentrated crude material was then separated by flash column chromatography using an isocratic eluent system (c-Hex/EtOAc, 4:1) to afford the target alkyne-sulfone product (**5-11**) in a 60-80% yield. It should be noted that in certain cases the target sulfonated alkyne was of an acceptable purity without further purification, typically these included the ethyl and methyl substituted sulfones.

2.2. Synthesis of Alkyne Phosphonates

The chosen alkyne **I** (5.2 mmol) in THF (anhydrous, 15 mL) was stirred and cooled to -78 °C under a nitrogen atmosphere. n-BuLi (2.1 mL, 2.5 M, 5.2 mmol) in hexanes was added dropwise via syringe to the stirred solution of alkyne and let stir for a further 30 minutes. Following the addition of n-BuLi in hexanes the diethyl chlorophosphate (5 mmol, 862 mg) in THF (anhydrous, 10 mL) was added dropwise to the stirring reaction mixture at -78 °C. The solution was stirred for a further 15 minutes at -78 °C before allowing the reaction mixture to warm to room temperature, upon which time a white precipitate could be seen forming. A saturated solution of K_2CO_3 in water (10 mL) was slowly added to the stirred solution. The mixture was washed twice successfully with EtOAc (2 x 15 mL) and concentrated under reduced pressure to afford a crude solution containing a concentrated alkyne-phosphonate which was utilised for screening purposes without further purification.

2.3. Synthesis of TBADT

Tetrabutylammonium bromide (2.4 g, 7.4 mmol) and sodium tungstate dihydrate (5.0 g, 15.0 mmol) were dissolved each in 150 mL of deionised water in two Erlenmeyer flasks and kept at 90 °C under vigorous stirring. HCl (aq., 12 M) was added dropwise to both solutions to adjust the pH to 2. The two solutions were then combined and maintained at 90 °C for 30 min under stirring. A white suspension n of TBADT formed and, after cooling to room temperature, was filtered via a glass sintered funnel. The white powder was washed with water (3 × 10 mL) and then dried in a vacuum oven (40 °C, 100 mbar) overnight. The resulting white solid was suspended in dichloromethane (20 mL per gram of solid) and kept under stirring for two hours. The pure TBADT was separated from the yellow solution by filtration on a glass sintered funnel and dried in a vacuum oven (40 °C, 100mbar) overnight to yield the target TBADT (68%, 3.4. The resultant TBADT was characterised by UV-Vis spectroscopy as described previously, yielding 68% of the target TBADT (3.40 g, 1 mmol).

The quality of TBADT synthesised was measured by UV spectroscopy which can be seen below along with the structure of the TBADT anion (**Fig S1**).

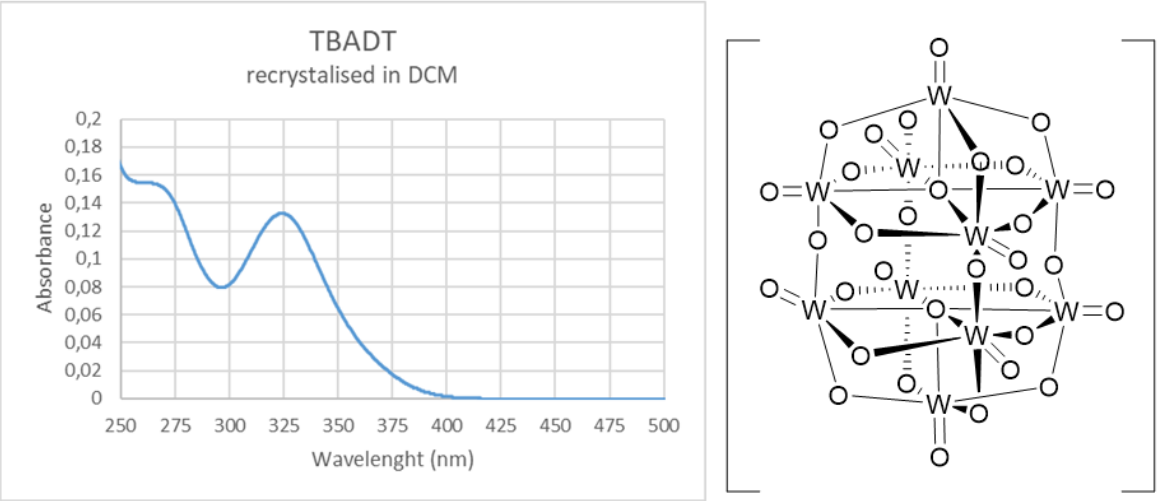

Figure S1: Absorbance and structure of TBADT.

2.4. General Procedure of Ynone Synthesis

Tetrabutylammonium decatungstate (84 mg, 5 mol%), corresponding aldehyde **1** and sulfonated alkyne **5-11** were charged in a sample vial and dissolved into a solution of MeCN: Acetone (1:1, 0.5 M). The solution was stirred until homogenous. The resultant reactant solution was pumped using an Vapourtec V-3 peristatic pump into a pre-charged UV-150 Vapourtec photochemical reactor equipped with a 10 mL PTFE coiled tube with an internal diameter of 1/32", cooled by compressed air. A backpressure of 1 bar was applied through the flow command software modulating a second V-3 pump in reverse to dynamically maintain a 1 bar pressure on the system. The UV-150 photochemical reactor was conditioned by passing 15 mL total volume of solvent (MeCN: Acetone, 1:1) under the same operating conditions as the reaction being conducted. The prepared reaction mixture was subsequently pumped at a flow rate of 0.2 mL min^{-1} achieving a residence time of 40 minutes within the 10 mL photoreactor coil. An overview of this process and accompanying picture can be seen below (**Scheme S1**). The resultant crude solution was collected in a round bottomed flask, condensed *en vacuo* and analysed by ^{1}H-NMR. TLC analysis using c-hex as an eluent system was employed to assess the ideal purification system for the target compound. It is noted that the apolar nature of the products and starting aldehydes made purification challenging, often giving R_f values > 0.6 and as high as 0.9, the proximity of the aldehyde and product For standard phase purification, flash chromatography was conducted using c-hex, and in the case of reverse phase chromatography a gradient elution was employed (H_2O/MeCN, 9:1 – 1:9) over a duration of 40 minutes using a PuriFlash X520PLUS prep chromatography system and an Interchim PF-15C18XS-F0012 column. It should be noted that the resultant ynones could be further functionalised without chromatography purification but rather by using extraction employing Et_2O and water to wash the crude reaction mixture.

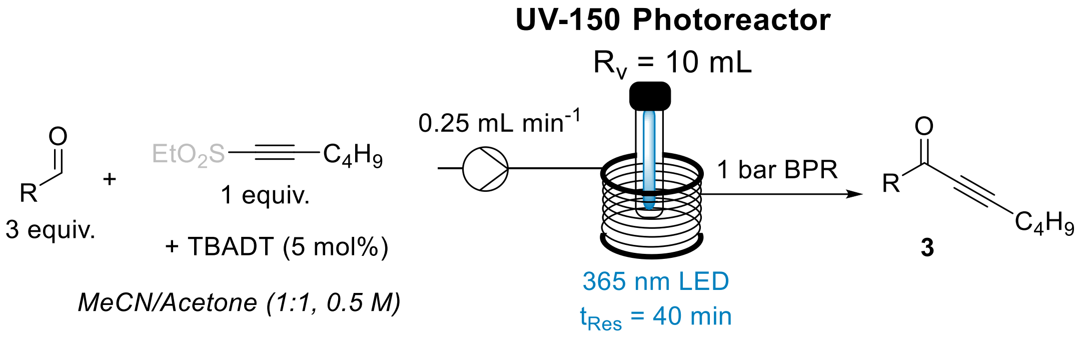

Scheme S1: Flow schematic for the formation of ynones.

2.5 Procedure for Gram Scale Reaction for the Formation of Ynone 3a

Tetrabutylammonium decatungstate (84 mg, 5 mol%), benzaldehyde (2.38 g, 22.5 mmol) and sulfonated alkyne **5a** (1.3 g, 7.5 mmol) were charged in a sample vial and dissolved in MeCN: Acetone (1:1, 0.5 M). The resulting solution was stirred with a magnetic stir bar until homogenous. The reaction procedure then followed the same steps as outlined in the general procedure for ynone synthesis **(Section 2.4),** being a flow rate of 0.25 mL/min in a 10 mL PFA reactor coil. Processing the 15 mL of reaction mixture required a total operational time of 1 hour. In this instance, isolation of the pure product (1.0 g, 5.4 mmol, 72%) was possible using flash chromatography using an isocratic elution profile (cHex-EtOAc, 99:1) with the target ynone having an R_f value of 0.75. A labelled photograph of the photochemical flow reactor can be seen below (**Fig S2**).

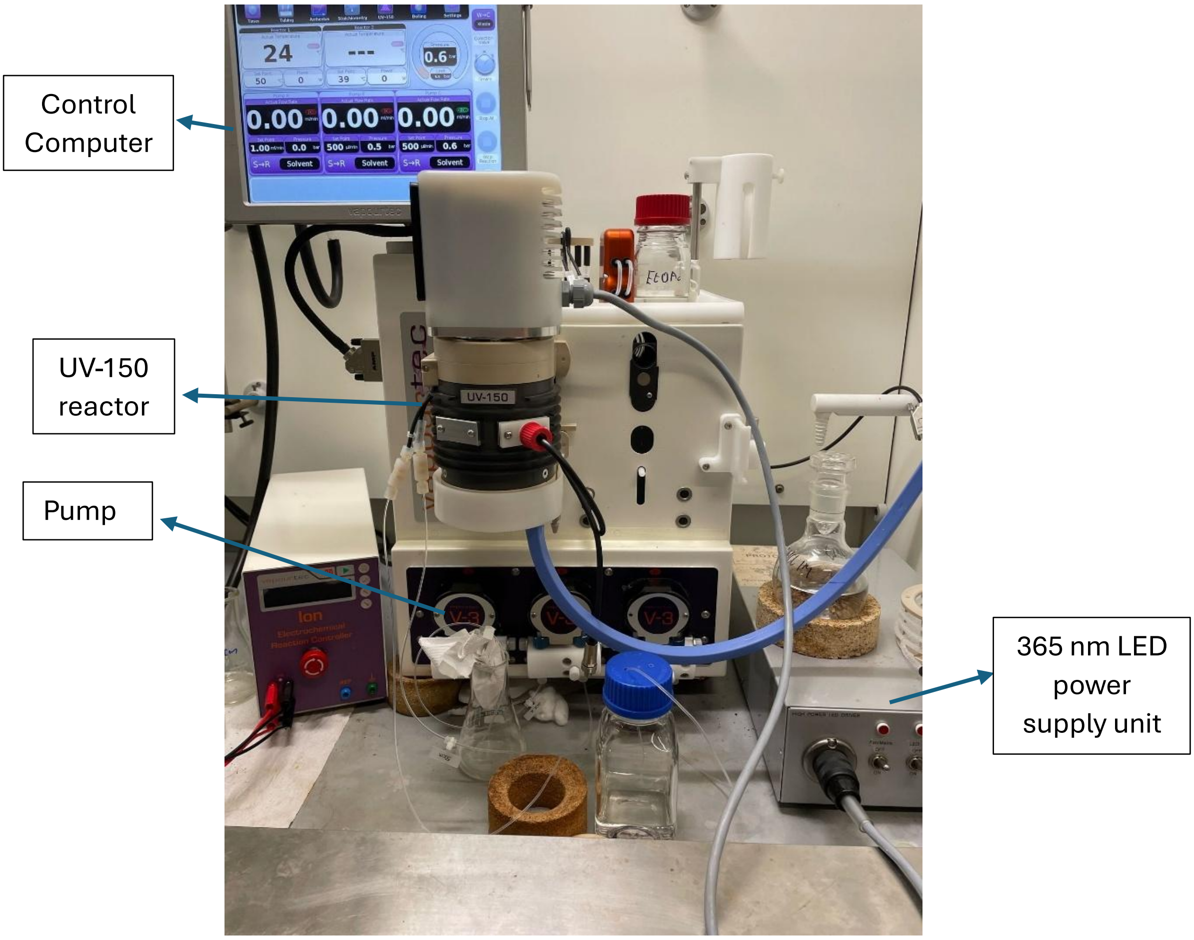

Figure S2: Image of flow reactor set-up.

3. Reaction Optimisations

3.1. Initial Parameter Screening

Initial optimisations began with a multi-factorial screening of stoichiometry, time and catalyst loading which can be seen below (**Table S1**) and summary of the process is shown (**Scheme S2**). These reactions were carried out at a concentration of 0.25 M in MeCN.

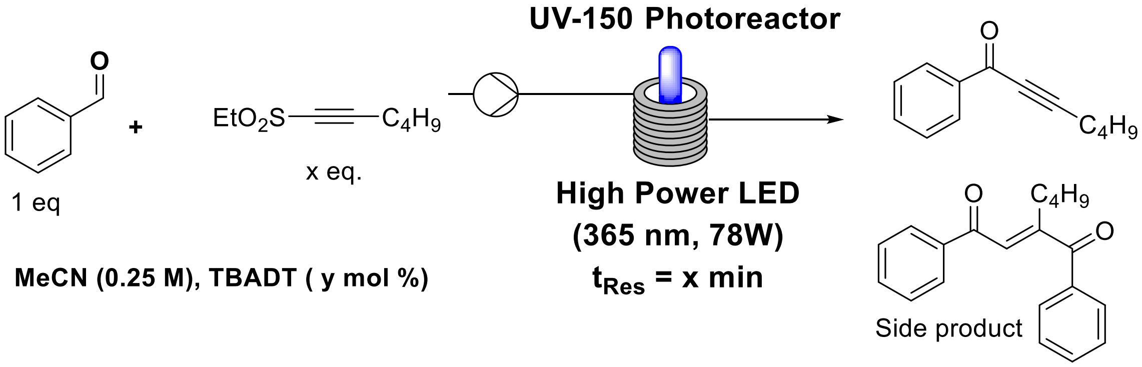

Scheme S2: *Summary of flow process for optimisation described in Table S1.*

Table S1: *Initial optimisation parameter screening*

Entry	Stoichiometry	Time (mins)	Catalyst loading (mol %)	%-Yield (Q-^{1}H-NMR)	Side product detected?
**1**	0.333	40	4	19	No
**2**	1.666	25	2.25	35	Yes
**3**	3	40	4	42	No
**4**	0.333	40	0.5	73	Yes
**5**	0.333	40	4	68	Yes
**6**	3	10	4	40	no

From this initial parameter screening a correlation matrix could be generated (**Table S1**) which is shown below. This matrix identifies how strongly impacting certain variables are on the overall reaction yield and guided further optimisations. This data shows that changes of the catalytic loading had a small impact on the reaction yield with a correlation coefficient of 2.31×10^{-2}, whilst stoichiometry and time had a much stronger impact with correlation coefficients of -5.22×10^{-1} and 2.36×10^{-1} respectively. Lastly, the negative number of stoichiometries coefficient indicates that increased equivalents of the sulfonated alkyne moiety had a negative impact on the reaction yield whilst catalyst and time both had a positive impact when increased.

Table S2: Correlation coefficient matrix for parameter screening

	Stoichiometry	*Time*	*Catalyst Loading (mol%)*
Stoichiometry	1	2.22×10^{-17}	-3.57×10^{-17}
Time	2.22×10^{-17}	1	-8.46×10^{-18}
Catalyst loading	-3.57×10^{-17}	-8.46×10^{-18}	1

With these results in hand, it was clear that increasing stoichiometric equivalents of the alkyne had a strongly negative impact on the reaction yield whilst increase time had a moderately positive impact and increasing catalytic loading had a minor impact. Resultantly a catalyst loading of 2 mol% was initially selected as this maintained a

relatively modest catalytic loading without requiring the reactions mass balance to be dominated by the catalyst due to its high molecular weight, despite being a low catalytic loading. Likewise, a stoichiometry of 0.333 equivalents of the alkyne moiety or 3 equivalents of the aldehyde was employed as not only had this given the highest yielding results in initial screen it also yielded the cleanest NMR spectra.

3.2. Suppression of Second Addition – Solvent Study

Initial purification of the target ynone proved challenging by flash chromatography, due to the co-elution of unwanted second addition products. The photochemistry of benzaldehyde is well documented with a tendency to independently form acyl radicals derived from benzoin. To combat this unproductive pathway acetone was added as a triplet quencher, promisingly a mixture of 1:1 MeCN:Acetone proved to inhibit further addition to the ynone products whilst only slightly reducing the overall reaction yield, providing a cleaner reaction mixture. A summary of this study can be seen below (**Fig S3**). This solvent study was conducted using 5 mol% of TBADT to best identify and prevent unwanted side product formation.

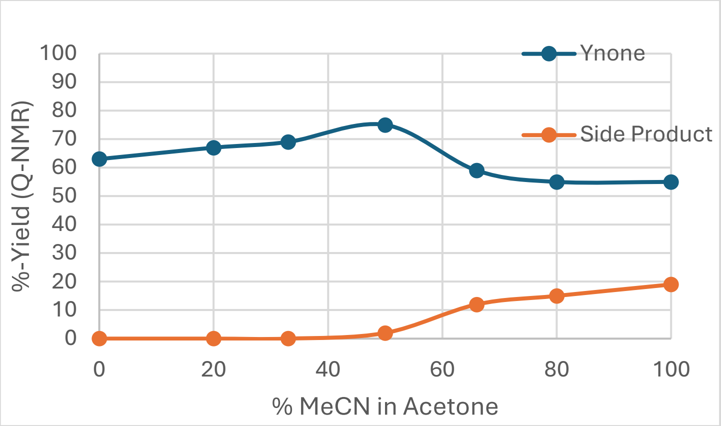

Figure S3: Solvent study investigating effect of acetone to inhibit side product formation.

3.3. Re-Evaluation of Optimization with New Solvent System

With a promising result demonstrating acetone could indeed supress subsequent additions of benzaldehyde to the ynone product concentration and reaction time were subsequently re-evaluated. When a 1:1 mixture of MeCN and acetone was utilised at lower catalytic loadings such as 0.5 mol% and 2 mol%, the reaction demonstrated reduced yields less than 50%. Therefore, the catalytic loading was returned to 5 mol% as it had been in the previous solvent study. Following the changes in catalyst loading and solvent system the reaction concentration and time was re-evaluated. The results of these studies can be seen below in **Table S3** and **Table S4**, respectively.

Table S3: *Concentration study utilising 1:1 mixture of MeCN to Acetone utilising 5 mol% TBADT loading, 3 equivalents of benzaldehyde and a reaction time of 30 minutes.*

Entry	Concentration (M)	% Yield (Q-NMR)
**1**	0.25	66
**2**	0.4	66
**3**	0.5	72
**4**	0.6	65
**5**	1	48

Table S4: *Switch off study utilising a 0.5 M (MeCN:Acetone, 1:1) solvent system, 5 mol % TBADT and 3 equivalents of benzaldehyde.*

Entry	Time (mins)	% Yield (Q-NMR)
**1**	0	0
**2**	10	37
**3**	15	58
**4**	20	65
**5**	25	68
**6**	30	70
**7**	35	74
**8**	40	77
**9**	45	68
**10**	50	52

Despite the addition of acetone causing an initial loss in the reaction yield, this could be recovered through an increase in the reaction time, affording a cleaner reaction mixture derived off a mixture of unconsumed starting material and product with only trace impurities. A summary of the optimal conditions and finalised flow process can be seen below (**Scheme S3**).

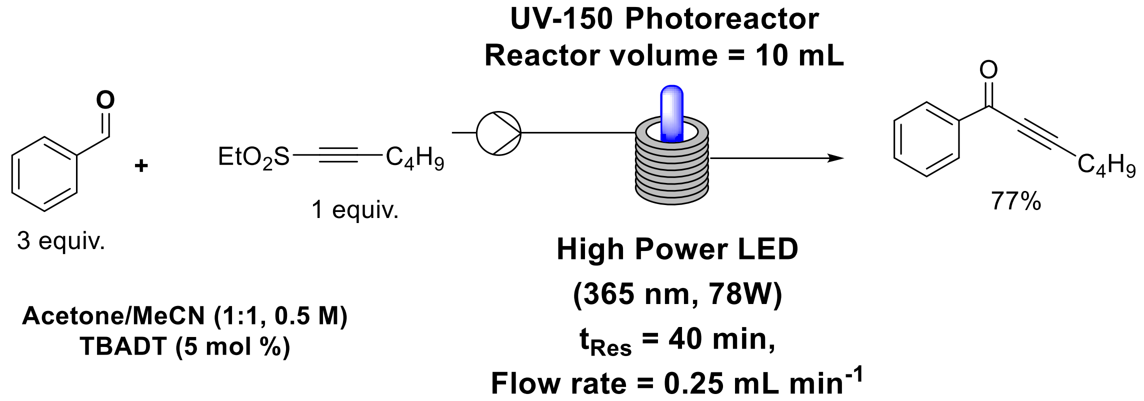

Scheme S3: Summary of finalised optimal conditions for the Giese-addition of benzaldehyde to sulfone substituted alkynes.

4. Characterization of Ynone Products

1-Phenylhept-2-yn-1-one, 3a:

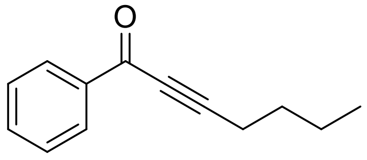

Chemical Formula: $C_{13}H_{14}O$
Molecular Weight: 186.25

Benzaldehyde (318 mg, 3 mmol), TBADT (83 mg, 0.05 equiv.) and 1-(ethylsulfonyl)hex-1-yne (188 mg, 1 mmol) were dissolved in a mixture of MeCN and acetone (1:1, 0.5 M, 2 mL). The resultant solution was pumped at a flow rate of 0.25 mL min^{-1} through a UV-150 reactor set-up (10 mL volume, PFA, 40 °C) equipped with a high-power LED emitting at 365 nm (input power 78 W). The title compound **3a** was isolated after purification using a PuriFlash preparatory chromatograph system employing a Interchim PF-15C18XS-F0012 column using a gradient elution system (MeCN/H_2O 9:1 – 1:9, 45 min duration) and subsequent extraction of the target product into Et_2O the target product was condensed *en vacuo* to afford the target with a 77% yield (148 mg, 0.77 mmol) as a pale-yellow oil.

^{1}H NMR (500 MHz, $CDCl_3$) δ 8.13 (m, 2H), 7.59 (t, *J* = 6.0 Hz, 1H), 7.47 (t, *J* = 6.0 Hz, 2H) 2.51 (t, *J* = 7 Hz, 2H), 1.67 (m , 2H), 1.50 (m, 2H), 0.97 (t, *J* = 7 Hz, 3H). $^{13}C\{^1H\}$ NMR (101 MHz, $CDCl_3$) δ 178.3 (C), 136.9 (C), 133.8 (CH), 129.5 (2CH), 128.5 (2CH), 96.8 (C), 79.7 (C), 29.8 (CH_2), 22.1 (CH_2), 18.9 (CH_2), 13.5 (CH_3). IR (neat) v/cm^{-1}: 3030 (w), 2931 (w), 2871 (w), 2236 (b), 2200 (m), 1640 (vs), 1448 (m), 1312 (m), 1262 (vs), 1174 (w), 909 (s), 698 (s). HR-MS (TOF-ES+) calc for $C_{13}H_{15}O$ 187.1117, found 187.1119 (M+H)+.

This data is consistent with published work [K. Ohe, et al., Org. Lett., 2014, 17, 23, 5843-5845].

1-(4-Methoxyphenyl)hept-2-yn-1-one, 3b:

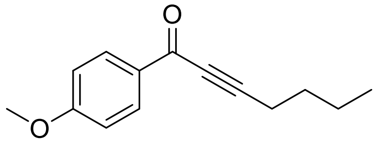

Chemical Formula: $C_{14}H_{16}O_2$
Molecular Weight: 216.28

4-Methoxybenzaldehyde (408 mg, 3 mmol). TBADT (83 mg, 0.05 equiv.) and 1-(ethylsulfonyl)hex-1-yne (188 mg, 1 mmol) were dissolved in a mixture of MeCN and acetone (1:1, 0.5 M, 2 mL). The resultant solution was pumped at a flow rate of 0.25 mL min^{-1} through a UV-150 reactor set-up (10 mL volume, PFA, 40 °C) equipped with a high-power LED emitting at 365 nm (input power 78 W). The title compound **3b** was isolated after purification using a PuriFlash preparatory chromatograph system employing a column containing 40 g of packed silica with an average diameter of 40 μm using 100% pentane to afford the target product with a 91% yield (197 mg, 0.91 mmol) as a pale-yellow oil.

^{1}H NMR (500 MHz, $CDCl_3$) δ 8.09 (d, *J* = 9 Hz, 2H), 6.93 (d, *J* = 9 Hz, 2H), 3.87 (s, 3H), 2.47 (t, *J* = 7 Hz, 2H), 1.70-1.58 (m, 2 H), 1.54-1.43 (m, 2H), 0.95 (t, *J* = 3 Hz). $^{13}C\{^1H\}$ NMR (101 MHz, $CDCl_3$) δ 177.1 (C), 164.4 (C), 132.0 (2CH), 130.5 (C), 113.8 (2CH), 96.0 (C), 79.9 (C), 55.6 (CH_3), 30.0 (CH_2), 22.2 (CH_2), 19.0 (CH_2), 13.6 (CH_3). IR (neat) v/cm^{-1}: 3074 (b), 3006 (w), 2933 (w), 2238 (m), 2199 (m), 1635 (s), 1593 (vs), 1420 (m), 1249 (vs), 1025 (m), 795 (m), 582 (m). HR-MS (TOF-ES+) calc for $C_{14}H_{17}O_2$ 217.1223, found 217.1225 (M+H)+.

This data is consistent with published work [Z. Gao, et al., Org Lett., 2015, 17, 13, 3298-3301.

1-(4-(Trifluoromethyl)phenyl)Hept-2-yn-1-one, 3c:

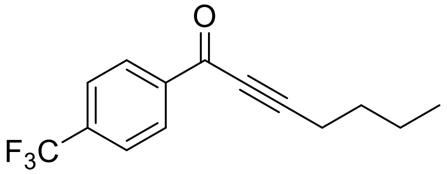

Chemical Formula: $C_{14}H_{13}F_3O$
Molecular Weight: 254.25

4-Trifluoromethylbenzaldehyde (522 mg, 3 mmol). TBADT (83 mg, 0.05 equiv.) and 1-(ethylsulfonyl) hex-1-yne (188 mg, 1 mmol) were dissolved in a mixture of MeCN and acetone (1:1, 0.5 M, 2 mL). The resultant solution was pumped at a flow rate of 0.25 mL min^{-1} through a UV-150 reactor set-up (10 mL volume, PFA, 40 °C) equipped with a high-power LED emitting at 365 nm (input power 78 W). The title compound **3c** was isolated after purification using a PuriFlash preparatory chromatograph system employing a column containing 40 g of packed silica with an average diameter of 40 µm using 100% pentane to afford the target product with an 59% yield (150 mg, 0.59 mmol) as a colourless oil.

^{1}H NMR (500 MHz, $CDCl_3$) δ 8.22 (d, *J* = 8 Hz, 2H), 7.73 (d, *J* = 8 Hz, 2H), 2.52 (t, *J* = 7 Hz, 2H), 1.67 (m, 2H), 1.50 (m, 2H), 0.96 (t, *J* = 7 Hz, 3H). $^{13}C\{^1H\}$ NMR (101 MHz, $CDCl_3$) δ 177.0 (C), 139.5 (C), 135.1 (q, 31 Hz, C), 129.9 (CH), 125.7 (q, *J* = 4 Hz, CH), 123.6 (q, *J* = 273 Hz, CF_3), 98.5 (C), 79.5 (C), 29.8 (CH_2), 22.2 (CH_2), 19.0 (CH_2), 13.6 (CH_3). $^{19}F\{^1H\}$ NMR (376 MHz, $CDCl_3$) δ -63.0 (3F). IR (neat) v/cm^{-1}: 3657 (b), 2299 (m), 2944 (b), 2293 (w), 2252 (vs), 1650 (m), 1443 (vs), 1413 (vs), 1069 (m), 918 (m), 749 (m). HR-MS (TOF-ES+) calc for $C_{14}H_{14}F_3O$ 255.0991, found 255.0993 (M+H)+.

This data is consistent with published work [X. Wu, Org. Biomol. Chem., 2014, 12, 5590-5593.]

1-(4-Chlorophenyl)hept-2-yn-1-one, 3d:

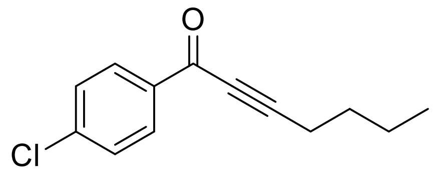

Chemical Formula: $C_{13}H_{13}ClO$
Molecular Weight: 220.70

4-Chlorobenzaldehyde (422 mg, 3 mmol), TBADT (83 mg, 0.05 equiv.) and 1-(ethylsulfonyl)hex-1-yne (188 mg, 1 mmol) were dissolved in a mixture of MeCN and acetone (1:1, 0.5 M, 2 mL). The resultant solution was pumped at a flow rate of 0.25 mL min^{-1} through a UV-150 reactor set-up (10 mL volume, PFA, 40 °C) equipped with a high-power LED emitting at 365 nm (input power 78 W). The title compound **3d** was isolated after purification using a PuriFlash preparatory chromatograph system employing a column containing 40 g of packed silica with an average diameter of 40 µm using 100% pentane to afford the target product with an 84% yield (185 mg, 0.84 mmol) as a colourless oil.

^{1}H NMR (600 MHz, $CDCl_3$) δ 8.07 (d, *J* = 9 Hz, 2H), 7.45 (d, *J* = 9 Hz, 2H), 2.51 (t, *J* = 7 Hz, 2H), 1.64 (p, *J* = 7 Hz, 2H), 1.5 (h, *J* = 7, 2H), 0.97 (t, *J* = 7 Hz, 3H). $^{13}C\{^1H\}$ NMR (151 MHz, $CDCl_3$) δ 176.9 (C), 140.46 (C), 135.4 (C), 130.9 (2CH), 128.9 (2CH), 97.5 (C), 79.4 (C), 29.8 (CH_2), 22.1 (CH_2), 18.9 (CH_2), 13.5 (CH_3). IR (neat) v/cm^{-1}: 3675 (b), 2959 (m), 2902

(m), 2237 (m), 2199 (m), 1646 (vs), 1585 (s), 1464 (m), 1306 (vs), 1067 (m), 843 (m), 646 (w). HR-MS (TOF-ES+) calc for $C_{13}H_{14}ClO$ 221.0728, found 221.0728 (M+H)+.

This data is consistent with published work [X. Wu, Org. Biomol. Chem., 2014, 12, 5590-5593].

1-(4-Bromophenyl)hept-2-yn-1-one, 3e:

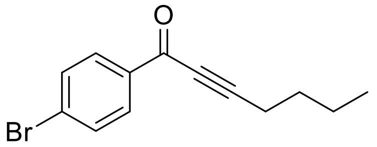

Chemical Formula: $C_{13}H_{13}BrO$
Molecular Weight: 265.15

4-Bromobenzaldehyde (555 mg, 3 mmol), TBADT (83 mg, 0.05 equiv.) and 1-(ethylsulfonyl)hex-1-yne (188 mg, 1 mmol) were dissolved in a mixture of MeCN and acetone (1:1, 0.5 M, 2 mL). The resultant solution was pumped at a flow rate of 0.25 mL min^{-1} through a UV-150 reactor set-up (10 mL volume, PFA, 40 °C) equipped with a high-power LED emitting at 365 nm (input power 78 W). The title compound **3e** was isolated after purification using a PuriFlash preparatory chromatograph system employing a column containing 40 g of packed silica with an average diameter of 40 µm using 100% pentane to afford the target product with an 59% yield (156mg, 0.59 mmol) as an orange oil.

^{1}H NMR (400 MHz, $CDCl_3$) δ 8.0 (d, *J* = 9 Hz, 2H), 7.6 (d, *J* = 9 Hz, 2H), 2.5 (t, *J* = 7 Hz, 2H), 1.66 (m, 2H), 1.5 (m, 2H), 0.96 (t, *J* = 7 Hz, 3H). ^{13}C{^{1}H} NMR (101 MHz, $CDCl_3$) δ 177.1 (C), 135.7 (C), 131.8 (2CH), 130.9 (2CH), 129.3 (C), 97.5 (C), 79.4 (C), 29.8 (CH_2), 22.1 (CH_2), 18.9 (CH_2), 13.5 (CH_3). IR (neat) v/cm^{-1}: 3668 (w), 3654 (w), 2932 (m), 2911 (m), 2231 (m), 2184 (m), 1632 (s), 1318 (m), 804 (w), 469 (w). HR-MS (TOF-ES+) calc for $C_{13}H_{14}BrO$ 266.0256, found 266.0256 (M+H)+.

This data is consistent with published work [X. Wu, Org. Biomol. Chem., 2014, 12, 5590-5593].

1-(4-(4,4,5,5-Tetramethyl-1,3,2-dioxaborolan-2-yl)phenyl)hept-2-yn-1-one, 3f:

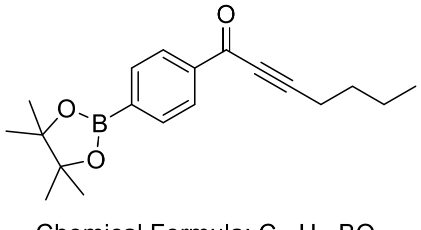

Chemical Formula: $C_{19}H_{25}BO_3$
Molecular Weight: 312.22

4-(4,4,5,5-Tetramethyl-1,3,2-dioxaborolan-2-yl)benzaldehyde (696 mg, 3 mmol), TBADT (83 mg, 0.05 equiv.) and 1-(ethylsulfonyl)hex-1-yne (188 mg, 1 mmol) were dissolved in a mixture of MeCN and acetone (1:1, 0.5 M, 2 mL). The resultant solution was pumped at a flow rate of 0.25 mL min^{-1} through a UV-150 reactor set-up (10 mL volume, PFA, 40 °C) equipped with a high-power LED emitting at 365 nm (input power 78 W). The title compound **3f** was isolated after purification using a PuriFlash preparatory chromatograph system employing a Interchim PF-15C18XS-F0012 column using a gradient elution system (MeCN/H_2O 9:1 – 1:9, 45 min duration) and subsequent extraction of the target product into Et_2O the target product was condensed *en. vacuo* to afford the target product with a 67% yield (209 mg, 0.67 mmol) as an orange oil.

^{1}H NMR (400 MHz, $CDCl_3$) δ 8.10 (d, *J* = 8 Hz, 2H), 7.90 (d, *J* = 8 Hz, 2H), 2.51 (t, *J* = 7 Hz, 2H), 1.66 (p, *J* = 7 Hz, 2H), 1.51 (m, 2H), 1.35 (s, 12H), 0.96 (t, *J* = 7 Hz, 3H). ^{13}C{^{1}H} NMR (101 MHz, $CDCl_3$) δ 178.4 (C), 138.8 (C), 134.8 (2CH), 128.5 (2CH), 97.1 (C), 84.2 (2C), 79.8 (C), 29.8 (CH_2), 24.9 ($4CH_3$), 22.1 (CH_2), 18.9 (CH_2), 13.5 (CH_3). IR (neat) v/cm^{-1}: 3660 (b), 3063 (w), 2957 (m), 2871 (m), 2145 (m), 2125 (w), 1714 (s), 1463 (s), 1178 (b), 1074 (w), 764 (s), 689 (s). HR-MS (TOF-ES+) calc for $C_{19}H_{26}BO_3$ 313.1973, found 313.1971 (M+H)+.

4-(Hept-2-ynoyl)phenyl acetate, 3g:

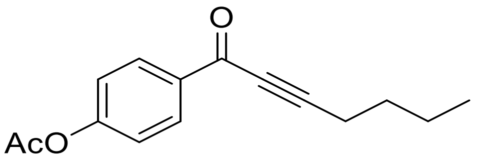

Chemical Formula: $C_{15}H_{16}O_3$
Molecular Weight: 244.2900

4-Formylphenyl acetate (492 mg, 3 mmol), TBADT (83 mg, 0.05 equiv.) and 1-(ethylsulfonyl)hex-1-yne (188 mg, 1 mmol) were dissolved in a mixture of MeCN and acetone (1:1, 0.5 M, 2 mL). The resultant solution was pumped at a flow rate of 0.25 mL min^{-1} through a UV-150 reactor set-up (10 mL volume, PFA, 40 °C) equipped with a high-power LED emitting at 365 nm (input power 78 W). The title compound **3g** was isolated after purification using a PuriFlash preparatory chromatograph system employing a column containing 40 g of packed silica with an average diameter of 40 μm using 100% pentane to afford the target product with a 92% yield (225 mg, 0.92 mmol) as a colourless oil.

^{1}H NMR (400 MHz, $CDCl_3$) δ 8.15 (d, *J* = 7.7 Hz, 2H), 7.20 (d, *J* = 10.0 Hz, 2H), 2.49 (t, *J* = 6.5 Hz, 2H), 2.32 (s, 3H), 1.72 – 1.59 (p, *J* = 7.3 Hz, 2H), 1.50 (p, *J* = 7.3 Hz, 2H), 0.95 (t, *J* = 7.3 Hz, 3H). ^{113}C{^{1}H} NMR (101 MHz, $CDCl_3$) δ 177.1 (C), 168.9 (C), 155.1 (C), 134.6 (C), 131.3 (2CH), 121.8 (2CH), 92.3 (C), 79.6 (C), 29.9 (CH_2), 22.2 (CH_3), 21.3 (CH_2), 19.0 (CH_2), 13.6 (CH_3). IR (neat) v/cm^{-1}: 3674 (w), 2959 (m), 2932 (w), 2238 (m), 2199 (m), 1759 (s), 1641 (s), 1500 (w), 1262 (m), 1182 (s), 1156 (s), 1087 (m), 754 (w), 578 (w). HR-MS (TOF-ES+) calc for $C_{15}H_{17}O_3$ 245.1173, found 245.1172 (M+H)+.

1-(Thiophen-2-yl)hept-2-yn-1-one, 3h:

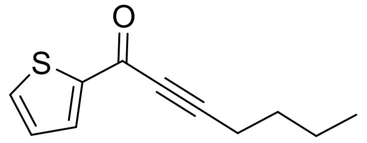

Chemical Formula: $C_{11}H_{12}OS$
Molecular Weight: 192.28

2-Thiophenecarboxyaldehyde (336 mg, 3 mmol), TBADT (83 mg, 0.05 equiv.) and 1-(ethylsulfonyl)hex-1-yne (188 mg, 1 mmol) were dissolved in a mixture of MeCN and acetone (1:1, 0.5 M, 2 mL). The resultant solution was pumped at a flow rate of 0.25 mL min^{-1} through a UV-150 reactor set-up (10 mL volume, PFA, 40 °C) equipped with a high-power LED emitting at 365 nm (input power 78 W). The title compound **3h** was isolated after purification using a PuriFlash preparatory chromatograph system employing a column containing 40 g of packed silica with an average diameter of 40 μm using 100% pentane to afford the target product with a 58% yield (111 mg, 0.58 mmol) as yellow oil.

^{1}H NMR (400 MHz, $CDCl_3$) δ 7.88 (dd, J = 3.8, 1.3 Hz, 1H), 7.66 (dd, J = 4.9, 1.2 Hz, 1H), 7.13 (dd, J = 4.9, 3.8 Hz, 1H), 2.47 (t, J = 7.0 Hz, 2H), 1.70 – 1.58 (m, 2H), 1.52 – 1.42 (m, 2H), 0.95 (t, J = 7.3 Hz, 3H). ^{13}C{^{1}H} NMR (101 MHz, $CDCl_3$) δ 170.2 (C), 145.2 (C), 134.9 (2xCH), 128.3 (CH), 95.4 (C), 79.4 (C), 29.9 (CH_2), 22.1 (CH_2), 18.9 (CH_2), 13.6 (CH_3). IR (neat) v/cm^{-1}: 2958 (m), 2932 (m), 2665 (m), 2228 (s), 2202 (m), 1620 (vs), 1514 (s), 1410 (vs), 1277 (s), 1035 (w), 727 (s). HR-MS (TOF-ES+) calc for $C_{11}H_{13}OS$ 193.0687, found 193.0685 (M+H)+.

This data is consistent with published work [S. Blum et al., Org. Lett., 2019, 21, 5, 1283-1286].

1-(1-Methyl-1H-indol-2-yl)hept-2-yn-1-one, 3i:

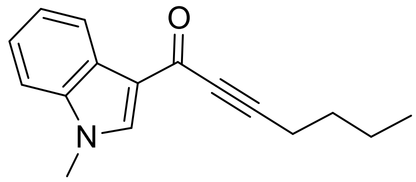

Chemical Formula: $C_{16}H_{17}NO$
Molecular Weight: 239.32

1-Methyl-1H-indole-3-carbaldehyde (477 mg, 3 mmol), TBADT (83 mg, 0.05 equiv.) and 1-(ethylsulfonyl)hex-1-yne (188 mg, 1 mmol) were dissolved in a mixture of MeCN and acetone (1:1, 0.5 M, 2 mL). The resultant solution was pumped at a flow rate of 0.25 mL min^{-1} through a UV-150 reactor set-up (10 mL volume, PFA, 40 °C) equipped with a high-power LED emitting at 365 nm (input power 78 W). The title compound **3i** was isolated after purification using a PuriFlash preparatory chromatograph system employing a column containing 40 g of packed silica with an average diameter of 40 μm using 100% pentane to afford the target product with a 22% yield (53 mg, 0.22 mmol) as a brown oil.

^{1}H NMR (500 MHz, $CDCl_3$) δ 8.39 – 8.36 (m, 1H), 7.86 (s, 1H), 7.34 – 7.28 (m, 3H), 3.86 (s, 3H), 2.46 (t, J = 8.0 Hz, 2H), 1.68 – 1.62 (m, 2H), 1.55 – 1.46 (m, 2H), 0.96 (t, J = 8.0 Hz, 3H). ^{13}C{^{1}H} NMR (126 MHz, $CDCl_3$) δ 171.8 (C), 138.8 (CH), 137.8 (C), 125.9 (C), 123.8 (CH), 123.0 (CH), 122.6 (CH), 118.5 (C), 109.8 (CH), 90.8 (C), 80.7 (C), 33.7 (CH_3), 30.2 (CH_2), 22.2 (CH_2), 18.9 (CH_2), 13.7 (CH_3). IR (neat) v/cm^{-1}: 3674 (w), 3666 (w), 297 (vs), 2929 (vs), 2224 (b), 2194 (b), 1604 (s), 1524 (s), 1250 (m), 1222 (m), 1109 (s), 747 (m). HR-MS (TOF-ES+) calc for $C_{16}H_{18}NO$ 240.1383, found 240.1384 (M+H)+.

1-Cyclohexylhept-2-yn-1-one, 3j:

Cyclohexanecarboxyaldehyde (336 mg, 3 mmol), 1-(ethylsulfonyl)hex-1-yne (188 mg, 1 mmol) were dissolved in a mixture of MeCN and acetone (1:1, 0.5 M, 2 mL). The resulting solution was pumped at a flow rate of 0.25 mL min^{-1} through a UV-150 reactor set-up (10 mL volume, PFA, 40 °C) equipped with a high-power LED emitting at 365 nm (input power 78 W). The title compound **3j** was isolated after purification using a PuriFlash preparatory chromatograph system employing a column containing 40 g of packed silica with an

average diameter of 40 μm using 100% pentane to afford the target product with a 68% yield (131 mg, 0.68 mmol) as colourless oil.

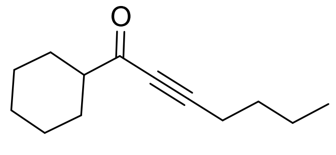

Chemical Formula: $C_{13}H_{20}O$
Molecular Weight: 192.3020

^{1}H NMR (400 MHz, $CDCl_3$) δ 2.38 (t, *J* = 7 Hz, 3H), 1.98-1.94 (m, 2H), 1.81-1.75 (m, 2H), 1.60-1.54 (m, 3H), 1.49-1.40 (m, 3H), 1.34-1.21 (m, 4H), 0.94 (t, *J* = 7 Hz, 3H). $^{13}C\{^1H\}$ NMR (126 MHz, $CDCl_3$) δ 191.8 (C), 94.9 (C), 80.2 (C), 52.3 (CH), 29.8 (CH_2), 28.3 ($2CH_2$), 25.8 (CH_2), 25.4 ($2CH_2$), 21.9 (CH_2), 18.7 (CH_2), 13.5 (CH_3). IR (neat) ν/cm^{-1}: 2987 (vs), 2857 (m), 2142 (m), 2132 (m), 1737 (s), 1704 (s), 1449 (m), 1250 (m), 1075 (s), 801 (m). HR-MS (TOF-ES+) calc for $C_{13}H_{21}O$ 193.1587, found 193.1587 (M+H)+.

This data is consistent with published work [F. Tsai, Tetrahedron, 2009, 65, 49, 10134-10141].

1-(4-Methoxyphenyl)-3-phenylprop-2-yn-1-one, 4a:

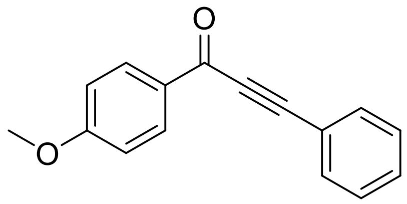

Chemical Formula: $C_{16}H_{12}O_2$
Molecular Weight: 236.27

4-Methoxy-benzaldehyde (408 mg, 3 mmol). TBADT (83 mg, 0.05 equiv.) and ((ethylsulfonyl)ethynyl)benzene (194 mg, 1 mmol) were dissolved in a mixture of MeCN and acetone (1:1, 0.5 M, 2 mL). The resultant solution was pumped at a flow rate of 0.25 mL min^{-1} through a UV-150 reactor set-up (10 mL volume, PFA, 40 °C) equipped with a high-power LED emitting at 365 nm (input power 78 W). The title compound **4a** was isolated after purification using a PuriFlash preparatory chromatograph system employing a column containing 40 g of packed silica with an average diameter of 40 μm using 100% pentane to afford the target product with a 65% yield (154 mg, 0.65 mmol) as yellow oil.

^{1}H NMR (400 MHz, $CDCl_3$) δ 8.19 (d, *J* = 8 Hz, 2H), 7.67 (dd, *J* = 8, 2 Hz, 2H), 7.52 – 7.36 (m, 3H), 6.98 (d, *J* = 9.0 Hz, 2H), 3.89 (s, 3H). $^{13}C\{^1H\}$ NMR (101 MHz, $CDCl_3$) δ 176.8 (C), 164.6 (C), 133.0 (2CH), 132.1 (2CH), 130.7 (CH), 130.4 (C), 128.7 (2CH), 120.5 (C), 114.0 (2CH), 92.4 (C), 87.0 (C), 55.7 (CH_3). IR (neat) ν/cm^{-1}: 3010 (m), 2958 (m), 2901 (b), 2197 (vs), 1628 (vs), 1600 (s), 1305 (s), 1011 (b), 840, 685 (m). HR-MS (TOF-ES+) calc for $C_{16}H_{13}O_2$ 237.0910, found 237.0914 (M+H)+.

This data is consistent with published work [Z. Gao, et al., Org. Lett., 2015, 17, 13, 3298-3301.

1,3-Diphenylprop-2-yn-1-one, 4b:

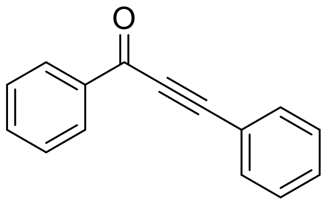

Chemical Formula: $C_{15}H_{10}O$
Molecular Weight: 206.24

Benzaldehyde (318 mg, 3 mmol). TBADT (83 mg, 0.05 equiv.) and ((ethylsulfonyl)ethynyl)benzene (194 mg, 1 mmol) were dissolved in a mixture of MeCN and acetone (1:1, 0.5 M, 2 mL). The resultant solution was pumped at a flow rate of 0.25 mL min^{-1} through a UV-150 reactor set-up (10 mL volume, PFA, 40 °C) equipped with a high-power LED emitting at 365 nm (input power 78 W). The title compound **4b** was isolated after purification using a PuriFlash preparatory chromatograph system employing a column containing 40 g of packed silica with an average diameter of 40 µm using 100% pentane to afford the target product with a 75% yield (155 mg, 0.75 mmol) as yellow oil.

^{1}H NMR (400 MHz, $CDCl_3$) δ 8.23 (dd, *J* = 8.4 Hz, 1.3 Hz, 2H), 7.71-7.67 (m, 2H), 7.66-7.60 (m, 1H), 7.55-7.46 (m, 3H), 7.45-7.39 (m, 2H). ^{13}C{^{1}H} NMR (101 MHz, $CDCl_3$) δ 178.0 (C), 136.9 (C), 134.1 (CH), 133.1 (2CH), 130.8 (CH), 129.6 (2CH), 128.7 (2CH), 128.6 (2CH), 120.2 (C), 93.1 (C), 86.9 (C). IR (neat) v/cm^{-1}: 3059 (w), 2970 (w), 2901 (w), 2195 (vs), 1636 (vs), 1596 (m), 1447 (m), 1207 (m), 993 (m), 685 (s). HR-MS (TOF-ES+) calc for $C_{15}H_{11}O$ 207.0804, found 207.0806 (M+H)+.

This data is consistent with published work [Z. Gao, et al., Org Lett., 2015, 17, 13, 3298-3301]

3-Phenyl-1-(4-(trifluoromethyl)phenyl)prop-2-yn-1-one, 4c:

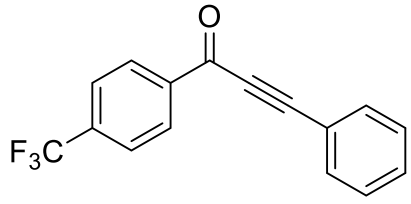

Chemical Formula: $C_{16}H_9F_3O$
Molecular Weight: 274.24

4-Trifluormethylbenzaldehyde (522 mg, 3 mmol). TBADT (88 mg, 0.05 equiv.) and ((ethylsulfonyl)ethynyl)benzene (194 mg, 1 mmol) were dissolved in a mixture of MeCN and acetone (1:1, 0.5 M, 2 mL). The resultant solution was pumped at a flow rate of 0.25 mL min^{-1} through a UV-150 reactor set-up (10 mL volume, PFA, 40 °C) equipped with a high-power LED emitting at 365 nm (input power 78 W). The title compound **4c** was isolated after purification using a PuriFlash preparatory chromatograph system employing a column containing 40 g of packed silica with an average diameter of 40 µm using 100% pentane to afford the target product with a 58% yield (159 mg, 0.58 mmol) as an orange oil.

^{1}H NMR (400 MHz, $CDCl_3$) δ 8.32 (d, *J* = 8.1 Hz, 2H), 7.78 (d, *J* = 8.1 Hz, 2H), 7.75 – 7.64 (m, 2H), 7.55-7.48 (m, 1H), 7.44 (t, 7.0 Hz, 2H). ^{13}C{^{1}H} NMR (101 MHz, $CDCl_3$) δ 176.8 (C), 139.5 (C), 135.3 (q, *J* = 33 Hz, C), 133.3 (2CH), 131.3 (CH), 129.9 (2CH), 128.9 (2CH), 125.8 (2CH), 123.6 (q, *J* = 273 Hz, CF_3), 119.8 (C), 94.6 (C), 86.7 (C).^{19}F{^{1}H} NMR (376 MHz, $CDCl_3$) δ -63.0 (3F). IR (neat) v/cm^{-1}: 2971 (m), 2901 (m), 2199 (s), 1641 (s), 1490 (w), 1232

(s), 1162 (s), 1064 (s), 1029 (vs), 801 (m), 687 (m). HR-MS (TOF-ES+) calc for $C_{16}H_{10}F_3O$ 275.0678, found 275.0678 (M+H)+.

3-Cyclopropyl-1-phenylprop-2-yn-1-one, 4d:

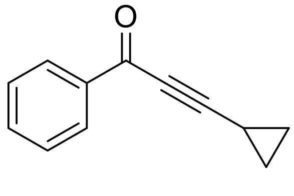

Chemical Formula: $C_{12}H_{10}O$
Molecular Weight: 170.21

Benzaldehyde (318 mg, 3 mmol). TBADT (88 mg, 0.05 equiv.) and ((ethylsulfonyl)ethynyl)cyclopropane (194 mg, 1 mmol) were dissolved in a mixture of MeCN and acetone (1:1, 0.5 M, 2 mL). The resultant solution was pumped at a flow rate of 0.25 mL min^{-1} through a UV-150 reactor set-up (10 mL volume, PFA, 40 °C) equipped with a high-power LED emitting at 365 nm (input power 78 W). The title compound **4d** was isolated after purification using a PuriFlash preparatory chromatograph system employing a column containing 40 g of packed silica with an average diameter of 40 µm using 100% pentane to afford the target product with a 53% yield (90 mg, 0.53 mmol) as colourless oil.

^{1}H NMR (400 MHz, $CDCl_3$) δ 8.09 (d, *J* = 8.5 Hz, 2H), 7.58 (t, *J* = 7.4 Hz, 1H), 7.46 (t, *J* = 7.6 Hz, 2H), 1.58 – 1.49 (m, 1H), 1.11 – 0.97 (m, 4H). ^{13}C{^{1}H} NMR (101 MHz, $CDCl_3$) δ 178.0 (C), 137.1 (C), 133.8 (CH), 129.5 (2CH), 128.5 (2CH), 101.1 (C), 75.6 (C), 9.97 (CH), 0.10 ($2CH_2$). IR (neat) v/cm^{-1}: 2987 (m), 2922 (m), 2901 (b), 2210 (vs), 1638 (vs), 1597 (w), 1939 (w), 1267 (s), 1074 (b), 915 (s) 700 (m). HR-MS (TOF-ES+) calc for $C_{12}H_{11}O$ 171.0804, found 171.0806 (M+H)+.

This data is consistent with published work [S. Blum et al., Org. Lett., 2019, 21, 5, 1283-1286].

1-Phenylnon-1-yn-3-one, 4e:

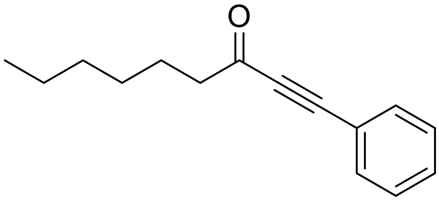

Chemical Formula: $C_{15}H_{18}O$
Molecular Weight: 214.31

Heptanal (343 mg, 3 mmol). TBADT (88 mg, 0.05 equiv.) and ((ethylsulfonyl)ethynl)benzene (194 mg, 1 mmol) were dissolved in a mixture of MeCN and acetone (1:1, 0.5 M, 2 mL). The resultant solution was pumped at a flow rate of 0.25 mL min^{-1} through a UV-150 reactor set-up (10 mL volume, PFA, 40 °C) equipped with a high-power LED emitting at 365 nm (input power 78 W). The title compound **4e** was isolated after purification using a PuriFlash preparatory chromatograph system employing a column containing 40 g of packed silica with an average diameter of 40 µm using C-Hex:EtOAc, 1:1 to afford the target product with a 50% yield (107 mg, 0.50 mmol) as pale-yellow oil.

^{1}H NMR (400 MHz, $CDCl_3$) δ 7.57 (d, *J* = 8.4 Hz, 2H), 7.46 (t, *J* = 7.6 Hz, 1H), 7.38 (t, *J* = 7.6 Hz, 2H), 2.66 (t, *J* = 8.0 Hz, 2H), 1.74 (p, *J* = 8.0 Hz, 2H), 1.38-1.30 (m, 6H), 0.89 (t, *J* = 7.8

Hz, 3H). $^{13}C\{^1H\}$ NMR (101 MHz, $CDCl_3$) δ 133.0 (2CH), 130.6 (CH), 128.6 (CH), 120.1 (C), 90.5 (C), 87.9 (C), 45.6 (CH_2), 31.5 (CH_2), 28.7 (CH_2), 24.1 (CH_2), 22.5 (CH_2), 14.0 (CH_3). IR (neat) v/cm^{-1}: 2991 (vs), 2862 (m), 2149 (m), 2137 (m), 1743 (s), 1722 (s), 1451 (s), 1248 (m), 1072 (s), 801 (m).

This data is consistent with published work [Y. Hu et al., Chem. Comm., 2018, 54, 9517-9520].

5. Characterization of Alkyne Adducts

(Hex-1-yn-1-ylsulfonyl)benzene, 5a:

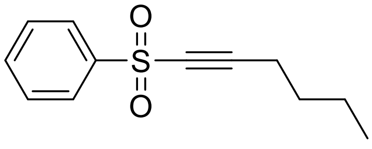

Chemical Formula: $C_{12}H_{14}O_2S$
Molecular Weight: 222.30

Compound **5a** was prepared as outlined in general synthesis procedure 2.1, using 1-hexyne (5.2 mmol) and diphenyl disulfide (5 mmol) affording the target compound in a 68% yield over the two steps as a pale-yellow oil (3.4 mmol, 755 mg).

^1H NMR (400 MHz, $CDCl_3$) δ 7.99 (d, *J* = 8.4 Hz, 2H), 7.75 – 7.62 (m, 1H), 7.60 – 7.46 (m, 2H), 2.35 (t, *J* = 7.1 Hz, 2H), 1.70 – 1.47 (m, 2H), 1.41 – 1.22 (m, 2H), 0.87 (t, *J* = 6.2 Hz, 3H). $^{13}C\{^1H\}$ NMR (101 MHz, $CDCl_3$) δ 142.1 (C), 134.0 (CH), 129.3 (2CH), 127.2 (2CH), 98.0 (C), 78.2 (C), 29.0 (CH_2), 22.0 (CH_2), 18.7 (CH_2), 13.4 (CH_3). IR (neat) v/cm^{-1}: 2960 (m), 2933 (m), 2873 (b), 2198 (s), 1447 (s), 1156 (vs), 1070 (s), 725 (s). HR-MS (TOF-ES+) calc for $C_{12}H_{15}O_2S$ 223.0793, found 223.0793 (M+H)+.

This data is consistent with published work [L. Zhang, Angew. Chem. Int. Ed., 2015, 54, 40, 11775-11779].

((Phenylethynyl)sulfonyl)benzene, 5b:

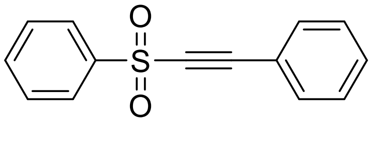

Chemical Formula: $C_{14}H_{10}O_2S$
Molecular Weight: 242.29

Compound **5b** was prepared as outlined in general synthesis procedure **2.1**, using phenylacetylene (5.2 mmol) and diphenyl disulfide (5 mmol) affording the target compound in a 62% yield over the two steps as a pale-yellow solid (3.1 mmol, 751 mg).

^1H NMR (500 MHz, $CDCl_3$) δ 8.18 – 8.02 (d, *J* = 8 Hz, 2H), 7.69 (t, *J* = 8 Hz, 1H), 7.61 (d, *J* = 8 Hz, 2H), 7.53 (d, *J* = 8 Hz, 2H), 7.47 (t, *J* = 8 Hz, 1H), 7.37 (t, *J* = 8 Hz, 2H). $^{13}C\{^1H\}$ NMR (126 MHz, $CDCl_3$) δ 141.8 (C), 134.2 (CH), 132.8 (2CH), 131.6 (CH), 129.4 (2CH), 128.7 (CH), 127.4 (2CH), 117.9 (C), 93.5 (C), 85.3 (C). IR (neat) v/cm^{-1}: 2952 (m), 2841 (m), 2197

(s), 1432 (s), 1379 (m), 1146 (s), 1064 (s), 728 (s). HR-MS (TOF-ES+) calc for $C_{14}H_{11}O_2S$ 243.0474, found 243.0476 (M+H)+.

This data is consistent with published work [C. Kuhakarn, J. Org. Chem., 2016, 81, 7, 2744-2752].

1-(Hex-1-yn-1-ylsulfonyl)-4-methylbenzene, 6a:

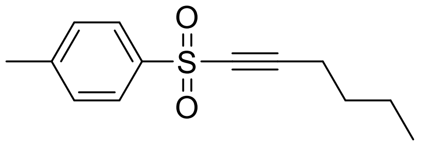

Chemical Formula: $C_{13}H_{16}O_2S$
Molecular Weight: 236.33

Compound **6a** was prepared as outlined in general synthesis procedure **2.1**, using 1-hexyne (5.2 mmol) and di-p-tolyl disulfide (5 mmol) affording the target compound in a 54% yield over the two steps as an yellow oil (2.7 mmol, 638 mg).

^{1}H NMR (400 MHz, $CDCl_3$) δ 7.86 (d, *J* = 8.4 Hz, 2H), 7.34 (d, *J* = 8.4 Hz, 2H), 2.44 (s, 3H), 2.34 (t, *J* = 7.1 Hz, 2H), 1.53 – 1.46 (m, 2H), 1.42 – 1.28 (m, 2H), 0.87 (t, *J* = 7.3 Hz, 3H). ^{13}C{^{1}H} NMR (101 MHz, $CDCl_3$) δ 145.1 (C), 139.3 (C), 129.9 (2CH), 127.3 (2CH), 97.4 (C), 78.5 (C), 29.1 (CH_2), 21.10 (CH_2), 21.8 (CH_3), 18.7 (CH_2), 13.4 (CH_3). IR (neat) v/cm^{-1}: 2960 (m), 2851 (m), 2198 (s), 1586 (s), 1447 (s), 1392 (m), 1156 (vs), 1070 (s), 725 (s).HR-MS (TOF-ES+) calc for $C_{13}H_{17}O_2S$ 237.0945, found 237.0944 (M+H)+.

This data is consistent with published work [B. König, ChemPhotoChem, 2017, 1, 5, 327-242].

1-Methyl-4-((phenylethynyl)sulfonyl)benzene, 6b:

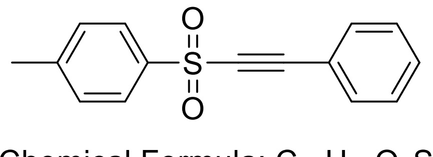

Chemical Formula: $C_{15}H_{12}O_2S$
Molecular Weight: 256.32

Compound **6b** was prepared as outlined in general synthesis procedure **2.1**, using Phenylacetylene (5.2 mmol) and di-p-tolyl disulfide (5 mmol) affording the target compound in a 61% yield over the two steps as a white crystalline solid (3.05 mmol, 781 mg).

^{1}H NMR (500 MHz, $CDCl_3$) δ 7.96 (d, *J* = 8.4 Hz, 2H), 7.52 (dd, *J* = 8.4, 1.3 Hz, 2H), 7.48 – 7.44 (m, 1H), 7.42 – 7.31 (m, 4H), 2.47 (s, 3H). ^{13}C{^{1}H} NMR (126 MHz, $CDCl_3$) δ 145.4 (C), 139.0 (C), 132.7 (2CH), 131.4 (CH), 123.0 (2CH), 128.7 (2CH), 127.5 (2CH), 118.0 (C), 93.0 (C), 85.6 (C), 21.7 (CH_3). IR (neat) v/cm^{-1}: 2958 (m), 2849 (m), 2201 (s), 1439 (s), 1381 (m), 1148 (s), 1065 (s), 722 (s). HR-MS (TOF-ES+) calc for $C_{15}H_{13}O_2S$ 257.0631, found 257.0632 (M+H)+.

This data is consistent with published work [B. König, ChemPhotoChem, 2017, 1, 5, 327-242.].

1-(Methylsulfonyl)hex-1-yne, 7a:

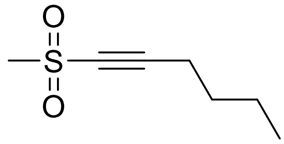

Chemical Formula: $C_7H_{12}O_2S$
Molecular Weight: 160.23

Compound **7a** was prepared as outlined in general synthesis procedure **2.1**, using 1-hexyne (5 mmol) and di-methyl disulfide (5.2 mmol) affording the target compound in a 48% yield over the two steps as a colourless oil (2.4 mmol, 384 mg).

^{1}H NMR (400 MHz, $CDCl_3$) δ 3.17 (s, 3H), 2.40 (t, *J* = 7.1 Hz, 2H), 1.73 – 1.51 (m, 2H), 1.50 – 1.35 (m, 2H), 0.92 (t, *J* = 7.3 Hz, 3H). ^{13}C{^{1}H} NMR (101 MHz, $CDCl_3$) δ 95.8 (C), 77.3 (C), 46.8 (CH_3), 29.0 (CH_2), 22.0 (CH_2), 18.5 (CH_2), 13.5 (CH_3). IR (neat) v/cm^{-1}: 2960 (m), 2932 (m), 2874 (b), 2202 (m), 1717 (m), 1307 (vs), 1144 (s), 965 (m), 771 (m). HR-MS (TOF-ES+) calc for $C_7H_{13}O_2S$ 161.0631, found 161.0631 (M+H)+.

This data is consistent with published work [L. Capaldo, D. Ravelli, Org. Lett., 2021, 23, 6, 2243-2247]

((Methylsulfonyl)ethynyl)benzene, 7b:

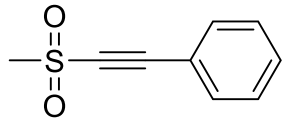

Chemical Formula: $C_9H_8O_2S$
Molecular Weight: 180.22

Compound **7b** was prepared as outlined in general synthesis procedure **2.1**, using phenylacetylene (5.2 mmol) and di-methyl disulfide (5 mmol) affording the target compound in a 63% yield over the two steps as a yellow solid (3.2 mmol, 577 mg).

^{1}H NMR (400 MHz, $CDCl_3$) δ 7.59 (d, *J* = 7.2 Hz, 2H), 7.52 (t, *J* = 7.5 Hz, 1H), 7.42 (t, *J* = 7.5 Hz, 2H), 3.30 (s, 3H). ^{13}C{^{1}H} NMR (101 MHz, $CDCl_3$) δ 132.9 (2CH), 131.8 (CH), 128.8 (2CH), 117.5 (C), 91.5 (C), 84.4 (C), 46.8 (CH_3). IR (neat) v/cm^{-1}: 3071 (b), 2970 (b), 2890 (b), 2184 (w), 1685 (vs), 1324 (s), 1291 (w), 1072 (m), 933 (b), 707 (s). HR-MS (TOF-ES+) calc for $C_9H_9O_2S$ 181.0318, found 181.0318 (M+H)+.

This data is consistent with published work [L. Capaldo, D. Ravelli, Org. Lett., 2021, 23, 6, 2243-2247]

1-(Ethylsulfonyl)hex-1-yne, 8a:

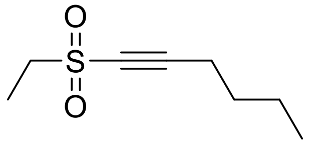

Chemical Formula: $C_8H_{14}O_2S$
Molecular Weight: 174.26

Compound **8a** was prepared as outlined in general synthesis procedure **2.1**, using 1-hexyne (5.2 mmol) and di-ethyl disulfide (5 mmol) affording the target compound in an 81% yield over the two steps as a colourless oil (4.1 mmol, 705 mg).

^{1}H NMR (400 MHz, $CDCl_3$) δ 3.15 (q, *J* = 7.4, 2H), 2.40 (t, *J* = 7.1 Hz, 2H), 1.57 (p, *J* = 7.1 Hz, 2H), 1.49 – 1.34 (m, 5H), 0.91 (t, *J* = 7.3 Hz, 3H). $^{13}C\{^1H\}$ NMR (101 MHz, $CDCl_3$) δ 96.7 (C), 75.5 (C), 52.6 (CH_2), 29.1 (CH_2), 22.0 (CH_2), 18.5 (CH_2), 13.5 (CH_3), 7.8 (CH_3). IR (neat) v/cm^{-1}: 2961 (b), 2935 (b), 2875 (b), 2199 (s), 1457 (m), 1319 (s), 1139 (s), 780 M). HR-MS (TOF-ES+) calc for $C_8H_{15}O_2S$ 175.0787, found 175.0788 (M+H)+.

((Ethylsulfonyl)ethynyl)benzene, 8b:

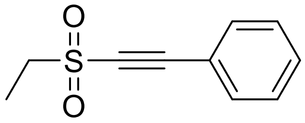

Chemical Formula: $C_{10}H_{10}O_2S$
Molecular Weight: 194.25

Compound **8b** was prepared as outlined in general synthesis procedure **2.1**, using phenylacetylene (5.2 mmol) and di-ethyl disulfide (5 mmol) affording the target compound in an 78% yield over the two steps as a pale-yellow oil (3.9 mmol, 758 mg).

^{1}H NMR (400 MHz, $CDCl_3$) δ 7.58 (d, *J* = 8.1 Hz, 2H), 7.54 – 7.47 (m, 1H), 7.40 (t, *J* = 6.8 Hz, 2H), 3.35 – 3.23 (q, *J* = 4 Hz, 2H), 1.53 (t, *J* = 4 Hz, 3H). $^{13}C\{^1H\}$ NMR (101 MHz, $CDCl_3$) δ 133.0 (2CH), 131.8 (CH), 128.9 (2CH), 117.7 (C), 92.6 (C), 82.8 (C), 52.9 (CH_2), 7.8 (CH_3). IR (neat) v/cm^{-1}: 2986 (m), 2941 (b), 2901 (b), 2179 (s), 1489 (w), 1328 (vs), 1137 (vs), 1044 (m), 844 (m), 756 (s), 493 (s). HR-MS (TOF-ES+) calc for $C_{10}H_{11}O_2S$ 195.0474, found 195.0474 (M+H)+.

This data is consistent with published work [J. Wu, Org., et al., Chem. Front., 2020, 7, 938-943]

1-(Isopropylsulfonyl)hex-1-yne, 9a:

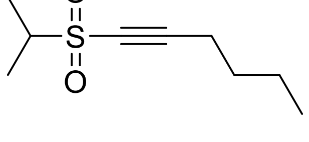

Chemical Formula: $C_9H_{16}O_2S$
Molecular Weight: 188.29

Compound **9a** was prepared as outlined in general synthesis procedure **2.1**, using 1-hexyne (5.2 mmol) and di-isopropyl disulfide (5 mmol) affording the target compound in an 32% yield over the two steps as a colourless oil (1.6 mmol, 300 mg).

^{1}H NMR (400 MHz, $CDCl_3$) δ 3.05 (sept, *J* = 6.8, 1H), 2.39 (t, *J* = 7.1 Hz, 2H), 1.64 – 1.46 (m, 2H), 1.45 – 1.32 (m, 5H), 1.28 (d, *J* = 6.8 Hz, 3H), 0.87 (t, *J* = 7.3Hz, 3H). $^{13}C\{^{1}H\}$ NMR (101 MHz, $CDCl_3$) δ 105.9 (C), 74.9 (C), 54.7 (CH), 29.6 (CH_2), 21.8 (CH_2), 19.3 (CH_2), 15.1 (CH_3), 14.8 (CH_3), 13.4 (CH_3). IR (neat) v/cm^{-1}: 2987 (b), 2942 (b), 2901 (b), 2193 (s), 1315 (vs), 1131 (vs), 1043 (m), 833 (m), 726 (s), 633 (m). HR-MS (TOF-ES+) calc for $C_9H_{17}O_2S$ 189.0944, found 189.0944 ($M+H^+$).

((Isopropylsulfonyl)ethynyl)benzene, 9b:

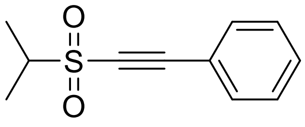

Chemical Formula: $C_{11}H_{12}O_2S$
Molecular Weight: 208.27

Compound **9b** was prepared as outlined in general synthesis procedure **2.1**, using phenylacetylene (22 mmol) and di-isopropyl disulfide (20 mmol) affording the target compound in an 58% yield over the two steps as a colourless oil (11.6 mmol, 2.42 g).

^{1}H NMR (400 MHz, $CDCl_3$) δ 7.61 – 7.55 (m, 2H), 7.50 (t, *J* = 7.6 Hz, 1H), 7.40 (t, *J* = 7.6 Hz, 2H), 3.29 (sept, *J* = 6.8 Hz, 1H), 1.50 (d, *J* = 6.8 Hz, 6H). $^{13}C\{^{1}H\}$ NMR (101 MHz, $CDCl_3$) δ 132.9 (2CH), 131.7 (CH), 128.8 (2CH), 117.8 (C), 93.3 (C), 81.3 (C), 57.6 (CH), 15.7 (2CH_3). IR (neat) v/cm^{-1}: 3061 (b), 2909 (b), 2179 (s), 1690 (w), 1445 (m). 1324 (s), 1158 (s), 998 (w), 749 (m), 715 (m). HR-MS (TOF-ES+) calc for $C_{11}H_{13}O_2S$ 209.0631, found 209.0631 ($M+H^+$).

This data is consistent with published work [W. Moran, et al., J. Org. Chem, 2016, 81, 6, 2543-2548.]

1-(*tert*-Butylsulfone)hex-1-yne, 10a:

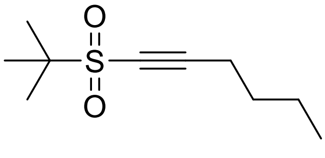

Chemical Formula: $C_{10}H_{18}O_2S$
Molecular Weight: 202.31

Compound **10a** was prepared as outlined in general synthesis procedure **2.1**, using 1-hexyne (5.2 mmol) and di-*tert*-butyl disulfide (5 mmol) affording the target compound in an 8% yield over the two steps as a colourless oil of poor purity (0.4 mmol, 81 mg).

^{1}H NMR (400 MHz, $CDCl_3$) δ 3.00 – 2.83 (m, 2H), 1.94 – 1.79 (m, 2H), 1.58 – 1.46 (m, 2H), 1.40 (s, 9H), 0.95 (t, *J* = 7.4 Hz, 3H). $^{13}C\{^{1}H\}$ NMR (101 MHz, $CDCl_3$) δ 109.9 (C), 77.3 (C), 59.0 (C), 45.2 (CH_2), 23.5 (3CH_3), 22.6 (CH_2), 22.2 (CH_2), 13.7 (CH_3). HR-MS (TOF-ES+) calc for $C_{10}H_{19}O_2S$ 203.1010, found 203.1009 ($M+H^+$).

((*tert*-Butylsulfone)ethynyl)benzene:

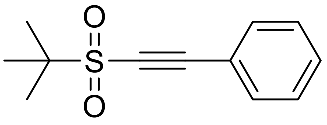

Chemical Formula: $C_{12}H_{14}O_2S$
Molecular Weight: 222.30

This compound could not be prepared under the general synthesis procedure **2.1**, using phenylacetylene (5.2 mmol) and di-*tert*-butyl disulfide (5 mmol).

Diethyl hex-1-yn-1-ylphosphonate, 11a:

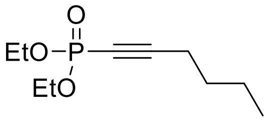

Chemical Formula: $C_{10}H_{19}O_3P$
Molecular Weight: 218.23

This compound (**11a**) was prepared using the general procedure outlined in section **2.2**, yielding the target phosphonate ester in a 68% yield as a pale-yellow oil. (3.4 mmol, 741.9 mg).

^{1}H NMR (400 MHz, $CDCl_3$) δ 4.32 – 4.00 (m, 4H), 2.35 (m, 2H), 1.79 – 1.50 (m, 2H), 1.46 – 1.18 (m, 8H), 0.90 (t, *J* = 9 Hz, 3H). ^{13}C{^{1}H} NMR (101 MHz, $CDCl_3$) δ 106.2 (d, *J* = 41 Hz, C), 73.3 (d, *J* = 212 Hz, C), 62.3 (d, *J* = 6.2 Hz, $2CH_2$), 29.4 (d, *J* = 1.9 Hz, CH_2), 22.0 (CH_2), 19.1 (d, *J* = 3.8 Hz, CH_2), 16.3 (d, *J* = 7.2 Hz, CH_3), 13.5 ($2CH_3$). ^{31}P NMR (162 MHz, $CDCl_3$) δ -5.6 (s). IR (neat) v/cm^{-1}: 2959 (m), 2932 (m), 2195 (vs), 1466 (w), 1424 (w), 1238 (vs), 1069 (s, b), 951 (vs), 764 (m). HR-MS (TOF-ES+) calc for $C_{10}H_{20}O_3P$ 219.1145, found 219.1145 (M+H$^+$).

This data is consistent with publish work [E. Karimi, N. Cockburn, W. Tam, J. Org. Chem. 2009, 74, 15, 5762, 5765].

Diethyl (phenylethynyl)phosphonate, 11b:

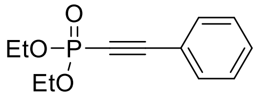

Chemical Formula: $C_{12}H_{15}O_3P$
Molecular Weight: 238.22

Compound **11b** was prepared using the general procedure outlined in section 2.2 yielding the target phosphonate ester in a 71% yield as a colourless oil. (3.5 mmol, 846 mg).

^{1}H NMR (400 MHz, $CDCl_3$) δ 7.55 (dd, *J* = 8 Hz, 2H), 7.44 (t, *J* = 7.6 Hz, 1H), 7.36 (t, *J* = 7.6 Hz, 2H), 4.22 (m, 4H), 1.40 (td, *J* = 7.1, 0.7 Hz, 6H). ^{13}C{^{1}H} NMR (101 MHz, $CDCl_3$) δ 132.7 (d, *J* = 2.7 Hz, 2CH), 130.7 (CH), 128.6 (2CH), 119.6 (d, *J* = 5.4 Hz, C), 99.1 (d, *J* = 53.4 Hz, C), 78.4 (d, *J* = 299.7 Hz, C), 63.3 (d, *J* = 5.5 Hz, $2CH_3$),16.2 (d, *J* = 7.1 Hz, $2CH_3$). ^{31}P NMR (162 MHz, $CDCl_3$) δ -5.4. IR (neat) v/cm^{-1}: 2984 (w), 2937 (w), 2184 (s), 1509 (w), 1260 (s), 1015 (vs), 973 (s), 853 (s), 689 (m), 541 (m). HR-MS (TOF-ES+) calc for $C_{12}H_{16}O_3P$ 239.0787, found 239.0787(M+H$^+$).

This data is consistent with publish work [E. Karimi, N. Cockburn, W. Tam, J. Org. Chem. 2009, 74, 15, 5762, 5765].

6. Copies of NMR Spectra

3a: 1-Phenylhept-2-yn-1-one

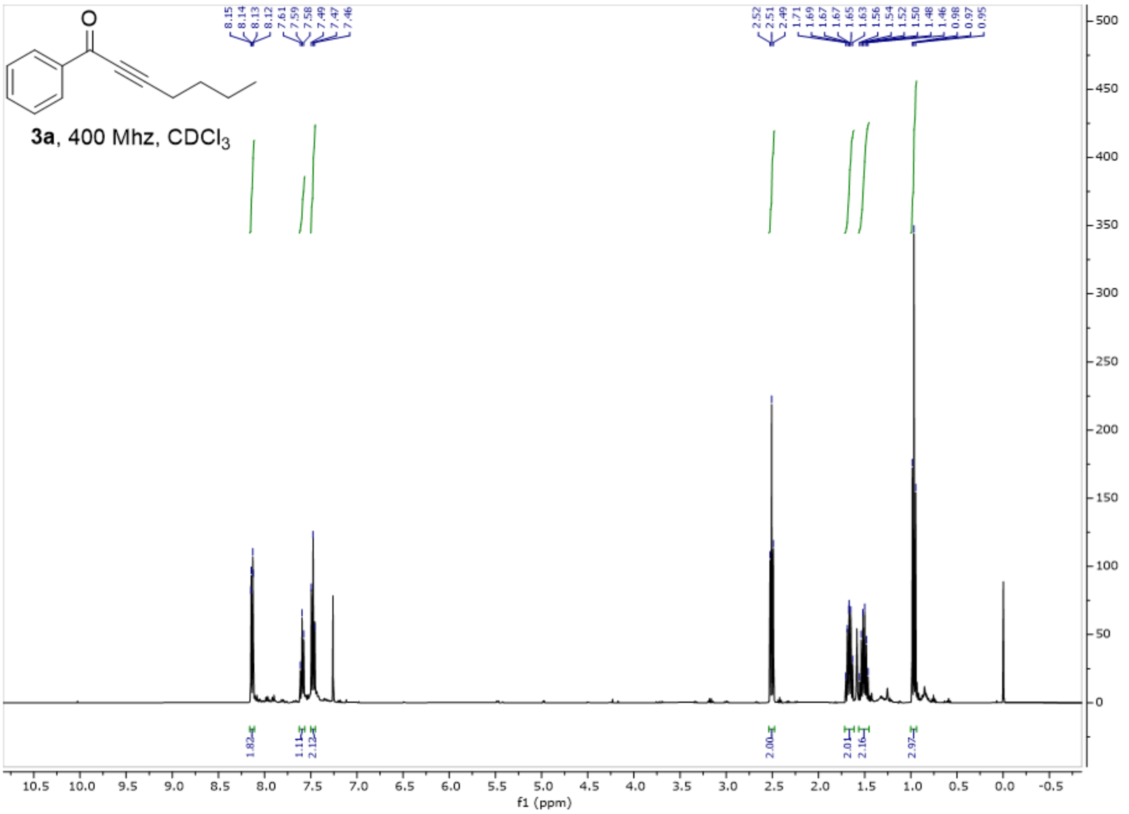

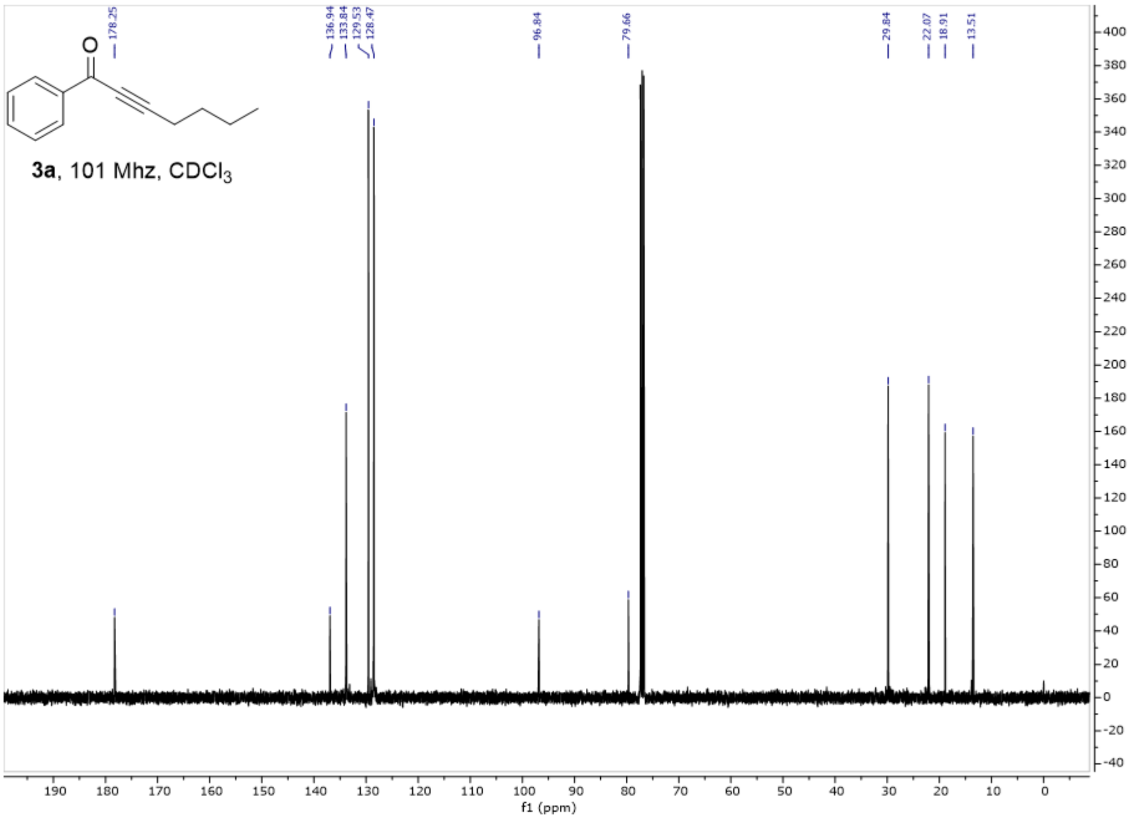

3b: 1-(4-Methoxyphenyl)hept-2-yn-1-one

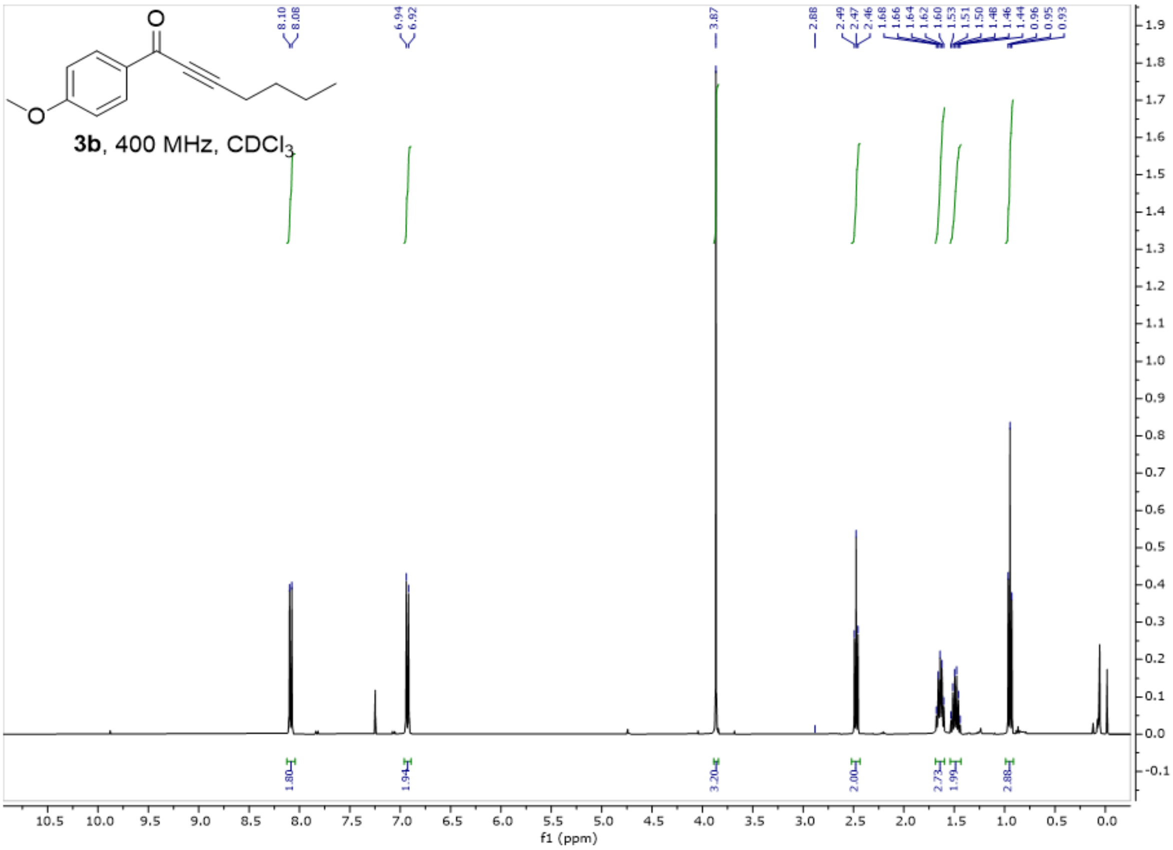

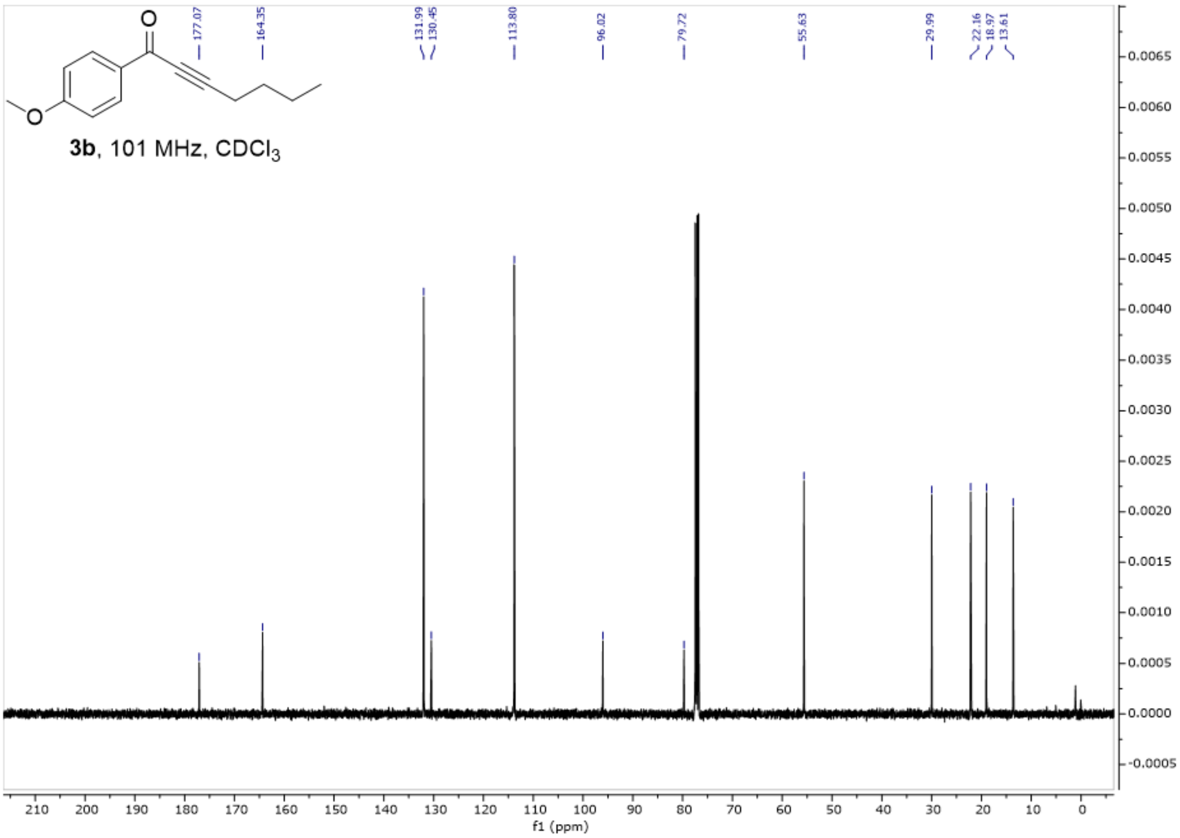

3c: 1-(4-(Trifluoromethyl)phenyl)Hept-2-yn-1-one

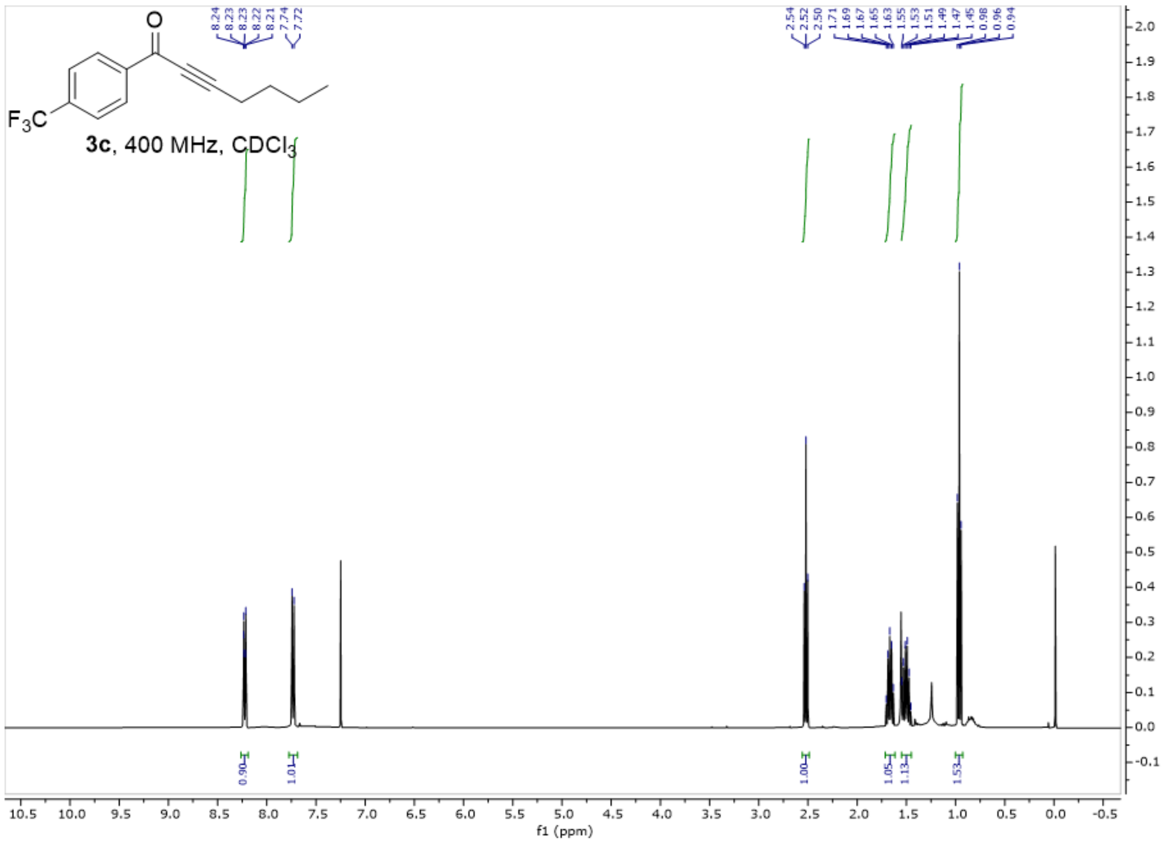

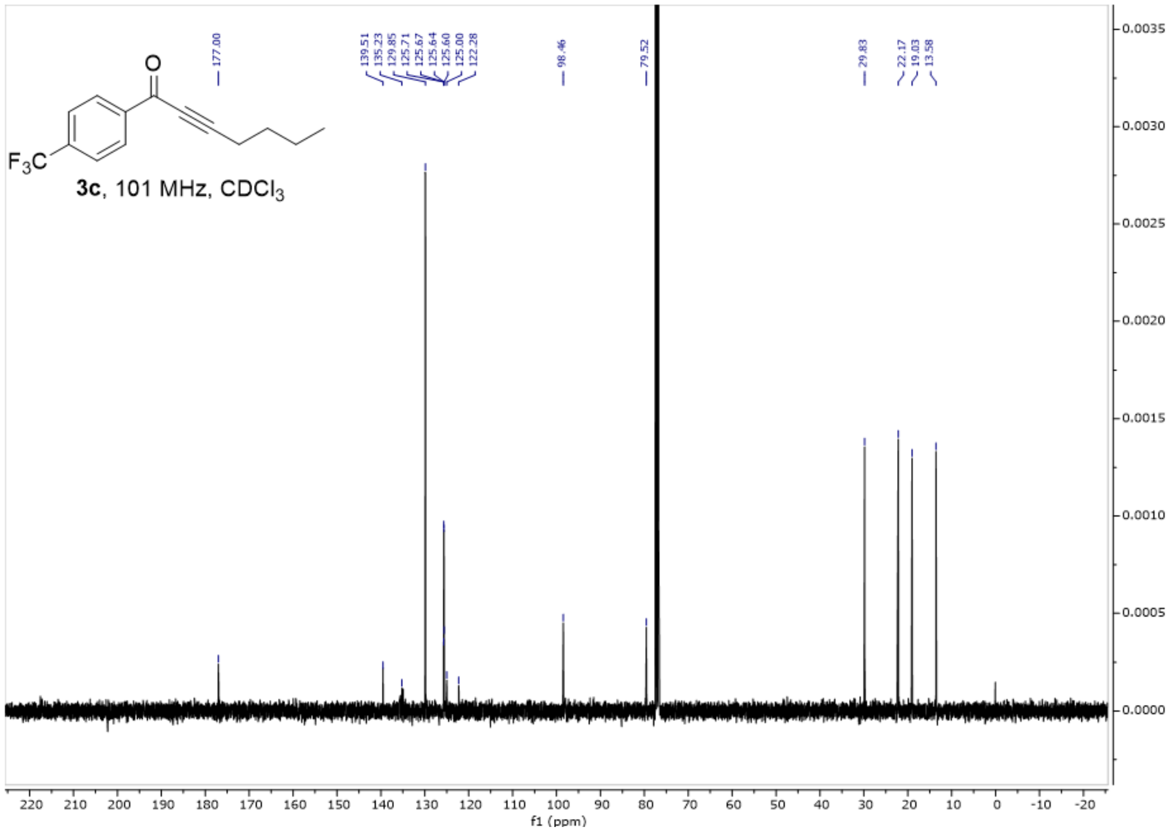

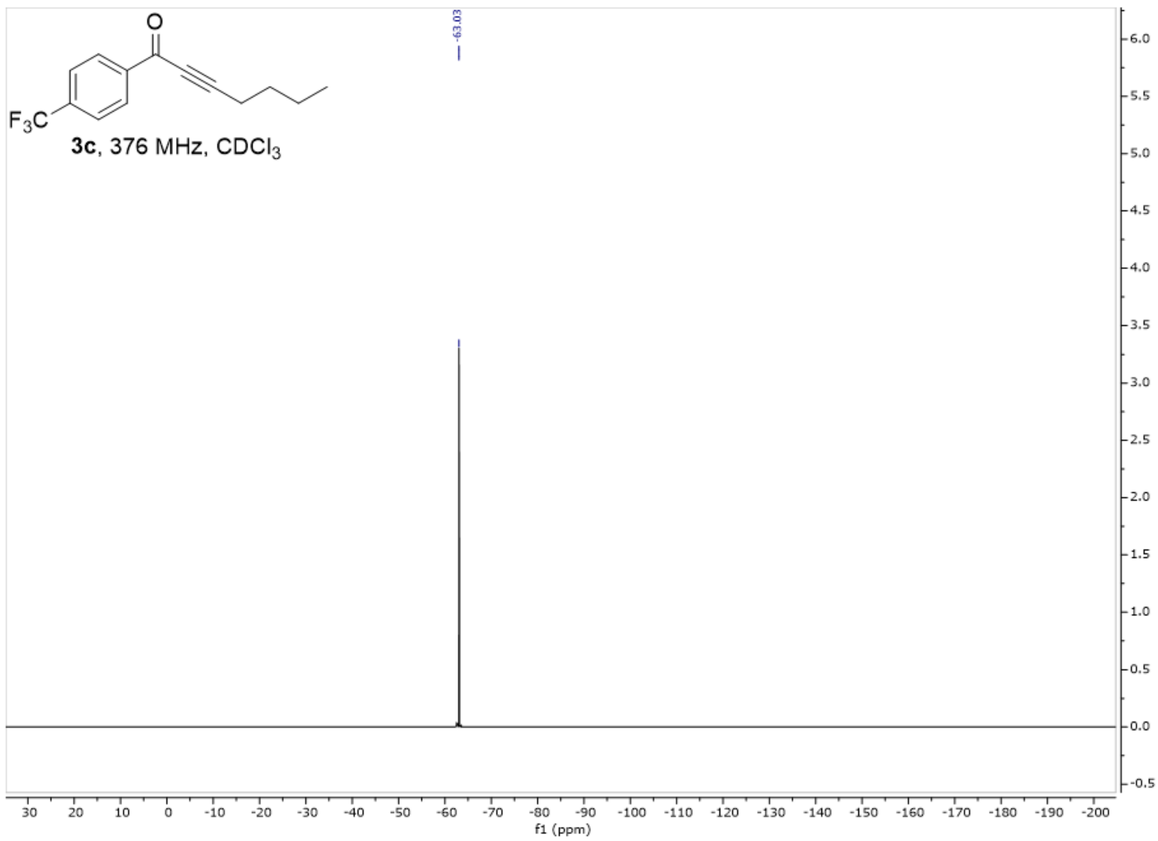
F3C
3c, 376 MHz, CDCl3
-63.03
f1 (ppm)

3d: 1-(4-Chlorophenyl)hept-2-yn-1-one

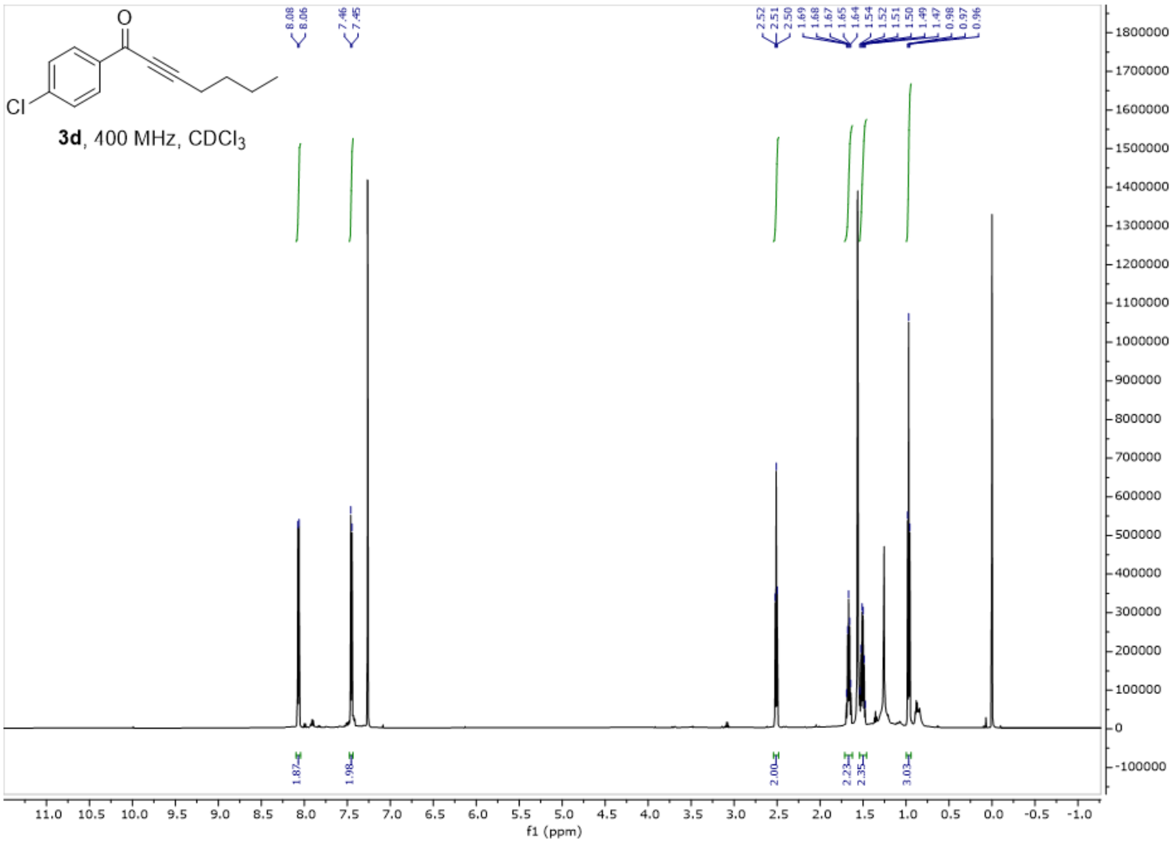

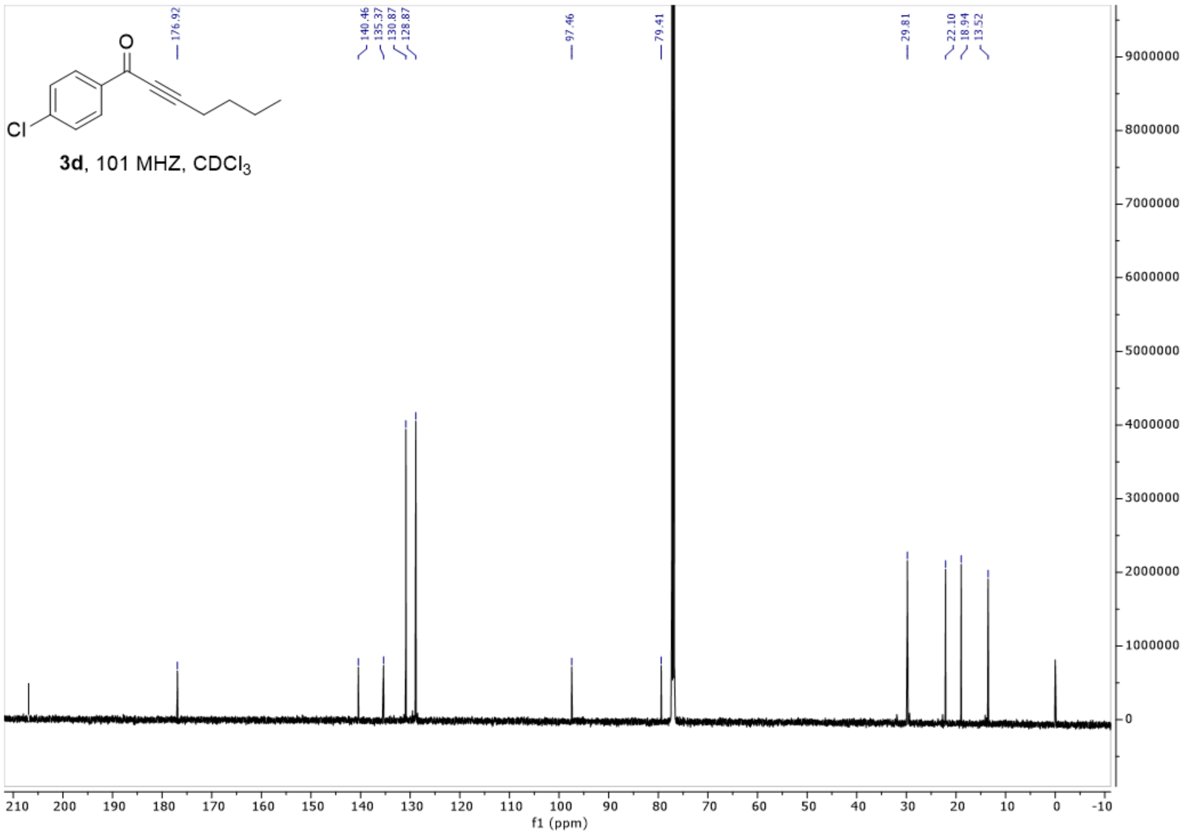

3e: 1-(4-Bromophenyl)hept-2-yn-1-one

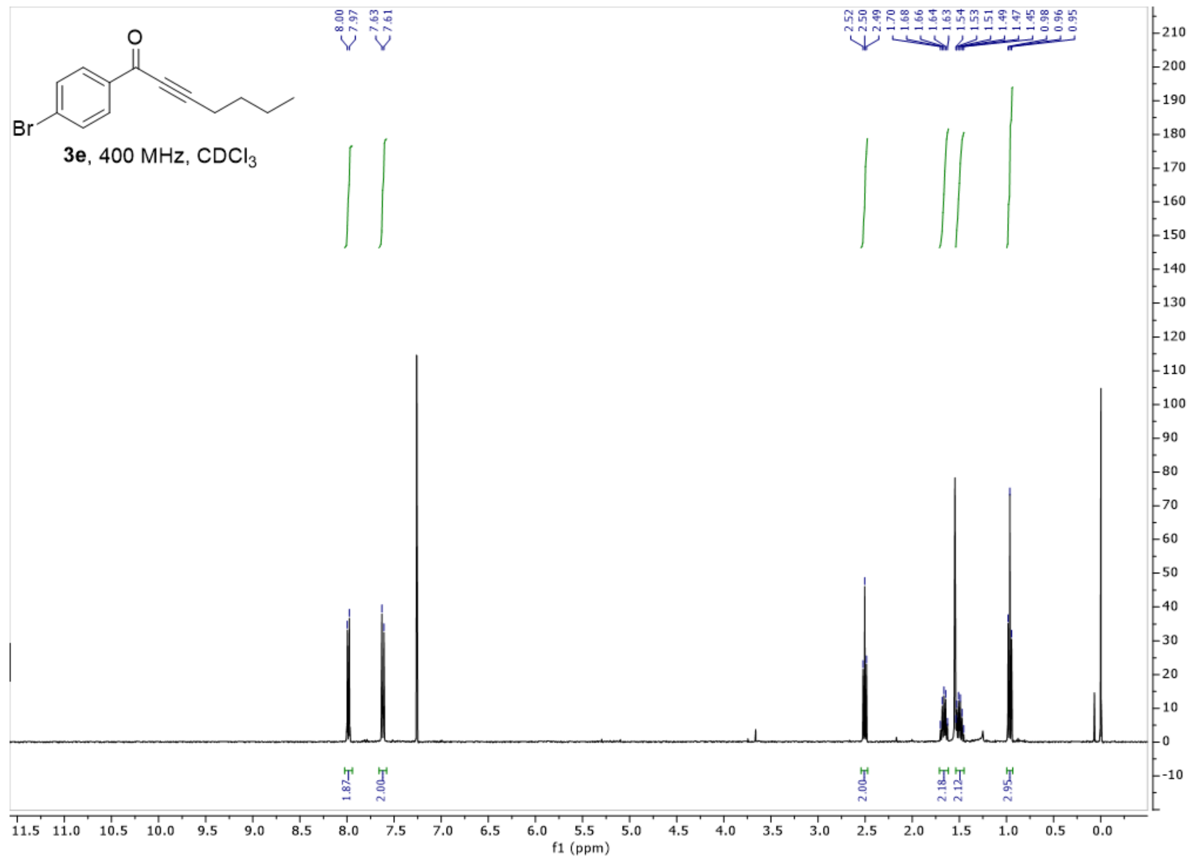

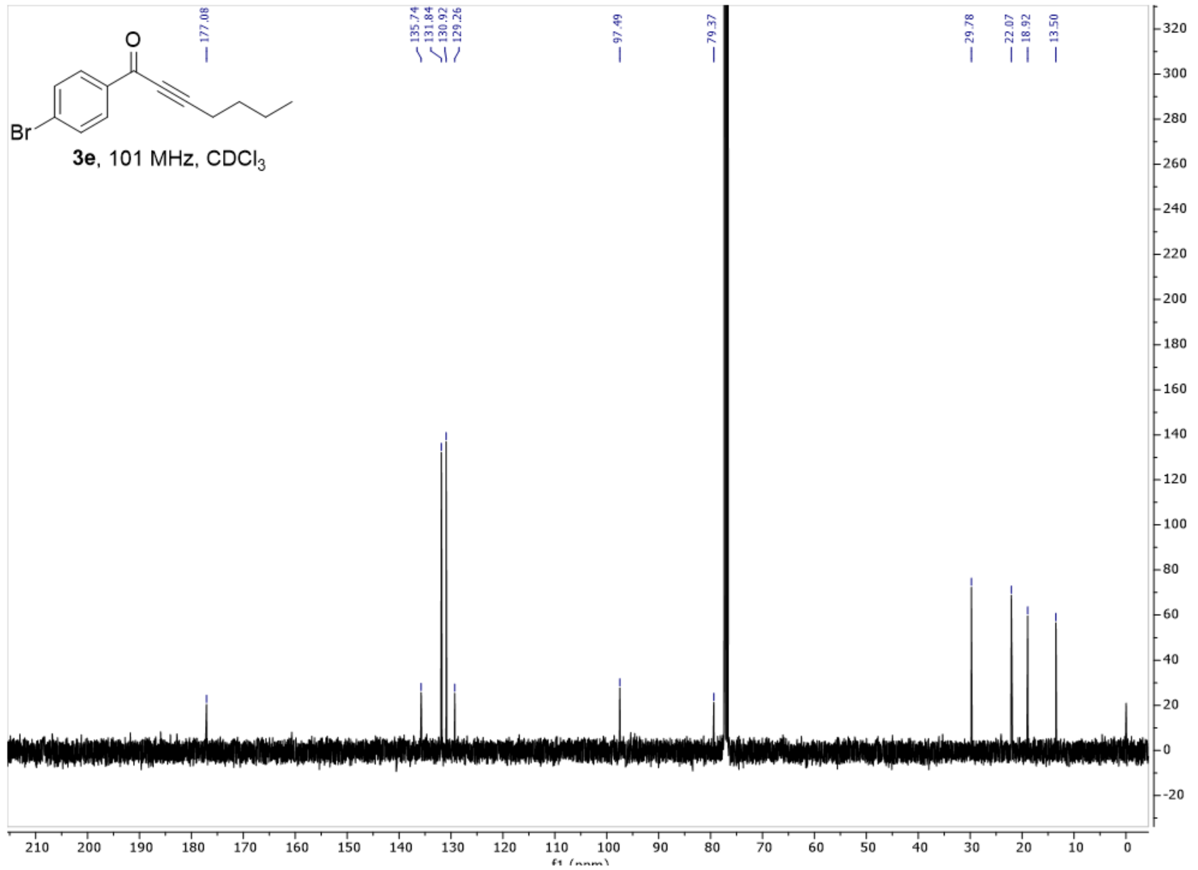

3f: 1-(4-(4,4,5,5-Tetramethyl-1,3,2-dioxaborolan-2-yl)phenyl)hept-2-yn-1-one

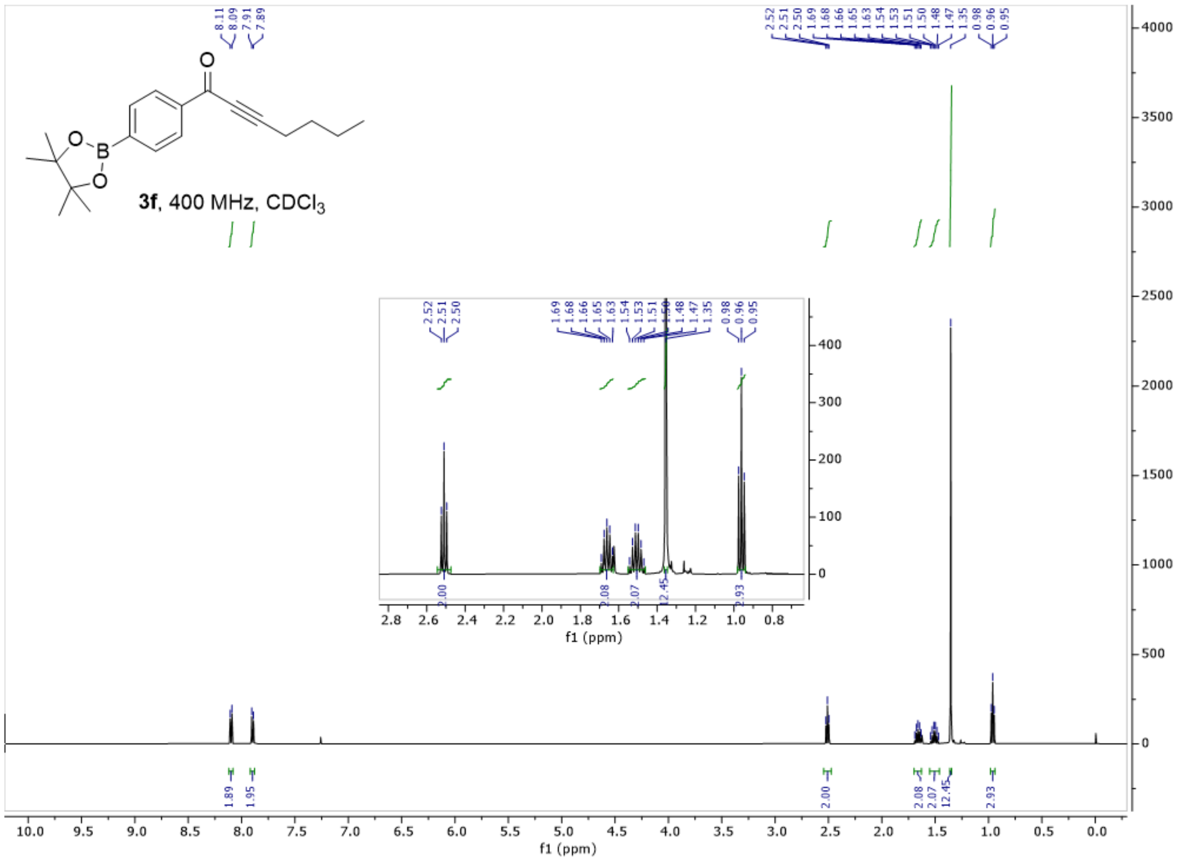

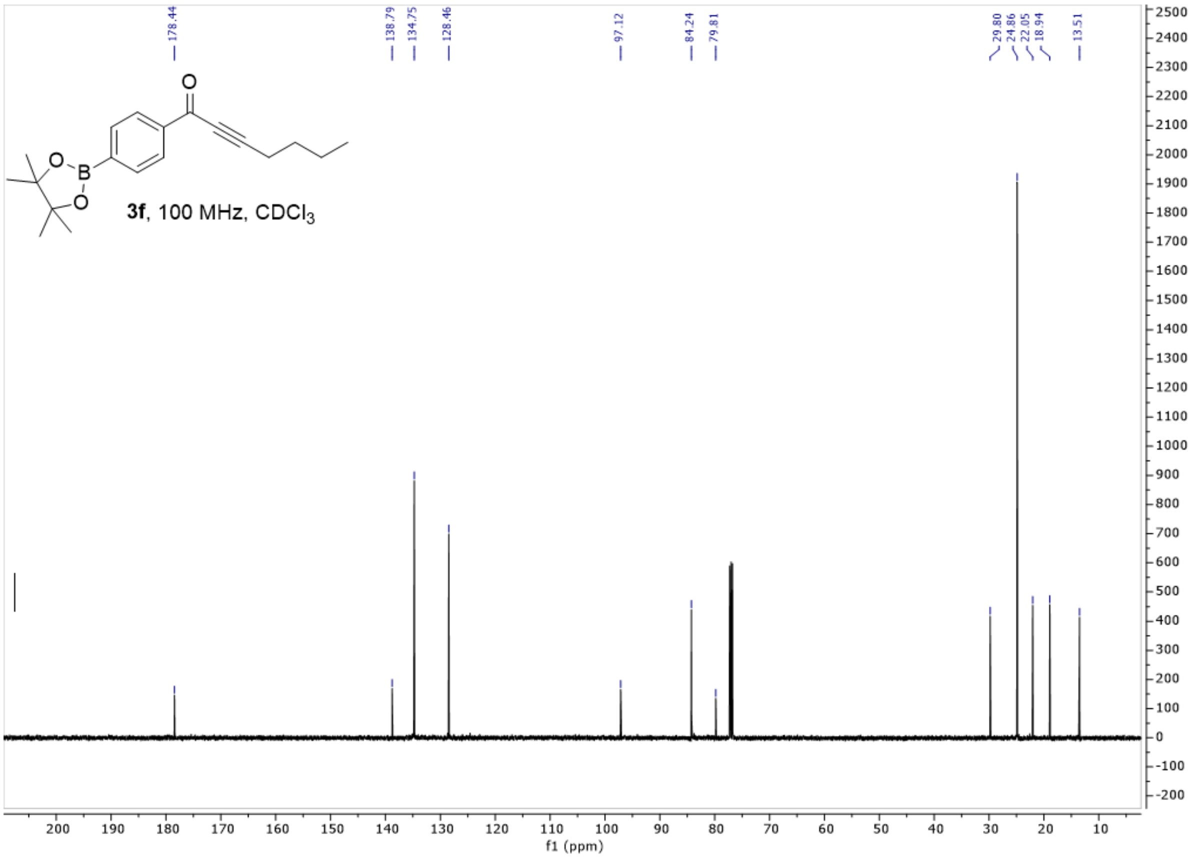

3g: 4-(Hept-2-ynoyl)phenyl acetate

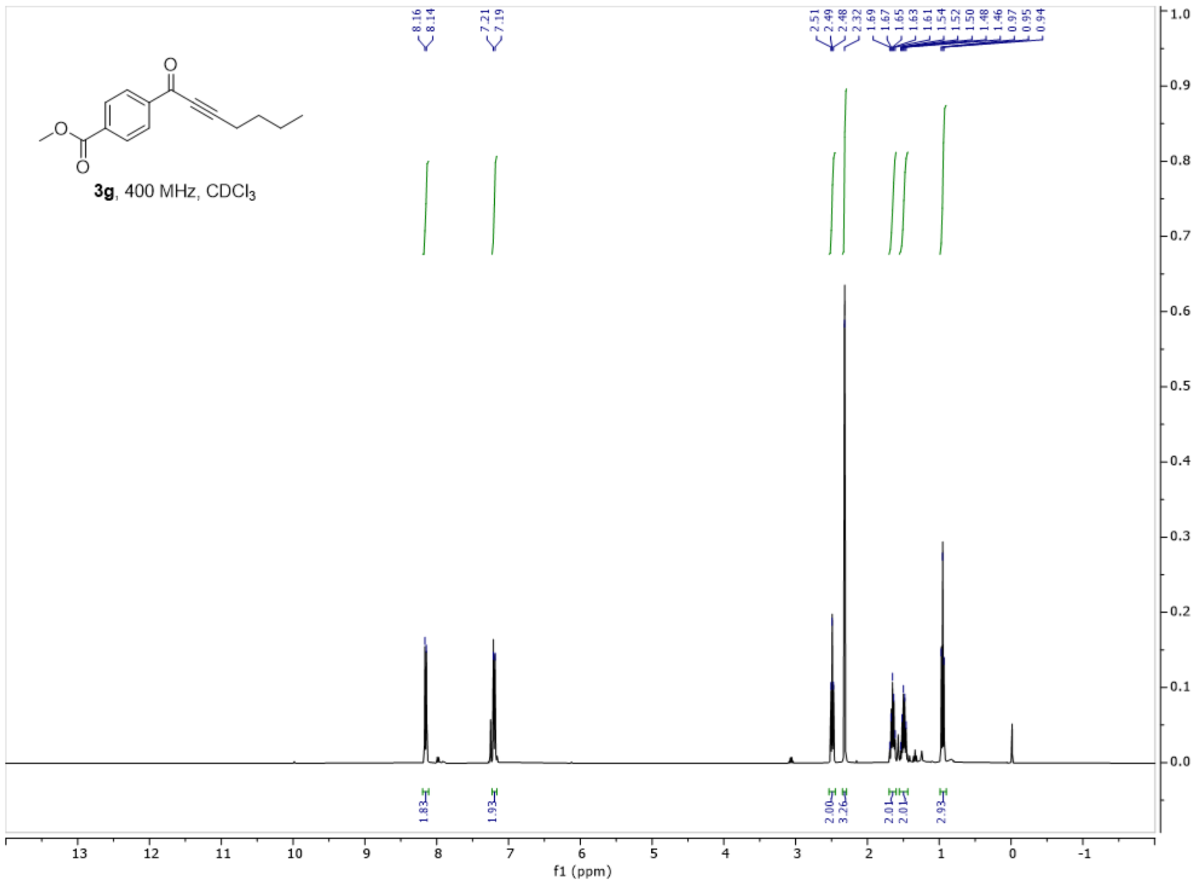

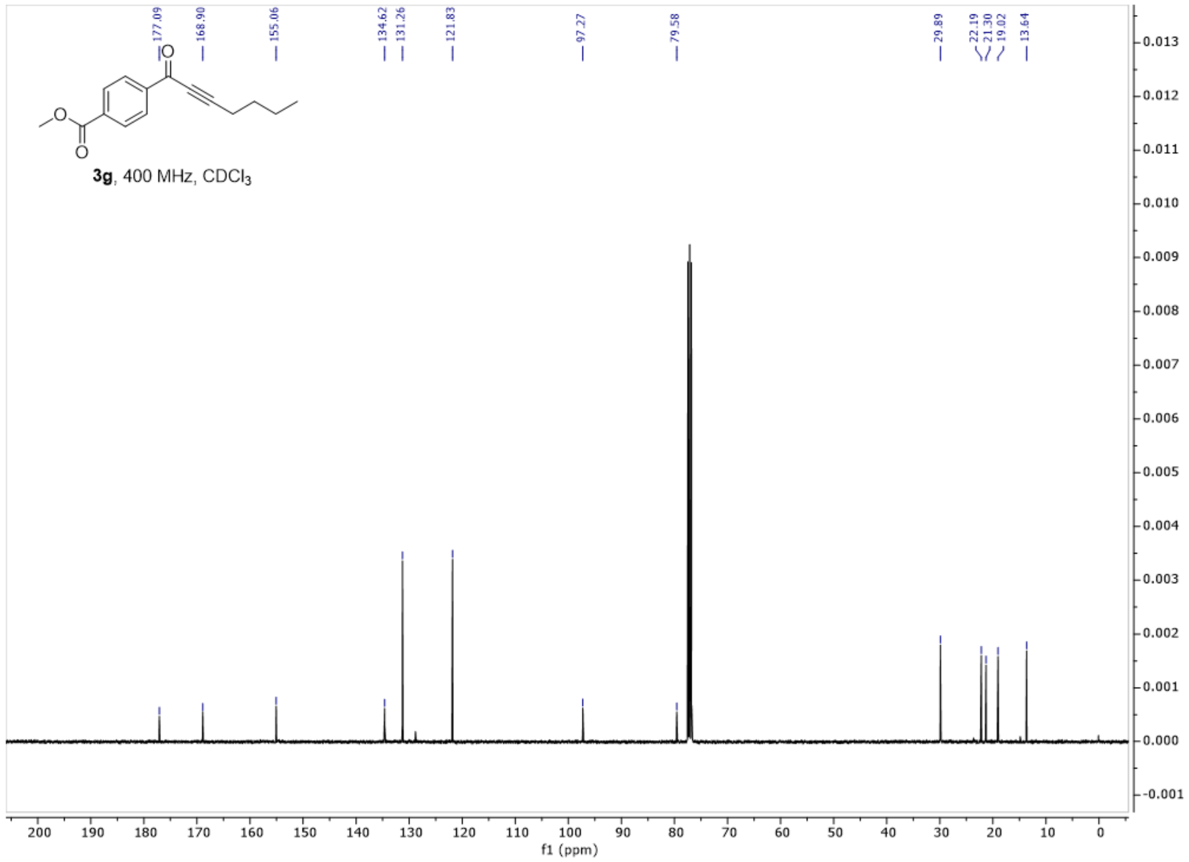

3h: 1-(Thiophen-2-yl)hept-2-yn-1-one

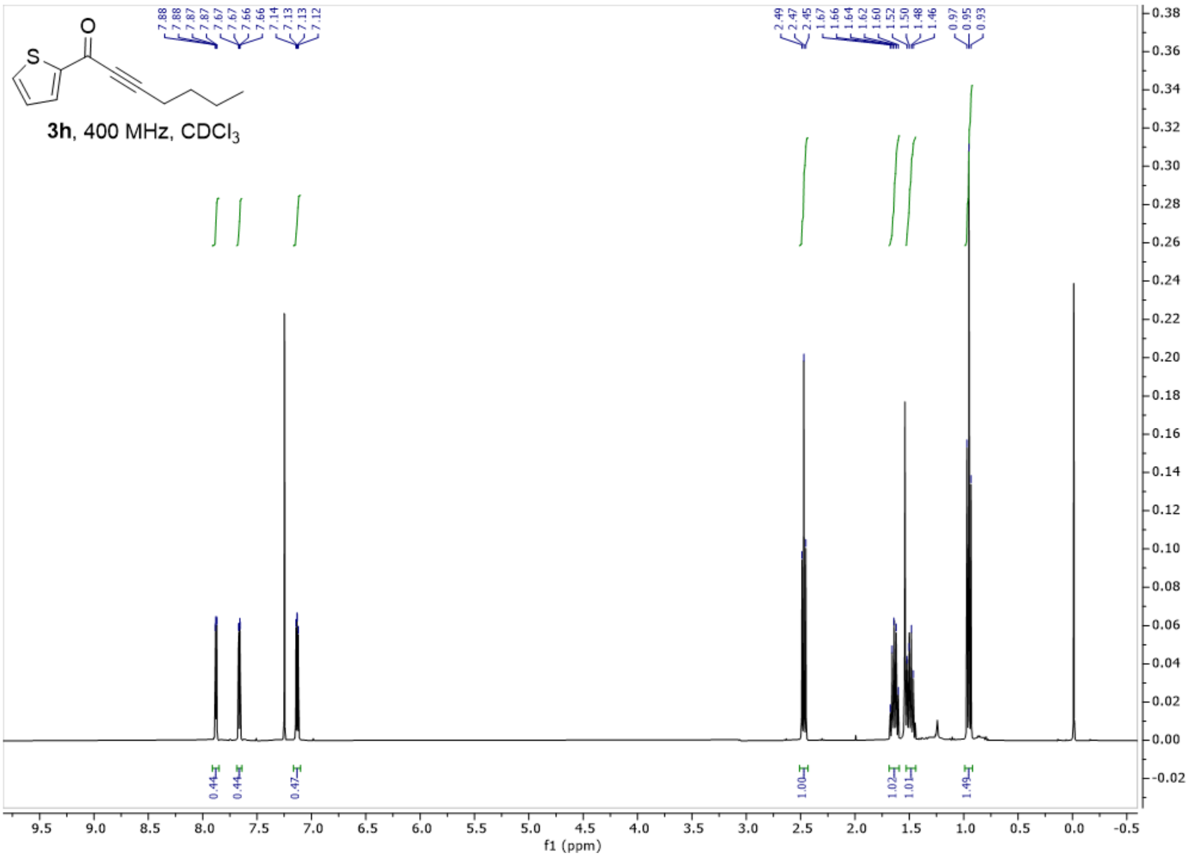

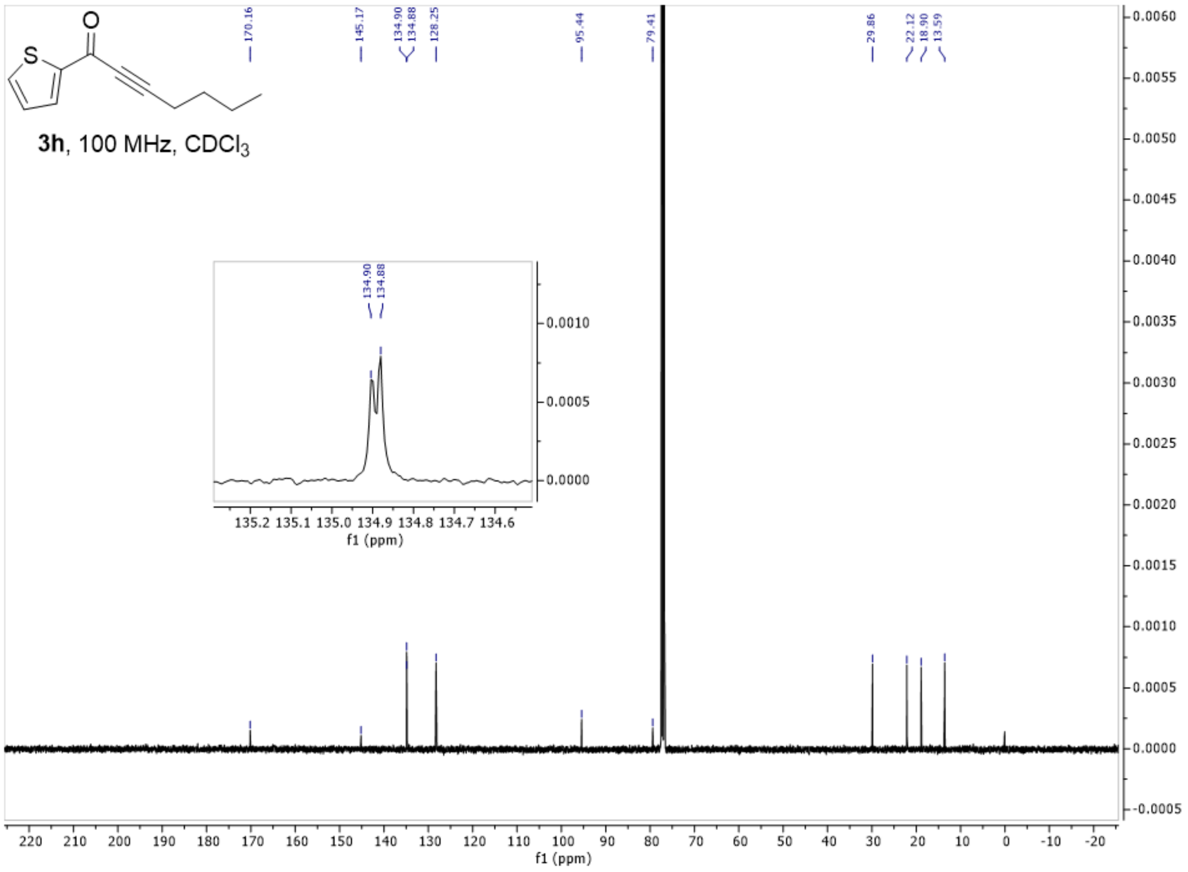

3i: 1-(1-Methyl-1H-indol-2-yl)hept-2-yn-1-one

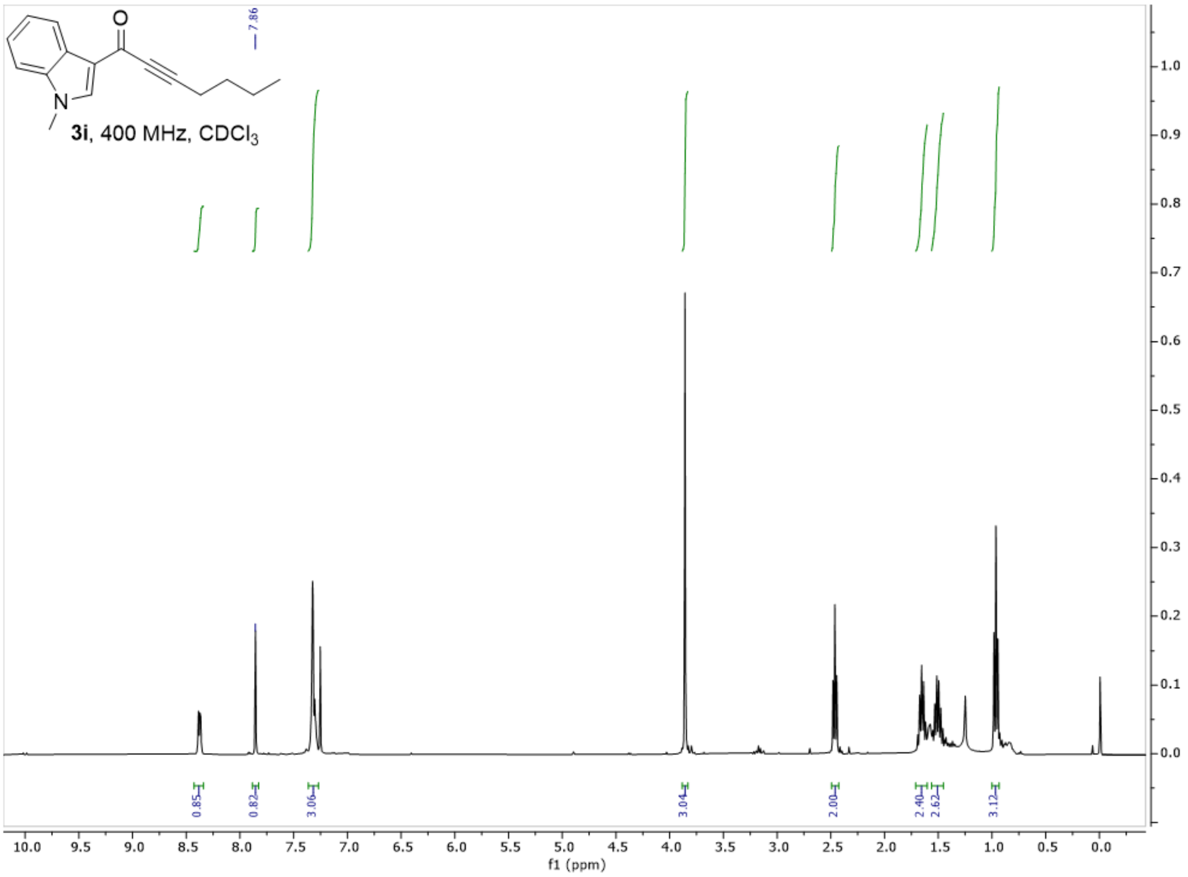

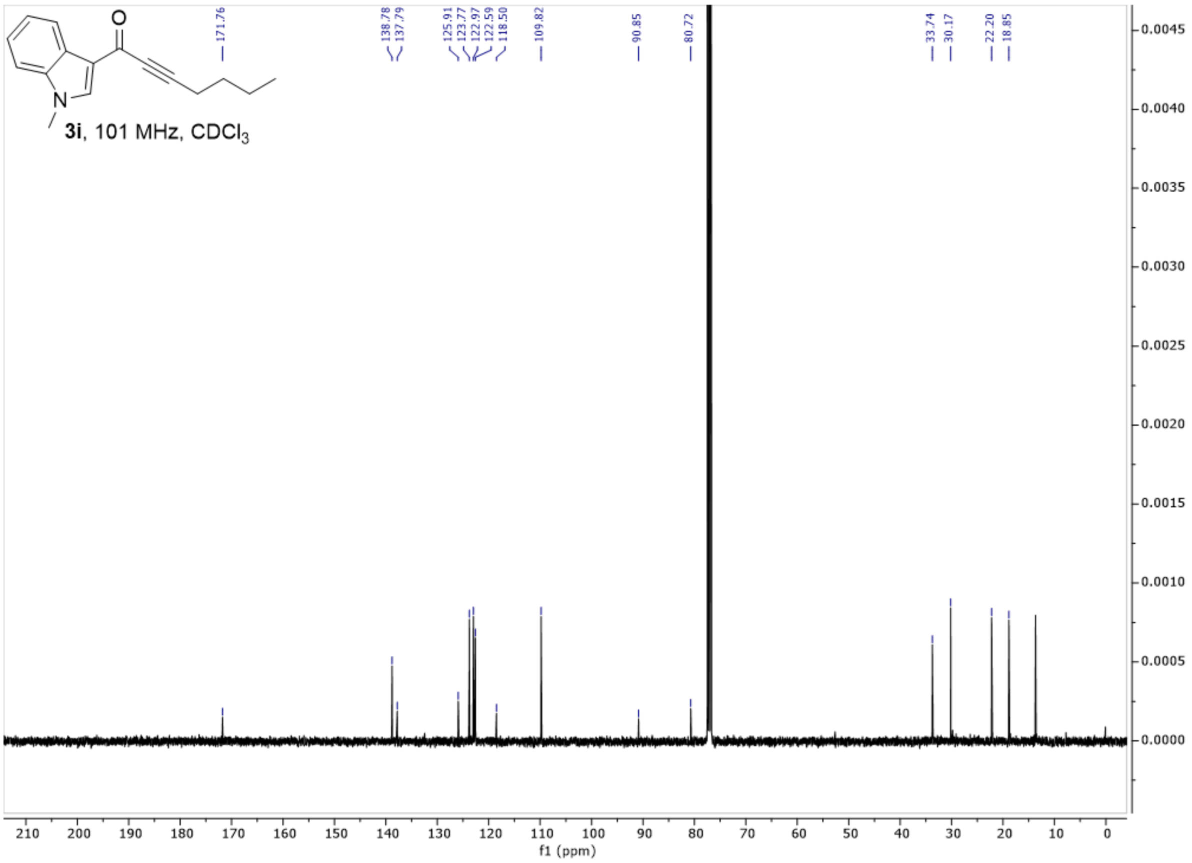

3j: 1-Cyclohexylhept-2-yn-1-one

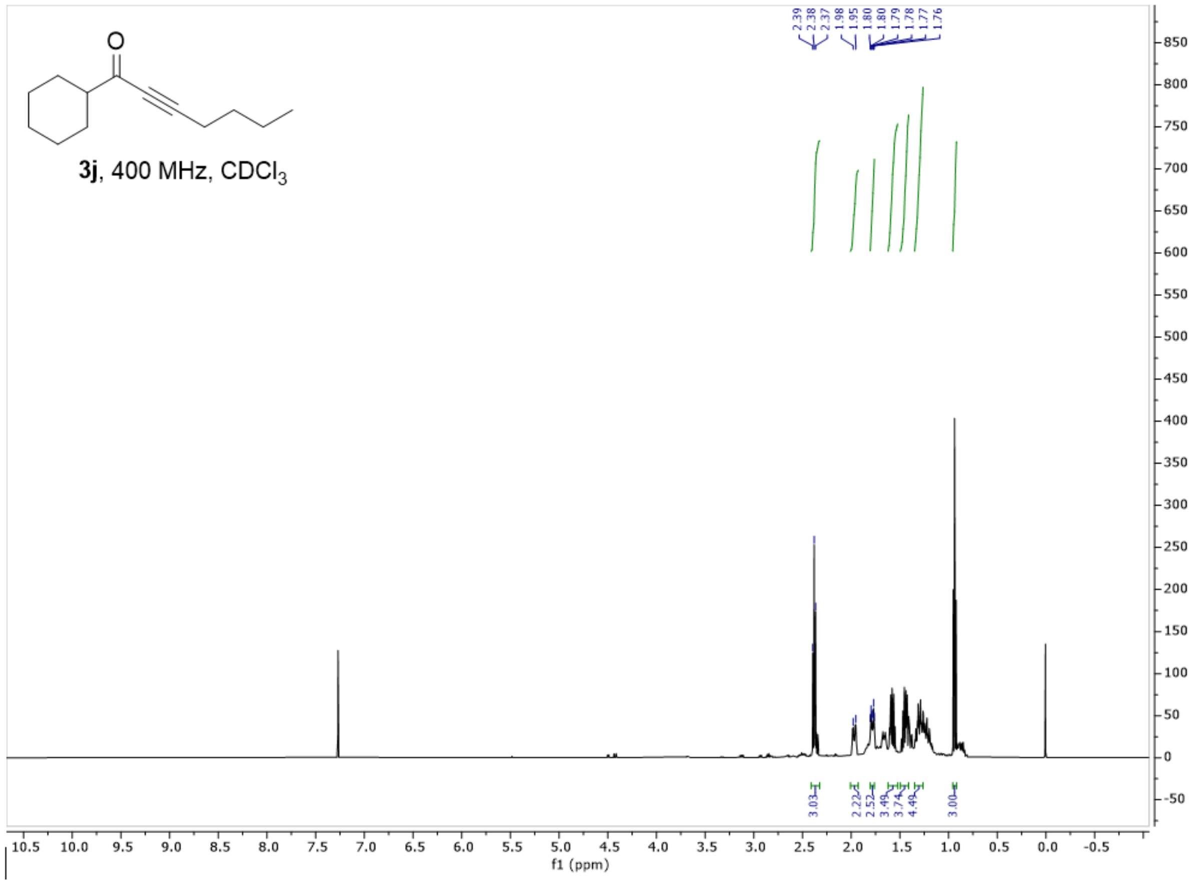

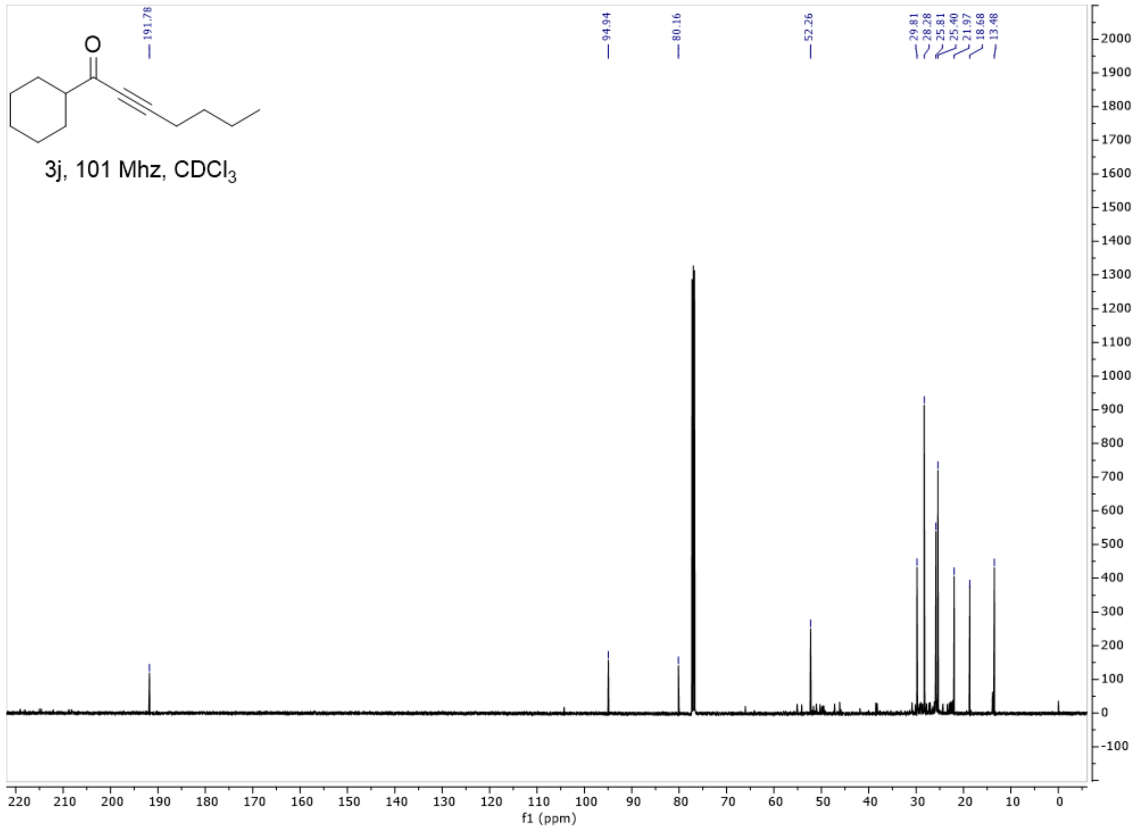

4a: 1-(4-Methoxyphenyl)-3-phenylprop-2-yn-1-one

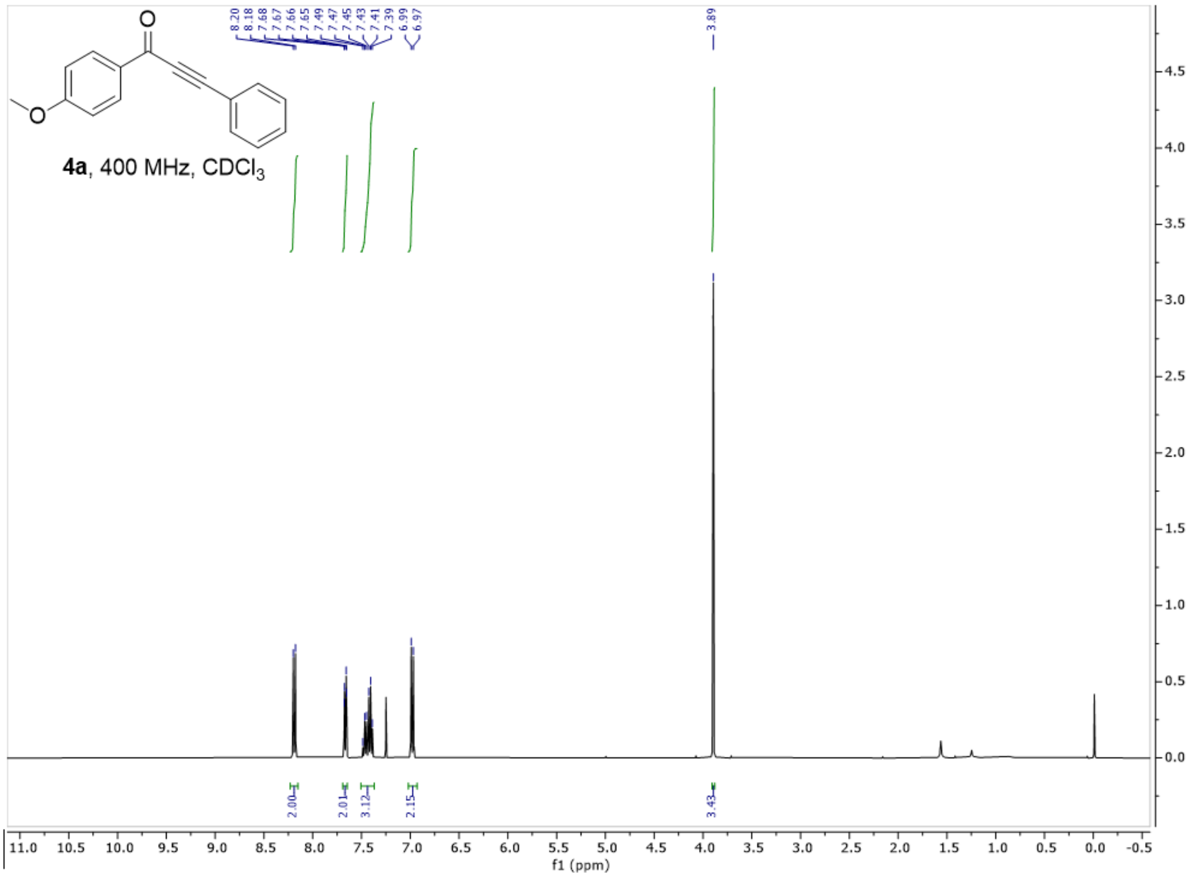

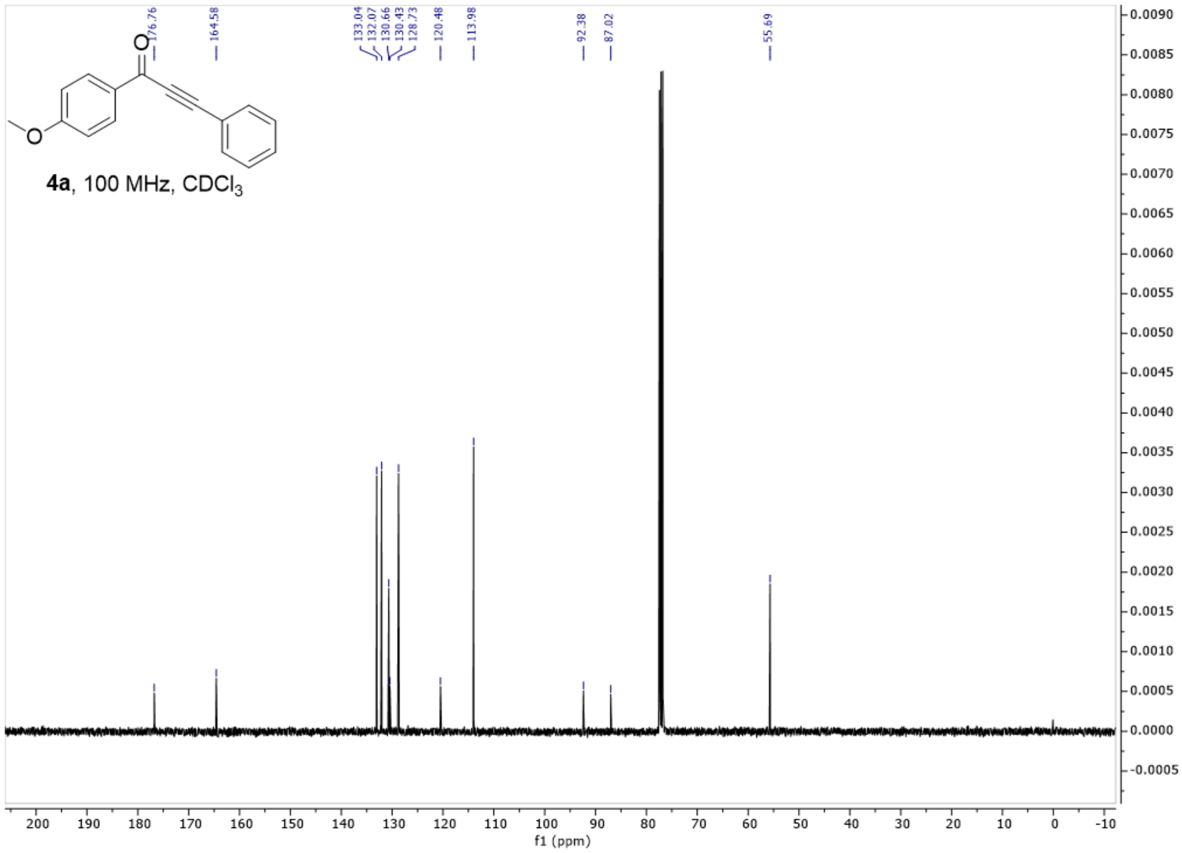

4b: 1,3-Diphenylprop-2-yn-1-one

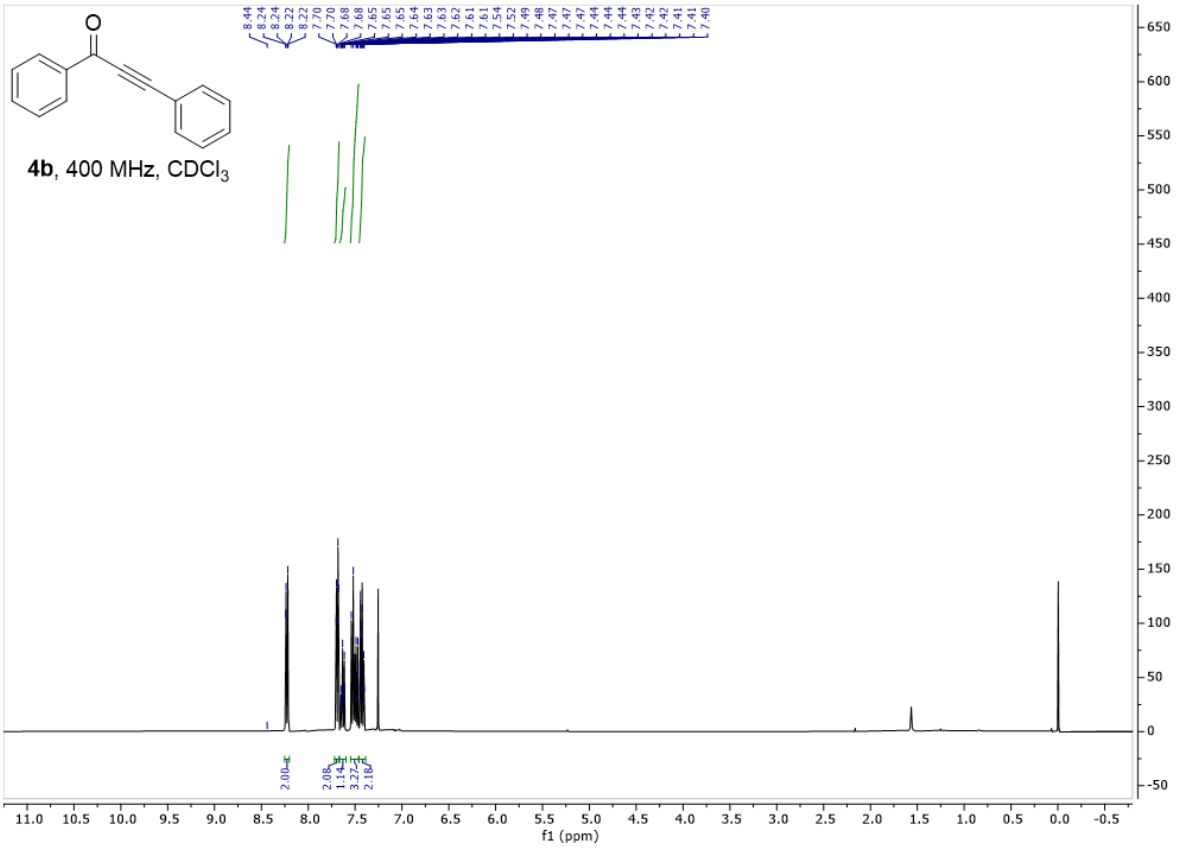

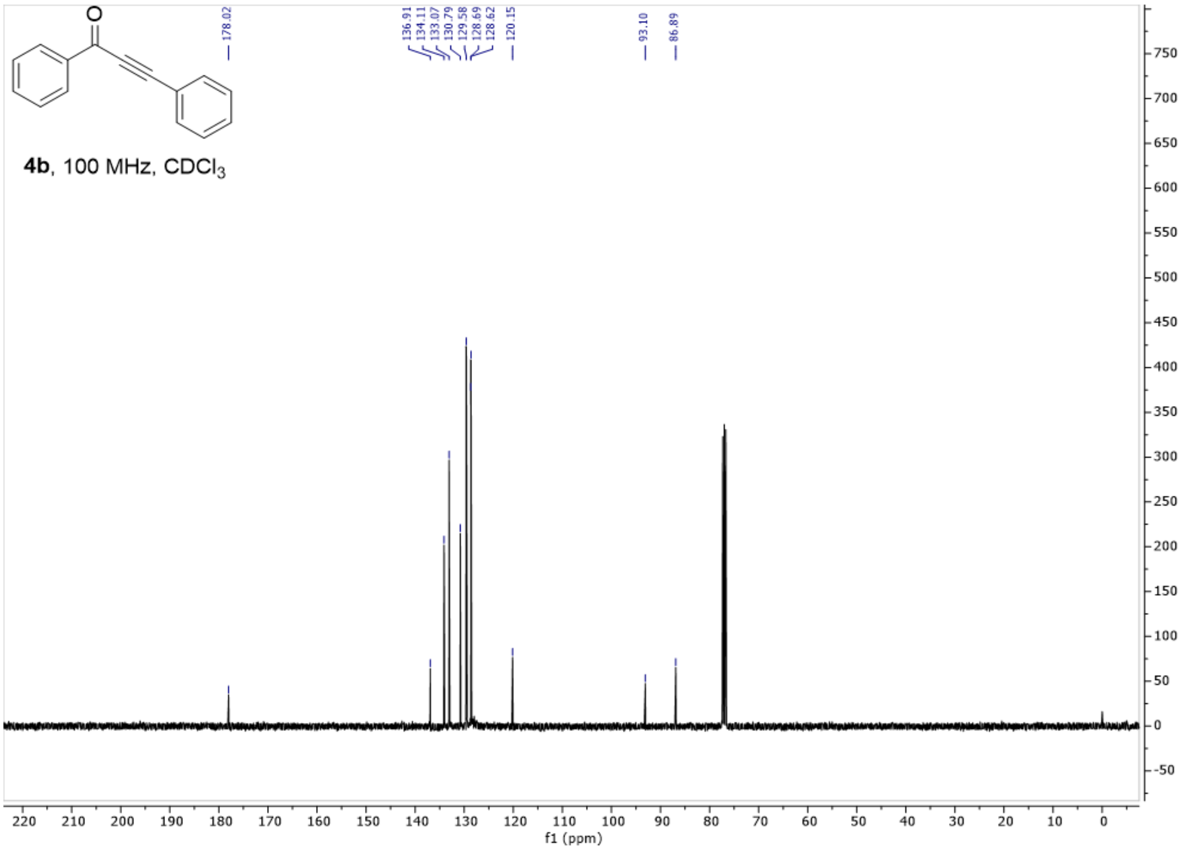

4c: 3-Phenyl-1-(4-(trifluoromethyl)phenyl)prop-2-yn-1-one

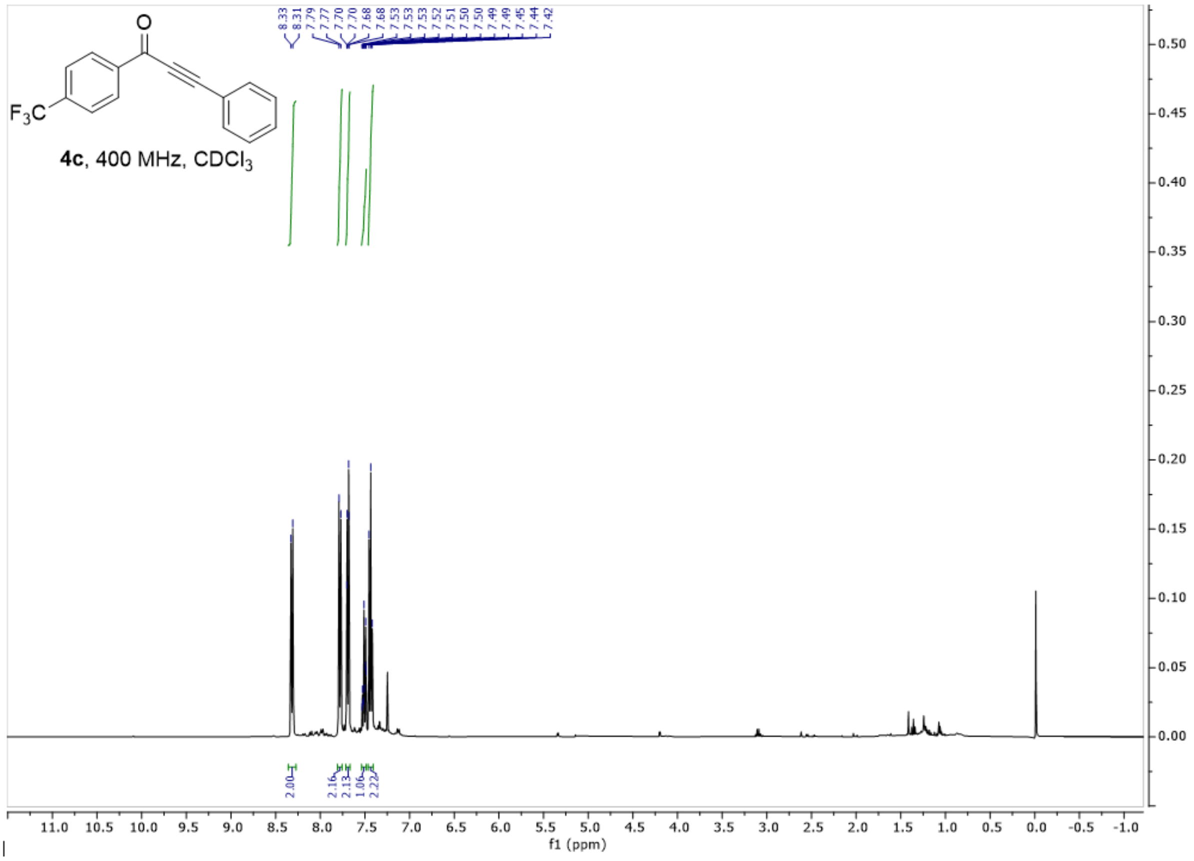

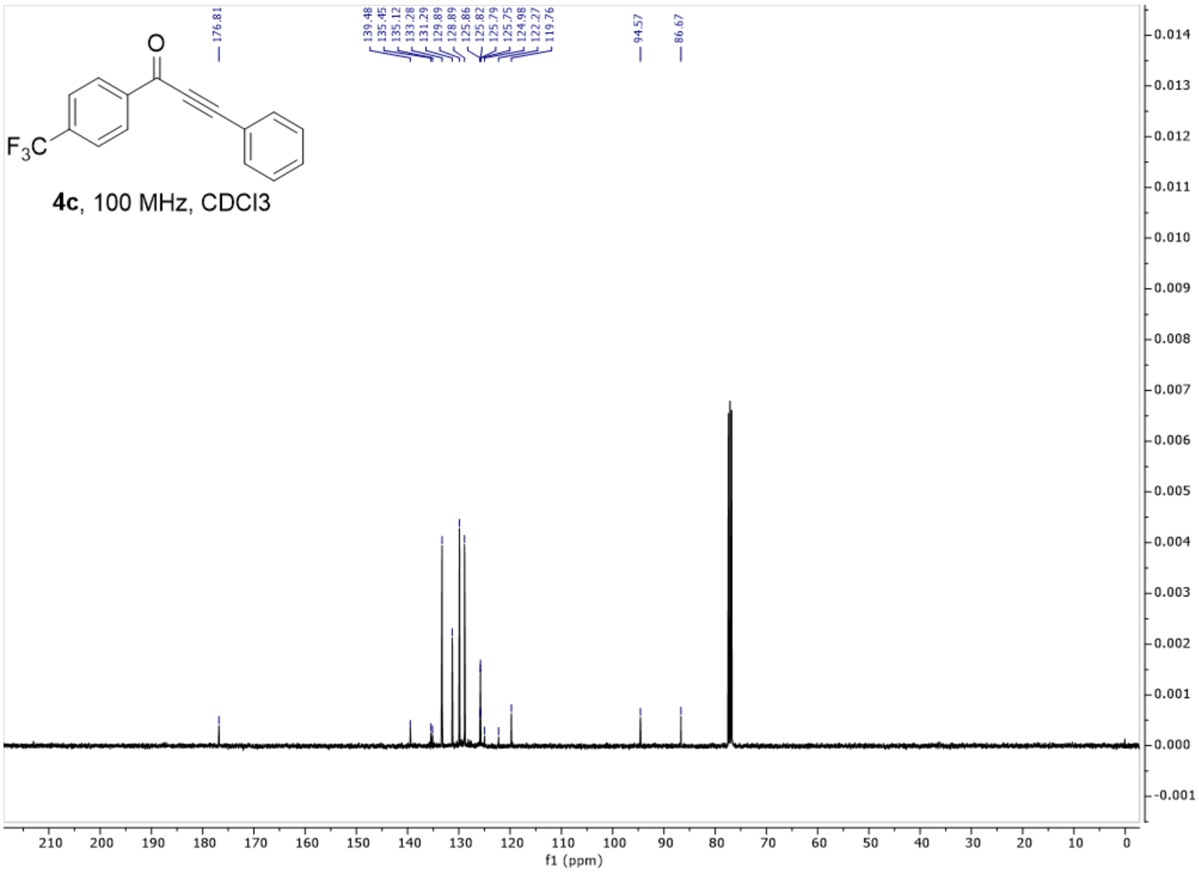

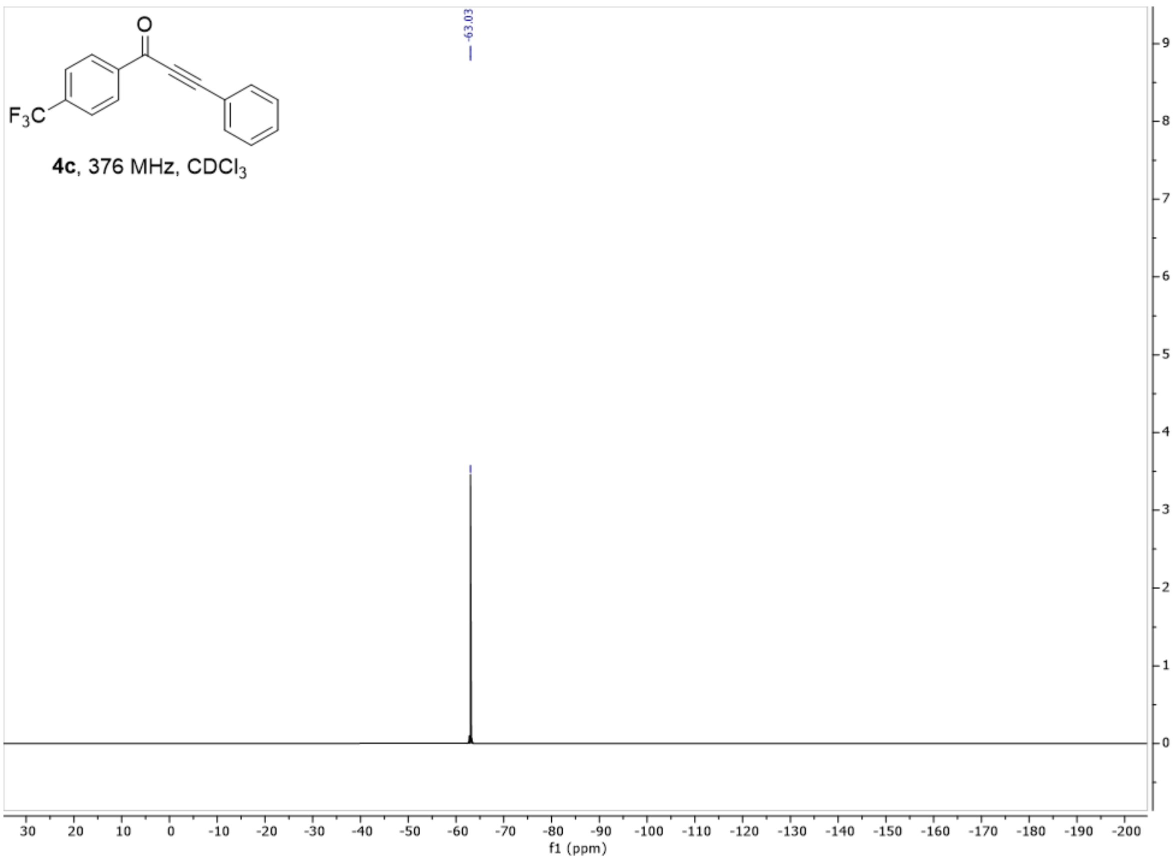

4c, 376 MHz, CDCl3
-63.03
f1 (ppm)

4d: 3-Cyclopropyl-1-phenylprop-2-yn-1-one

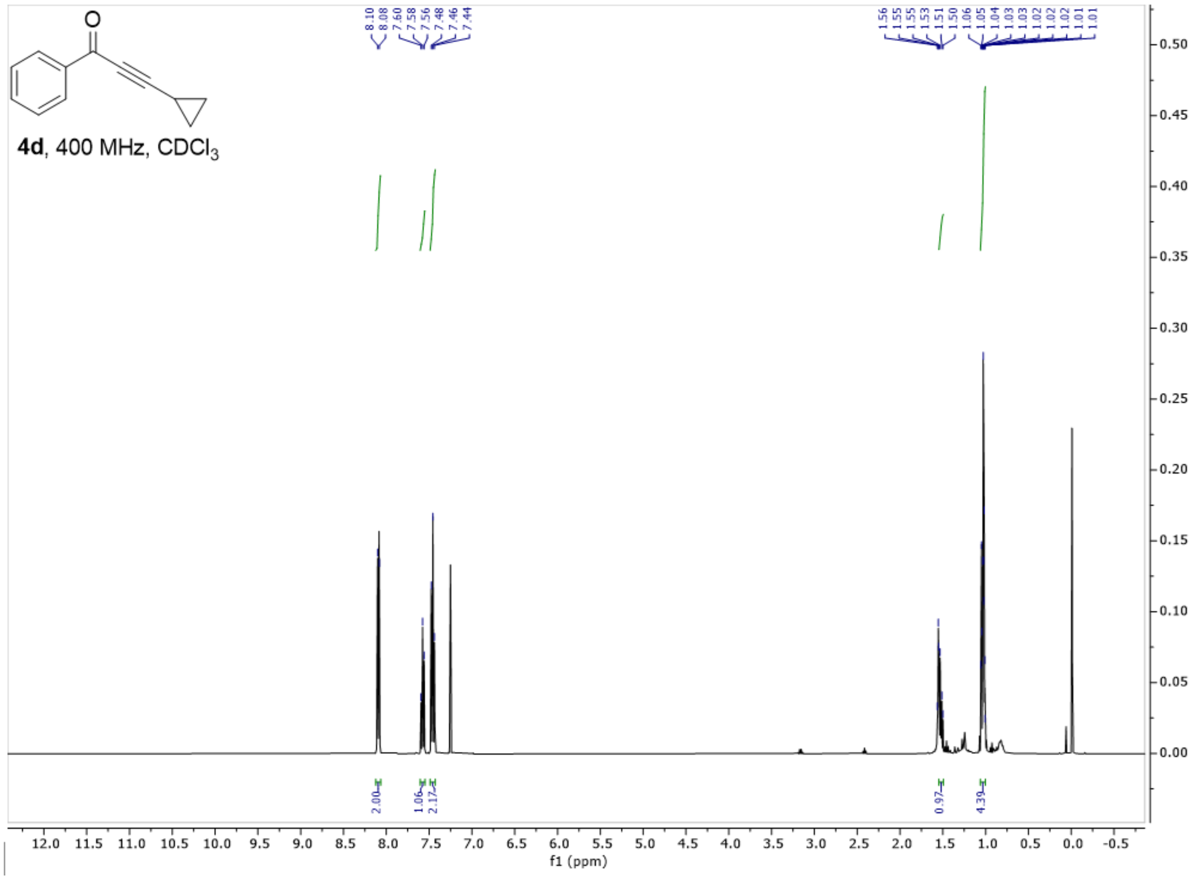

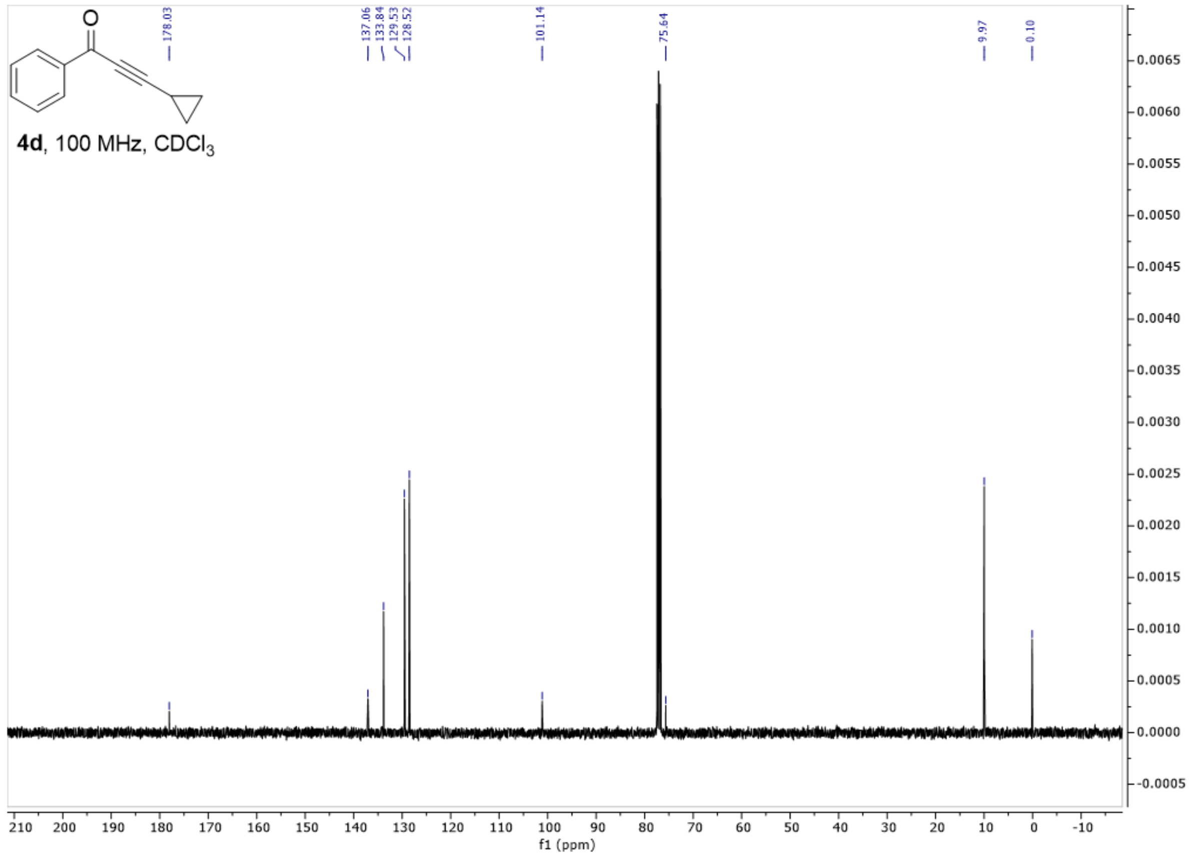

4e: 1-phenylnon-1-yn-3-one

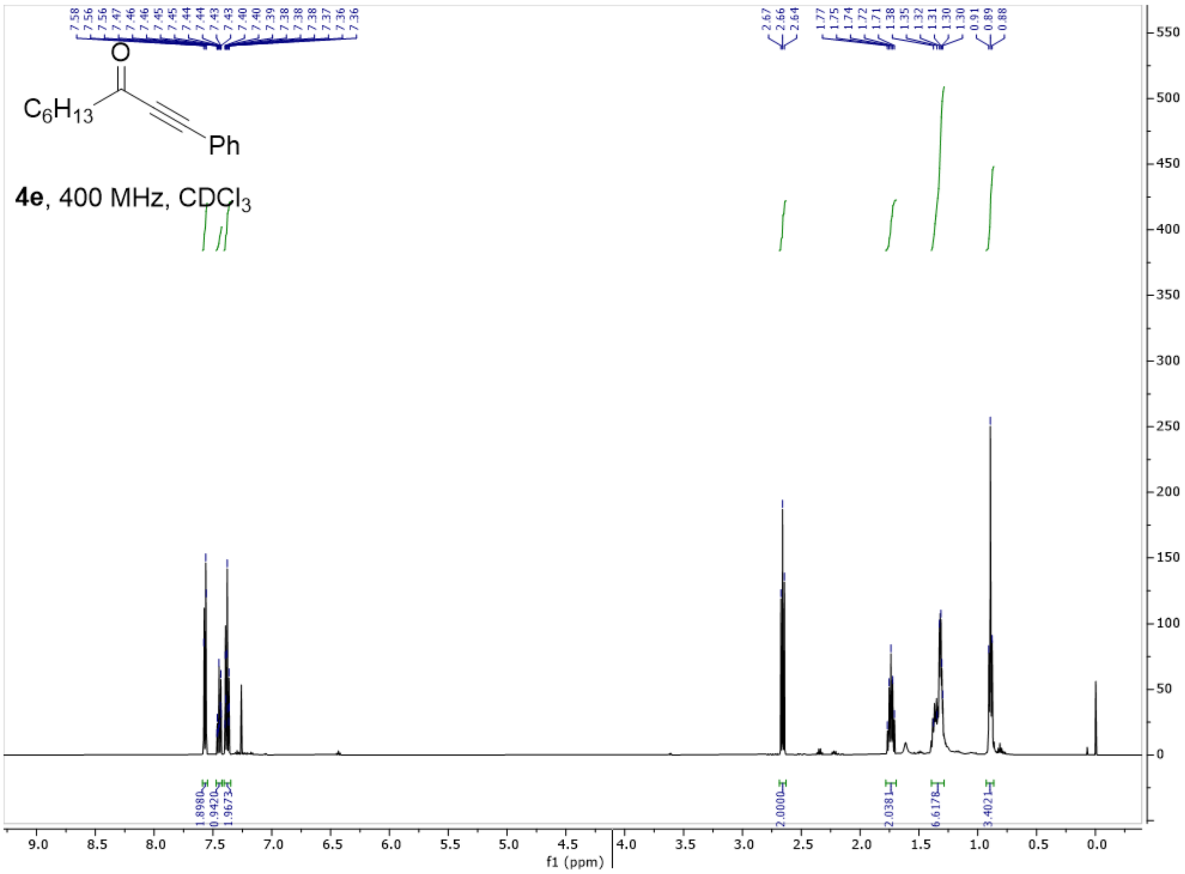

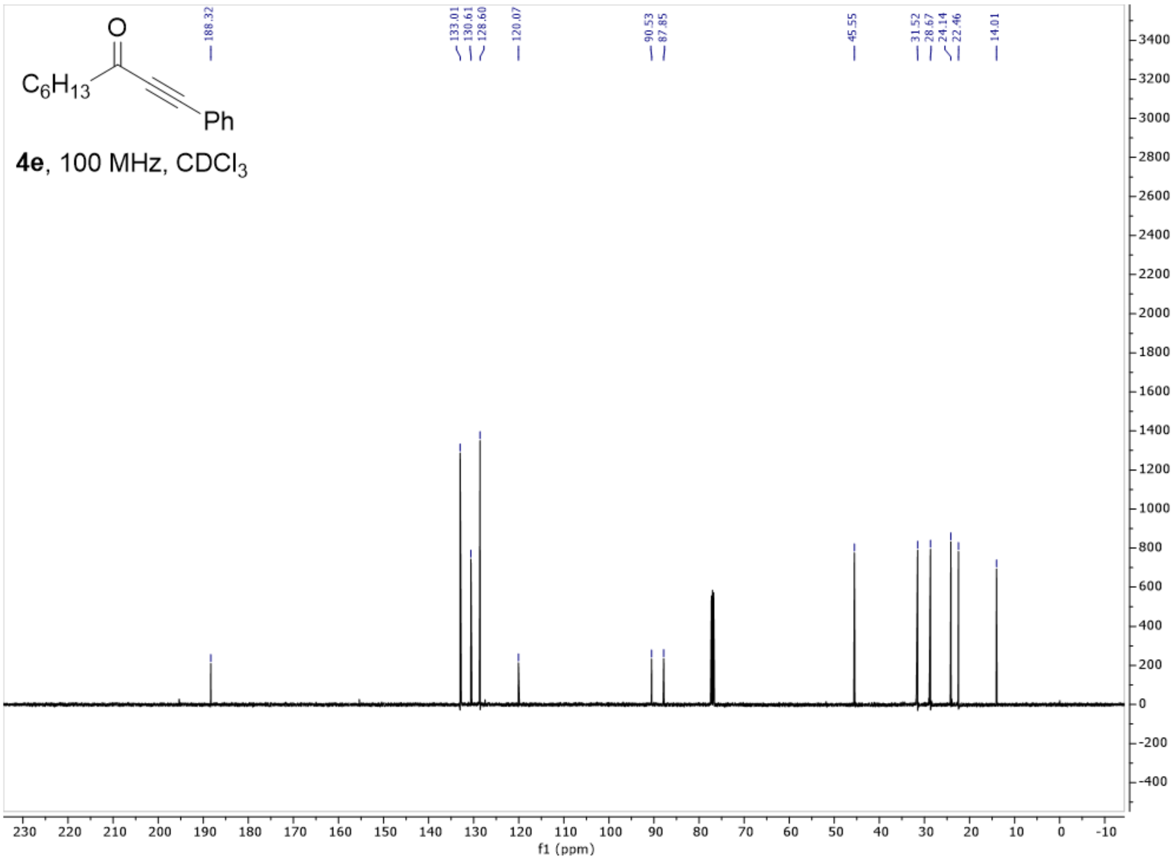

5a: (Hex-1-yn-1-ylsulfonyl)benzene

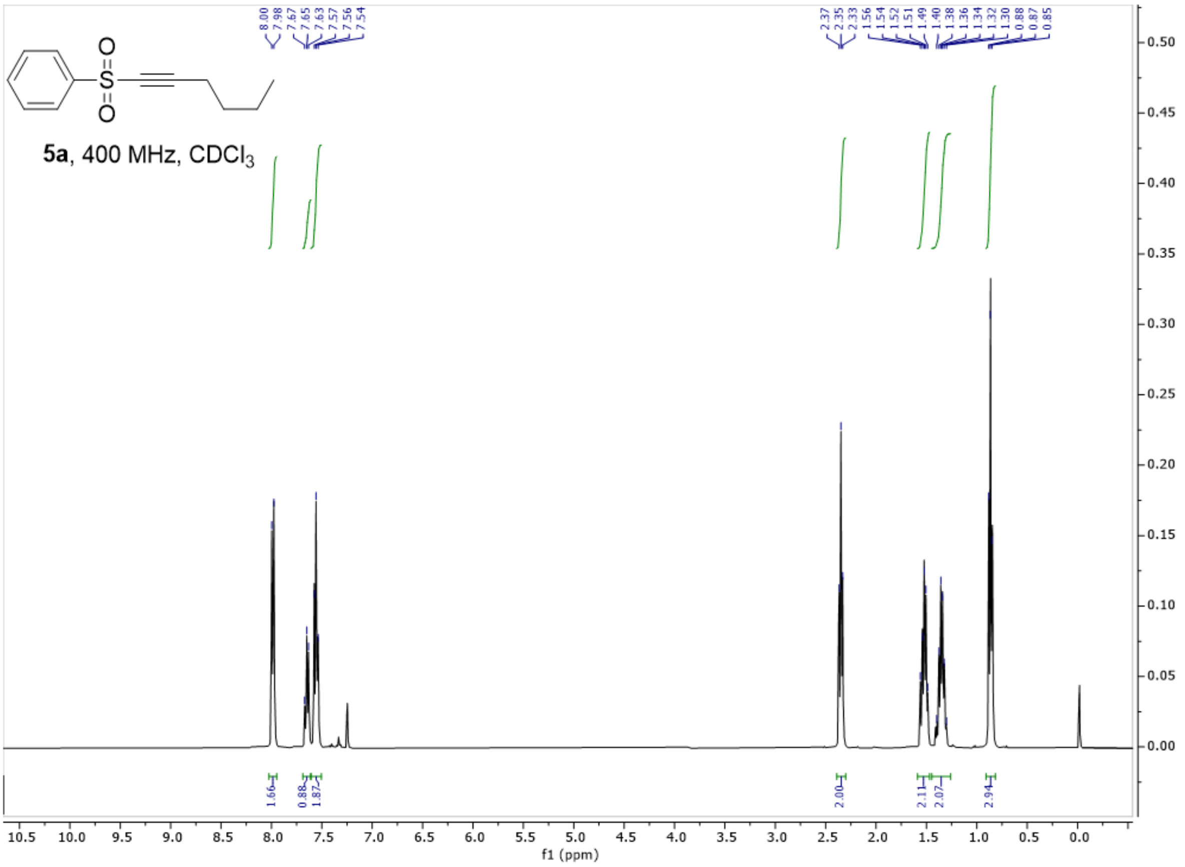

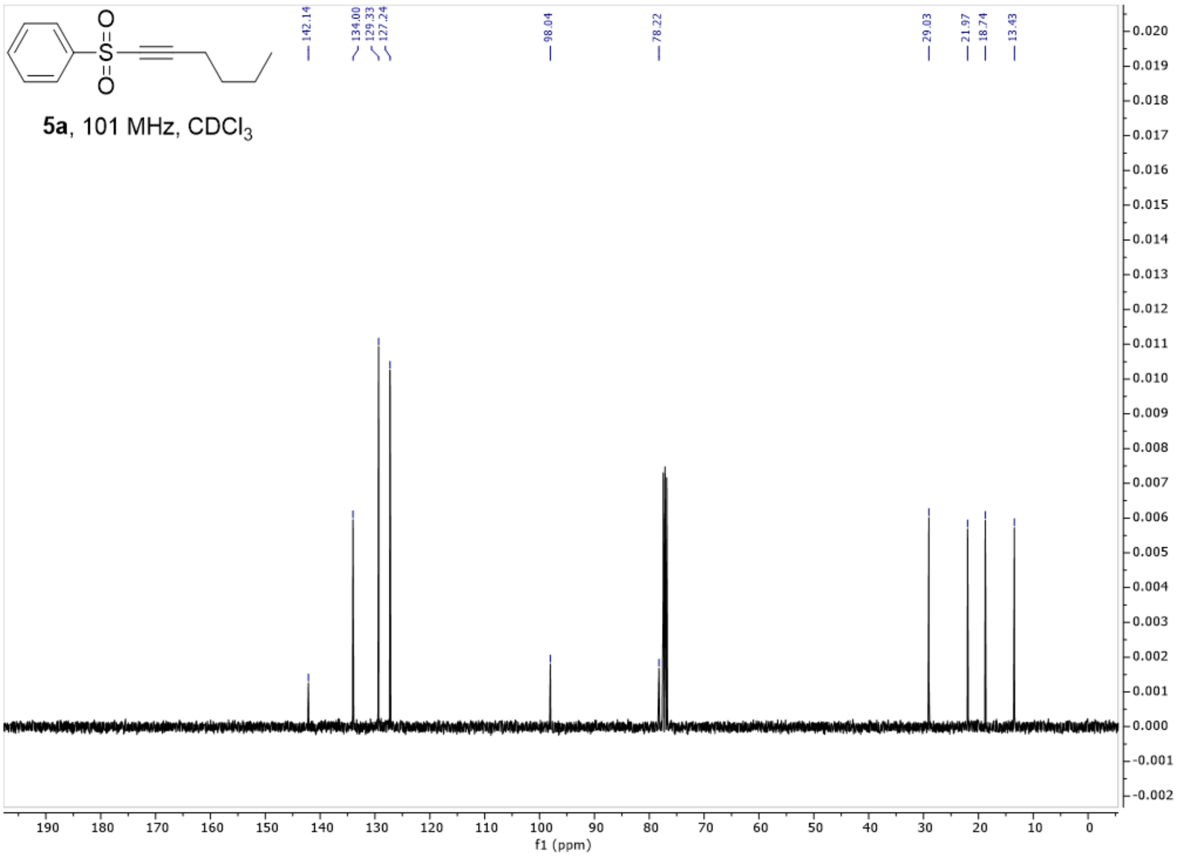

5b: ((Phenylethynyl)sulfonyl)benzene

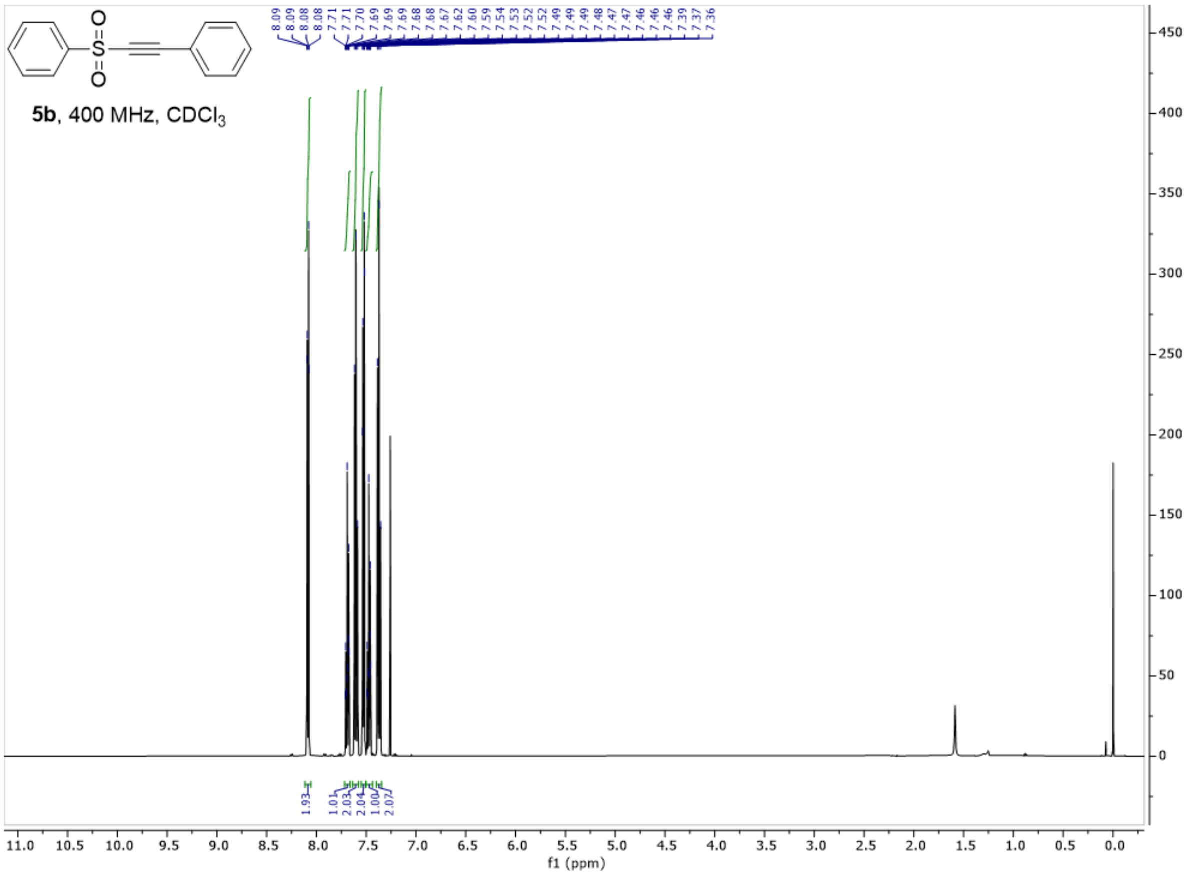

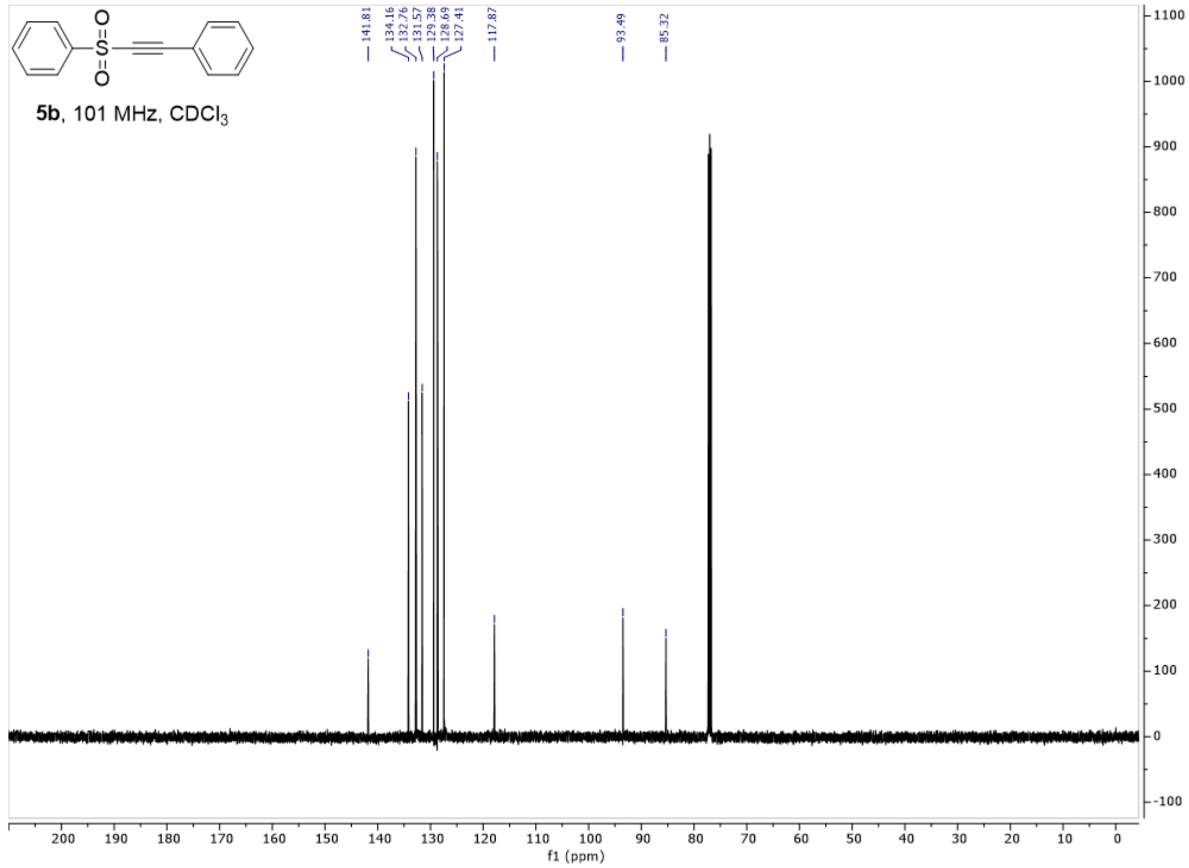

6a: 1-(Hex-1-yn-1-ylsulfonyl)-4-methylbenzene

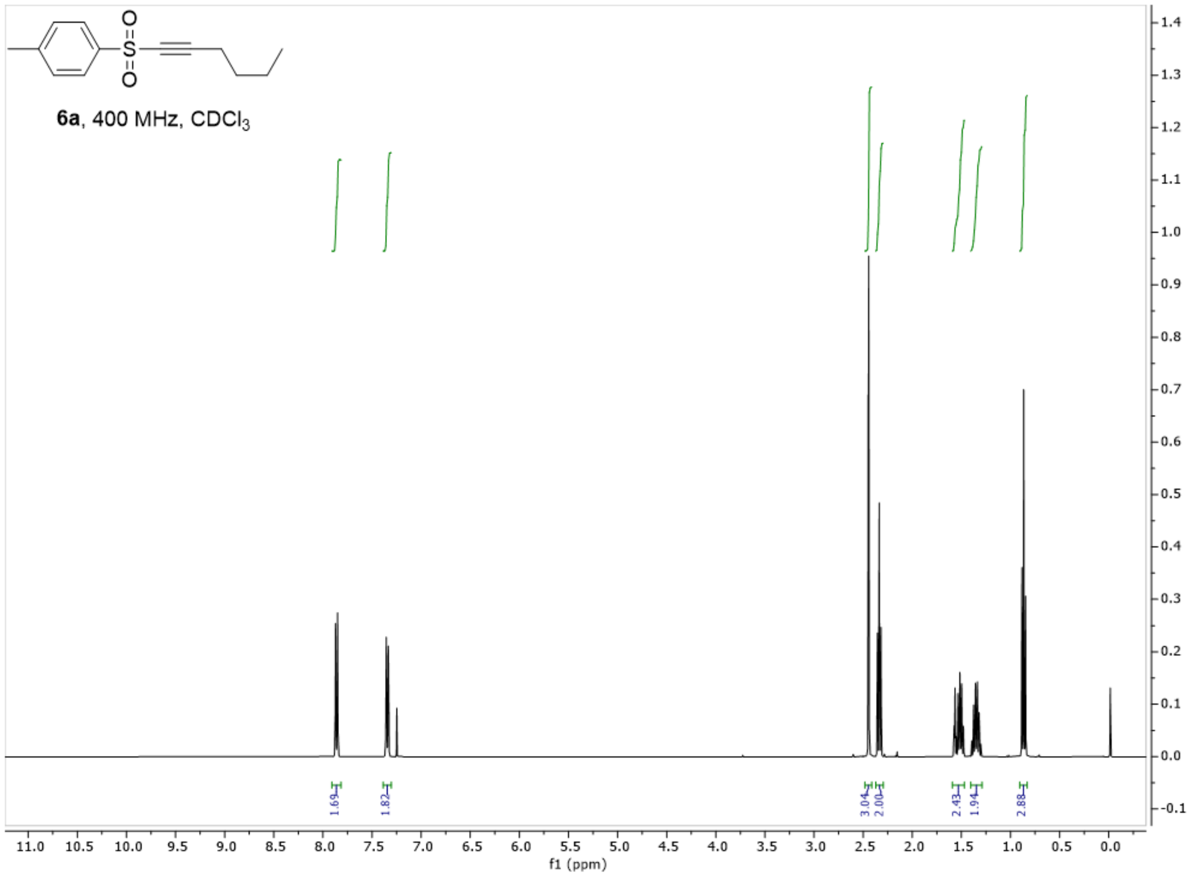

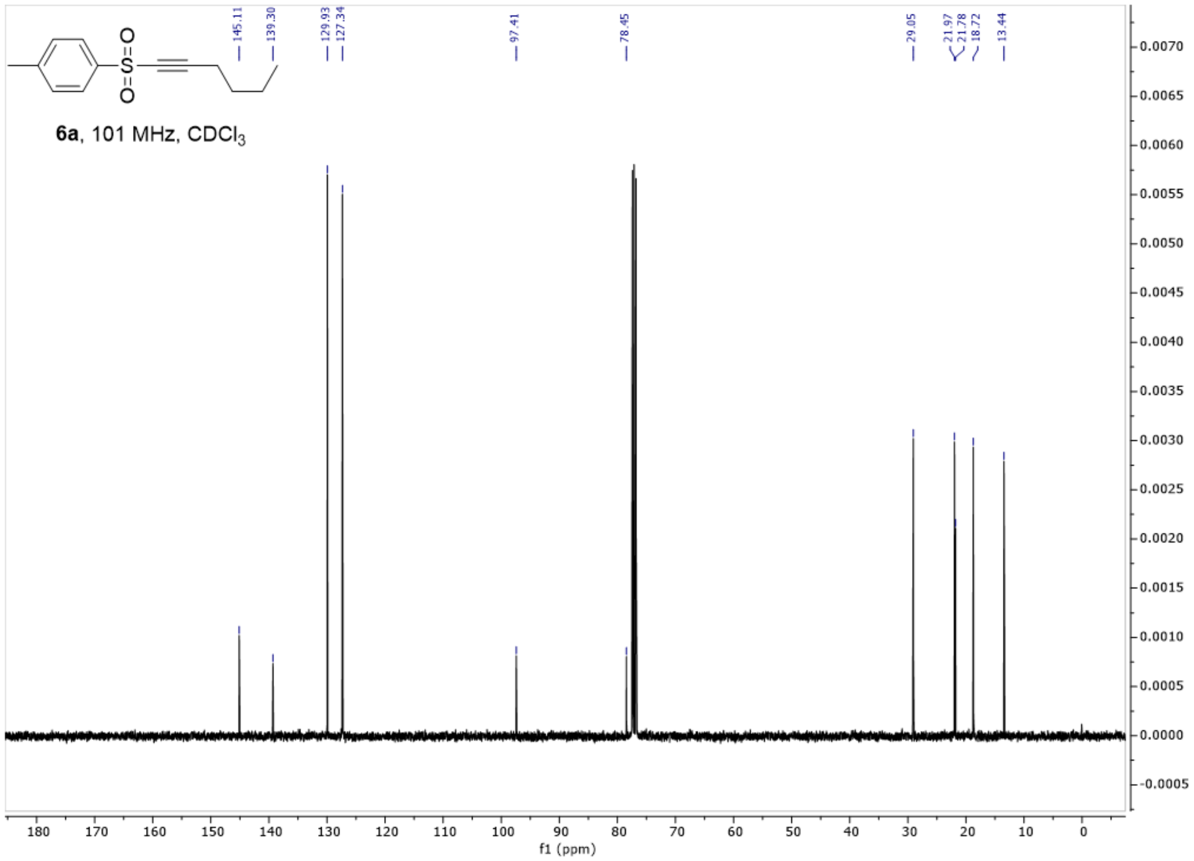

6b: 1-Methyl-4-((phenylethynyl)sulfonyl)benzene

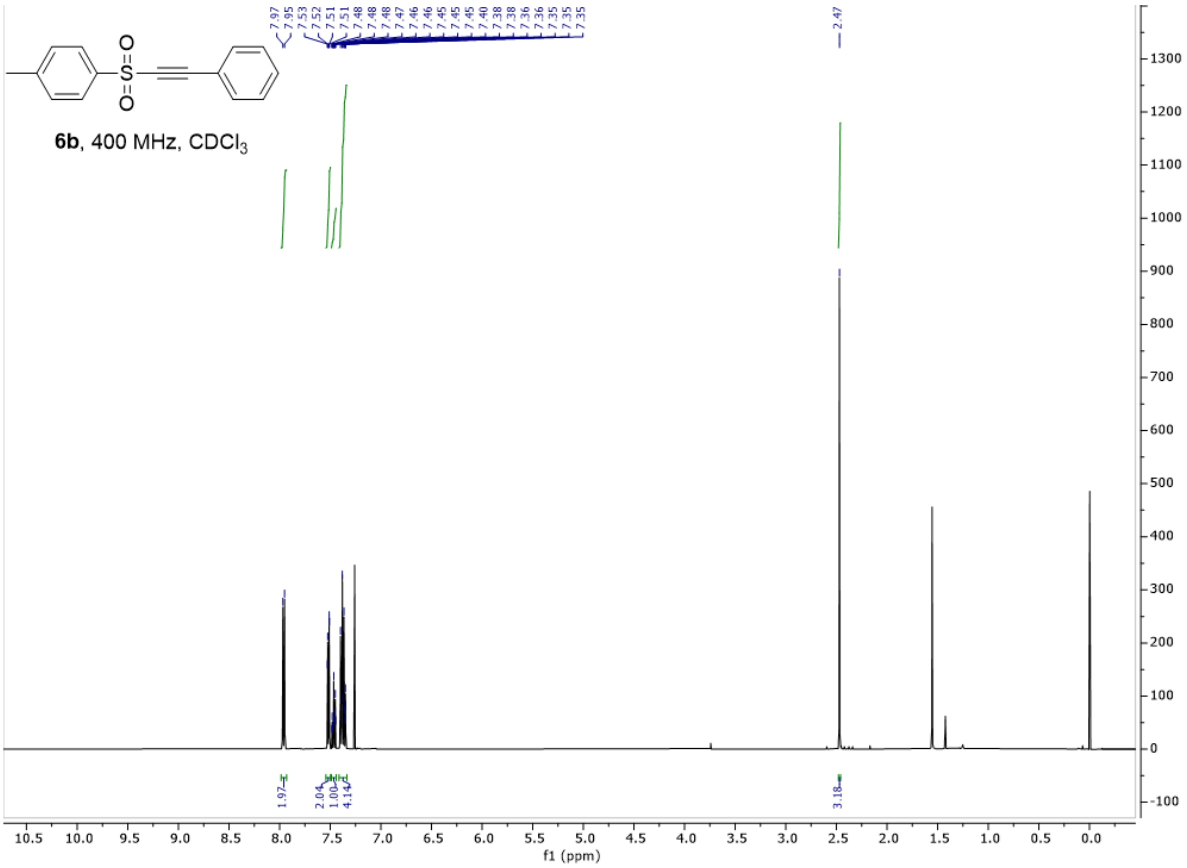

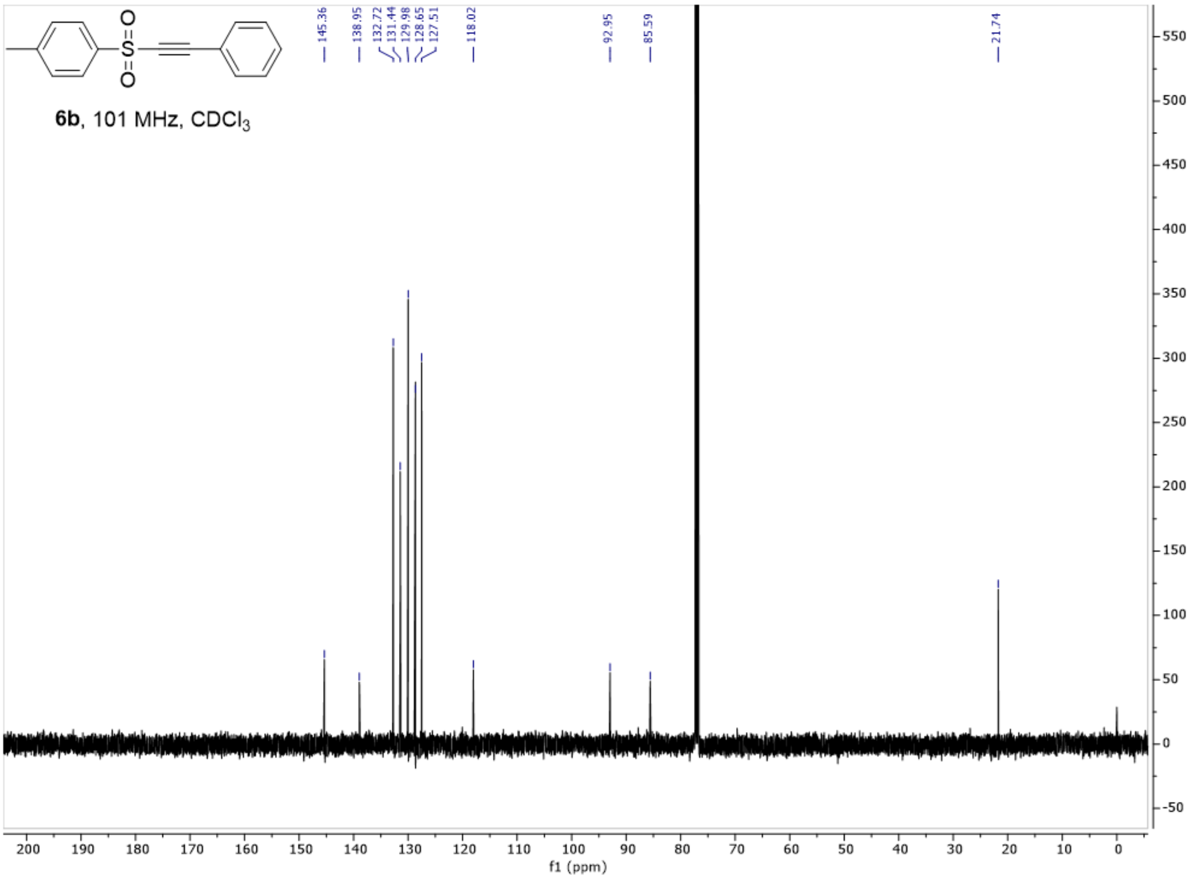

7a: 1-(Methylsulfonyl)hex-1-yne

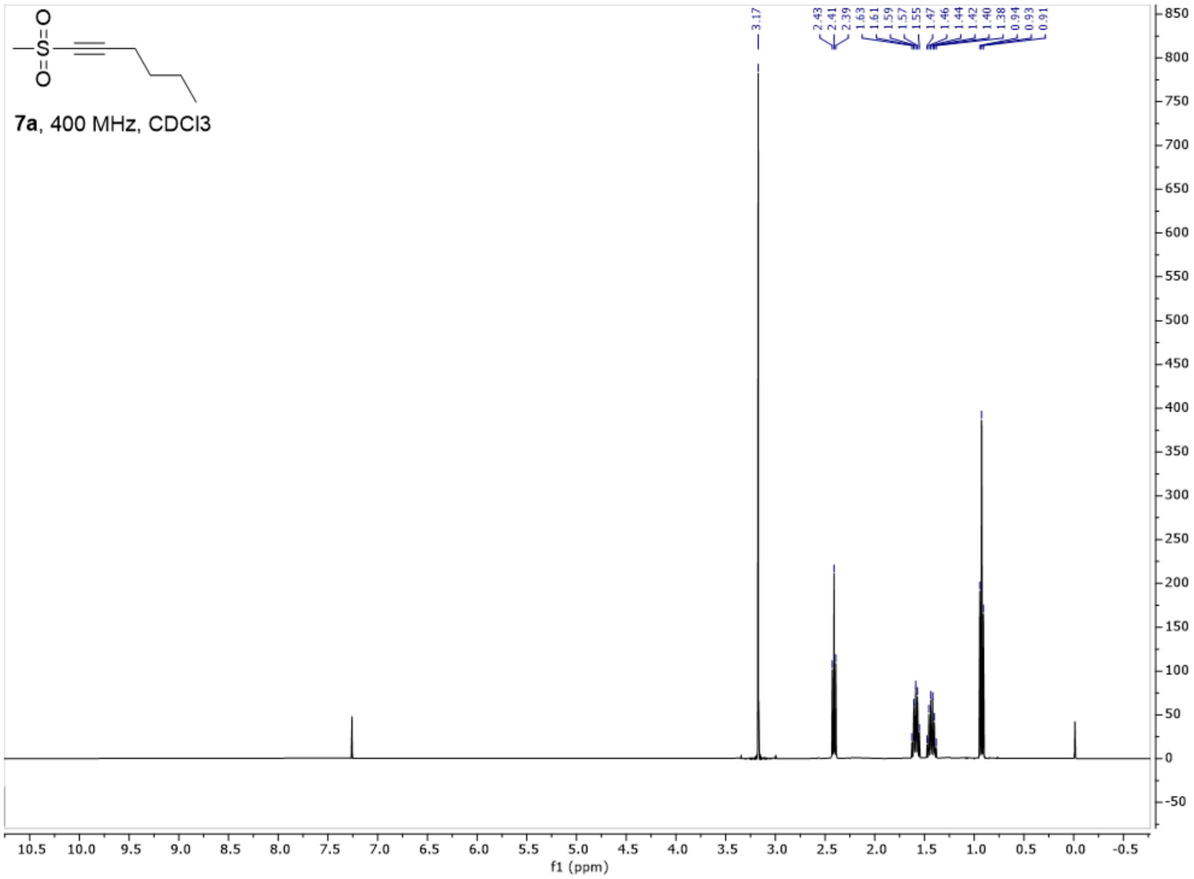

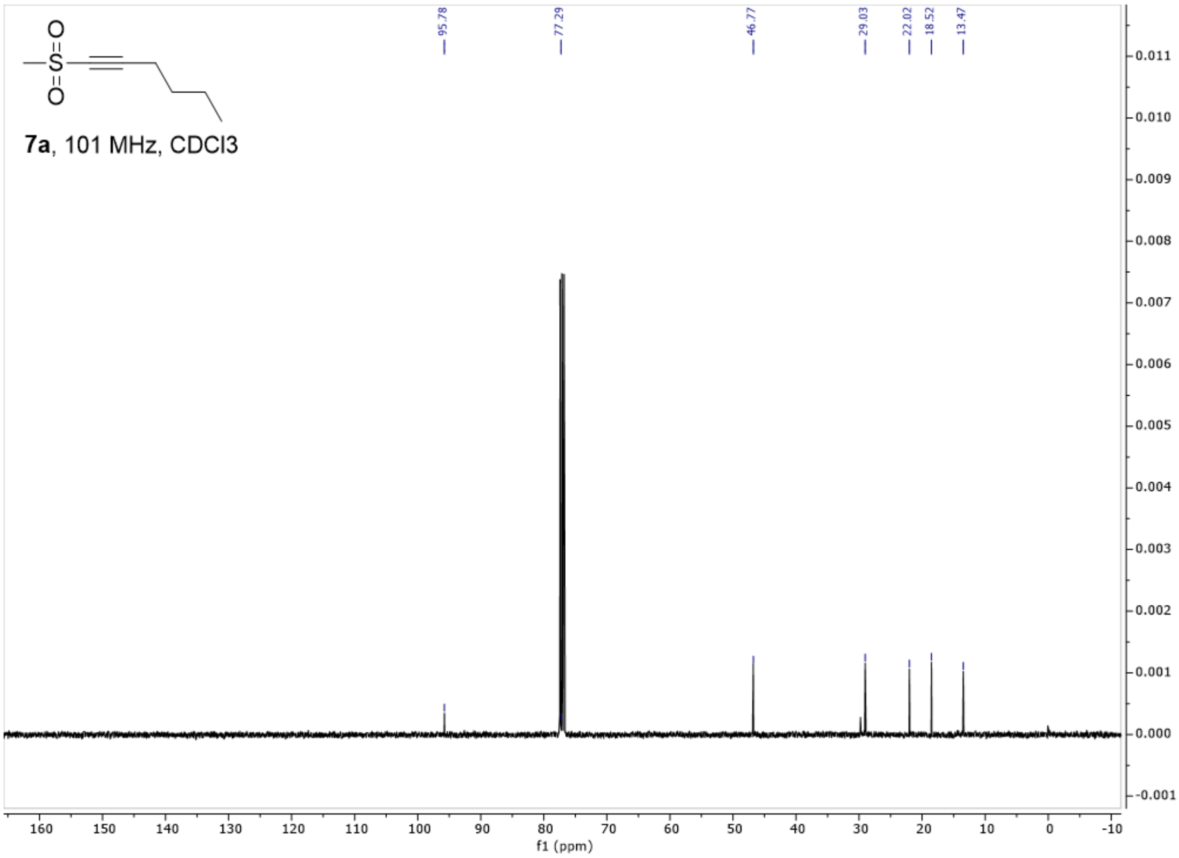

7b: ((Methylsulfonyl)ethynyl)benzene

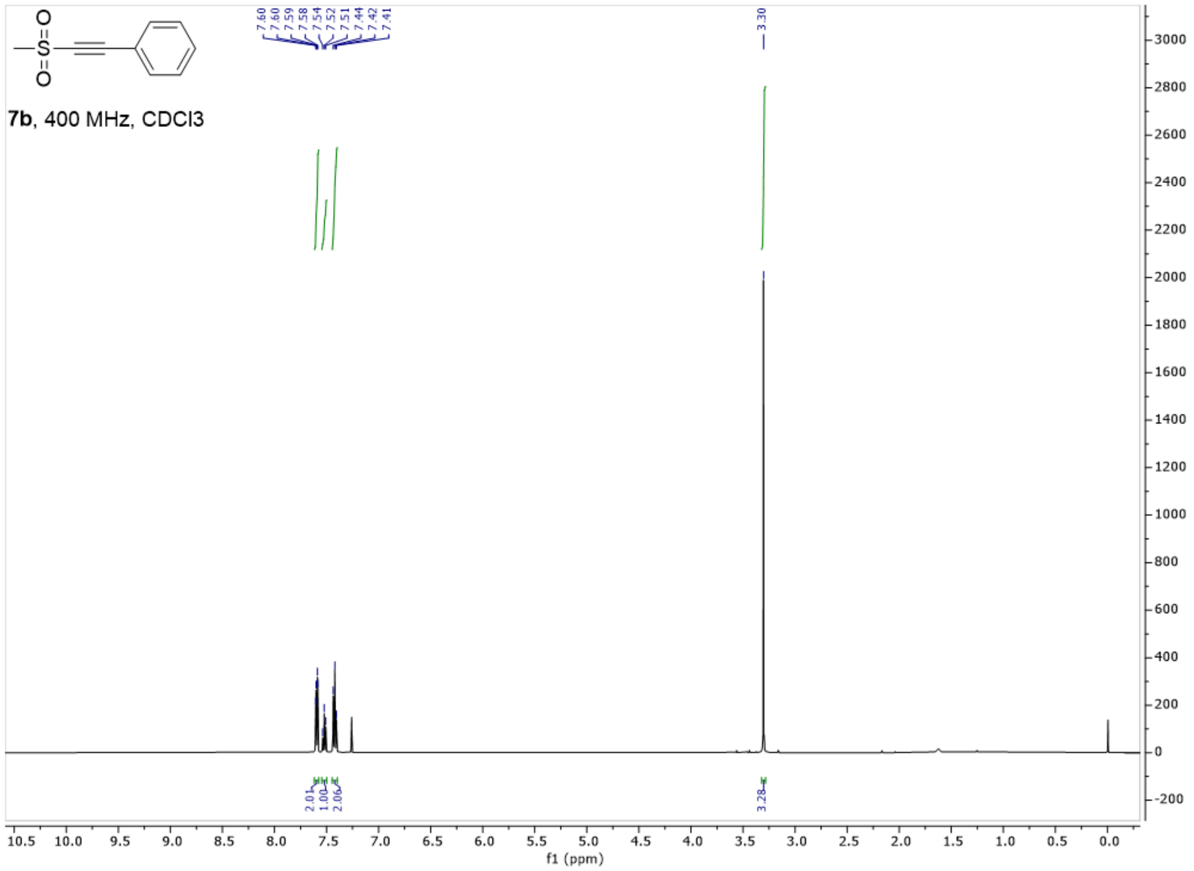

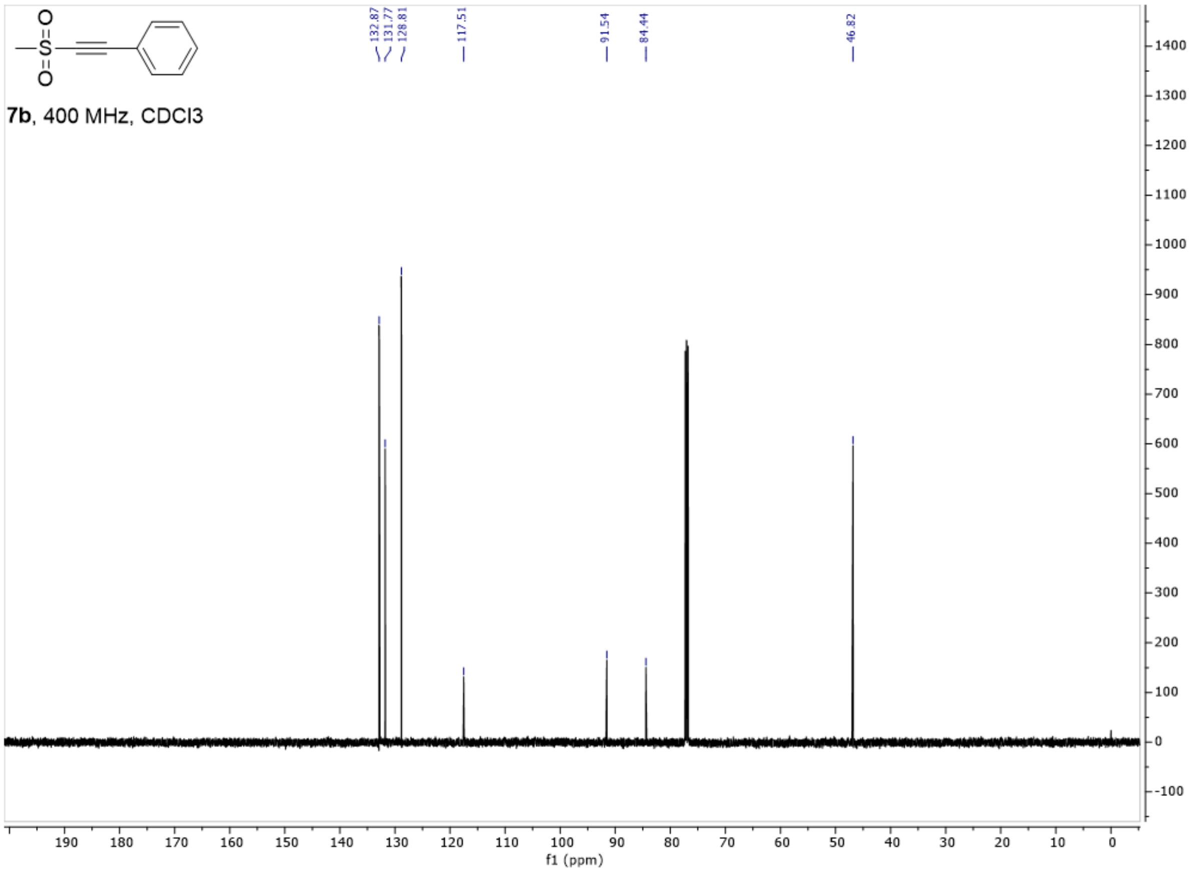

8a: 1-(Ethylsulfonyl)hex-1-yne

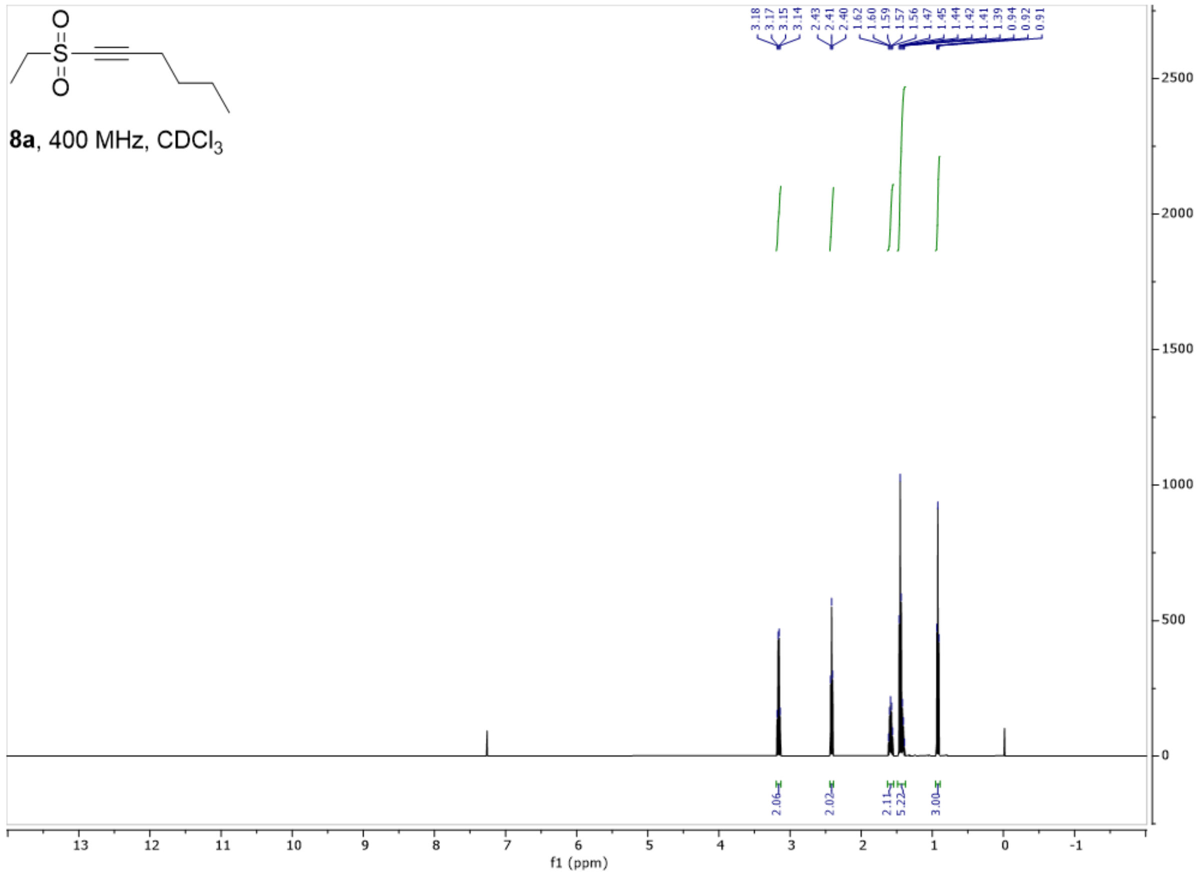

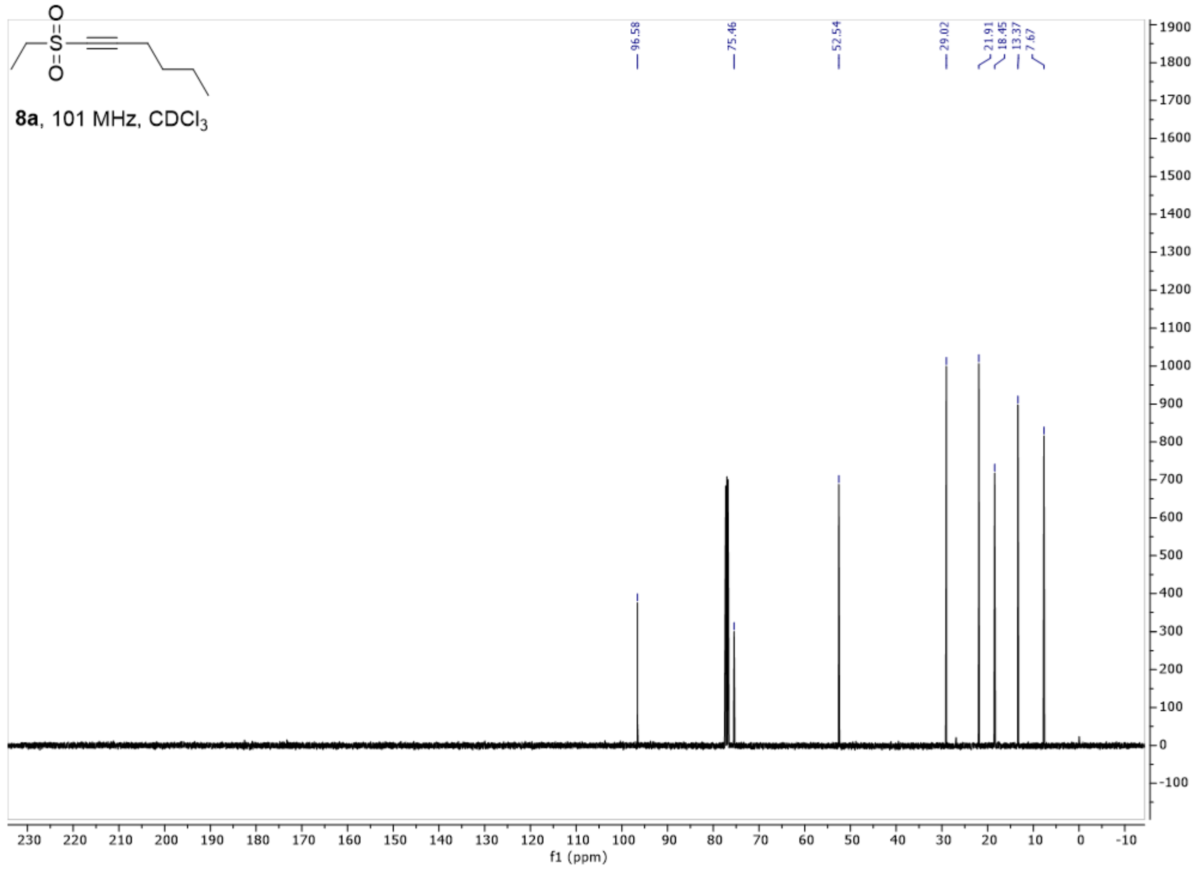

8b: ((Ethylsulfonyl)ethynyl)benzene

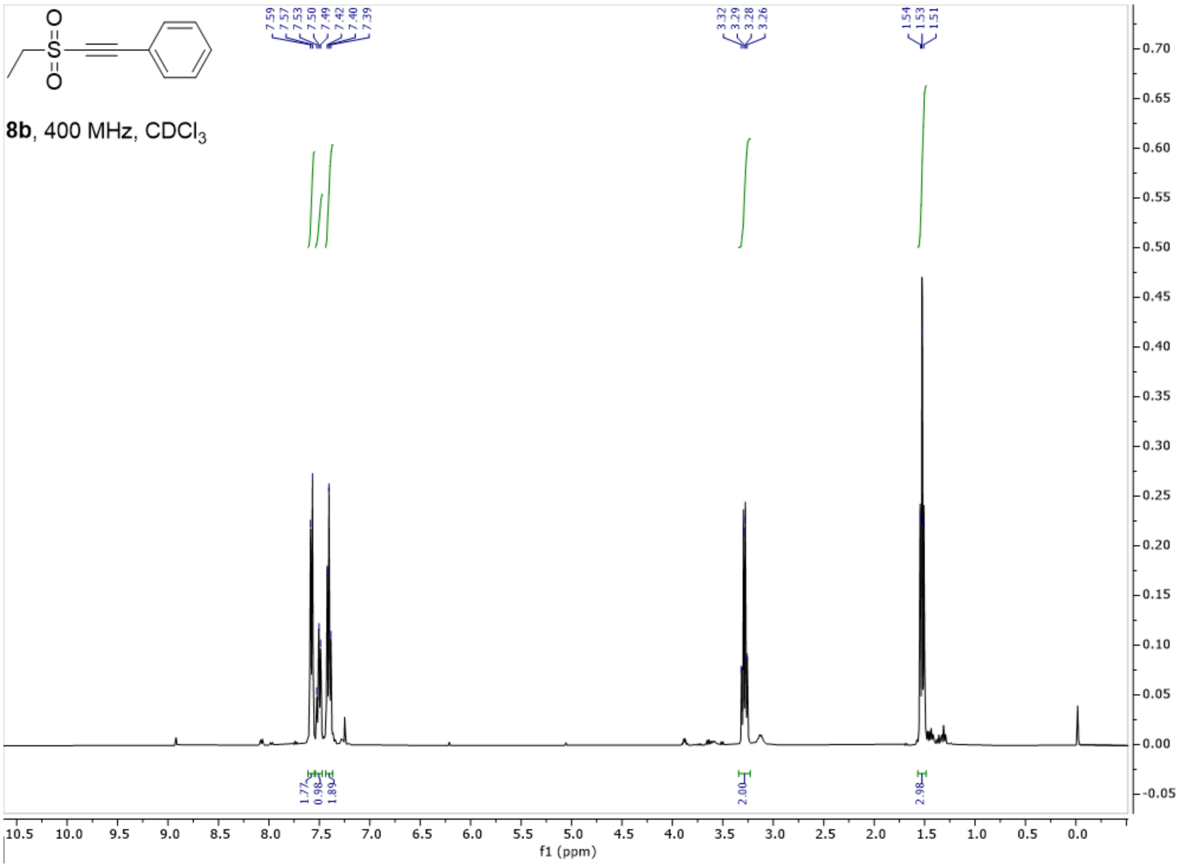

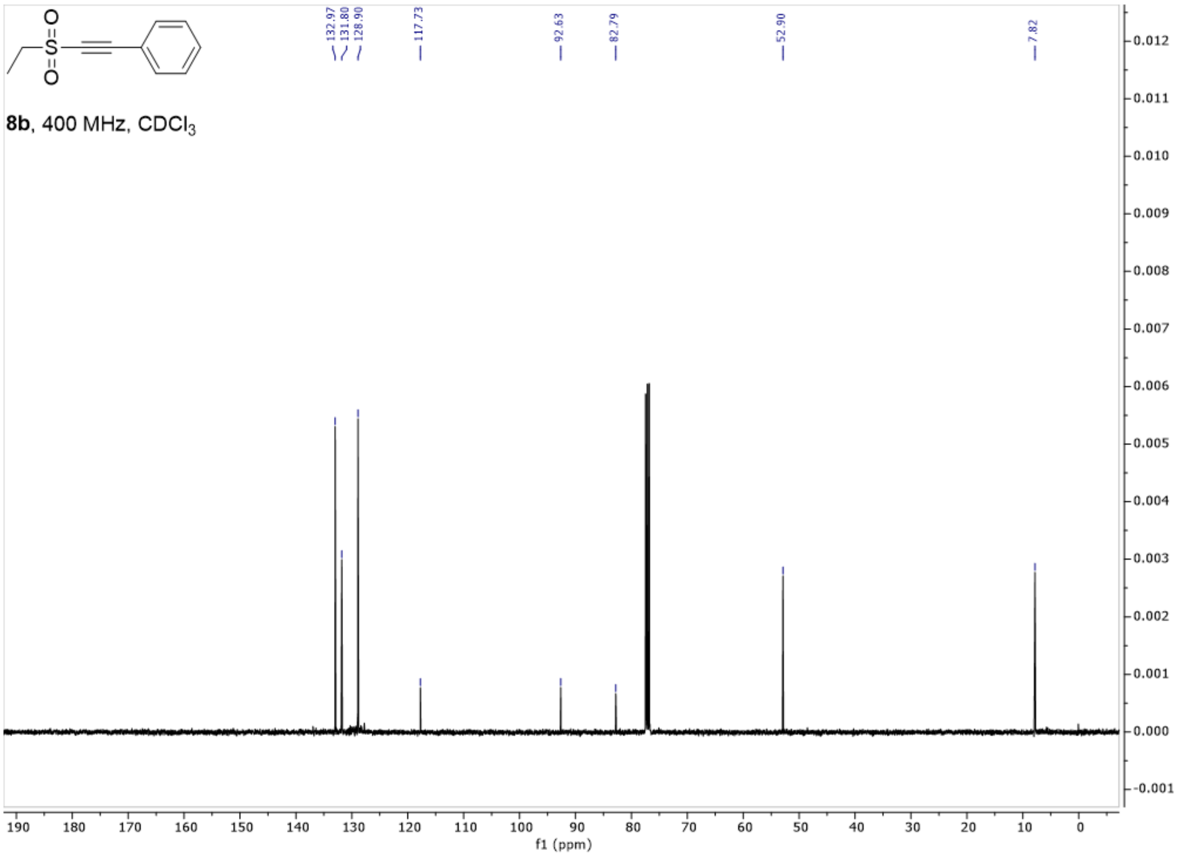

9a: 1-(Isopropylsulfonyl)hex-1-yne

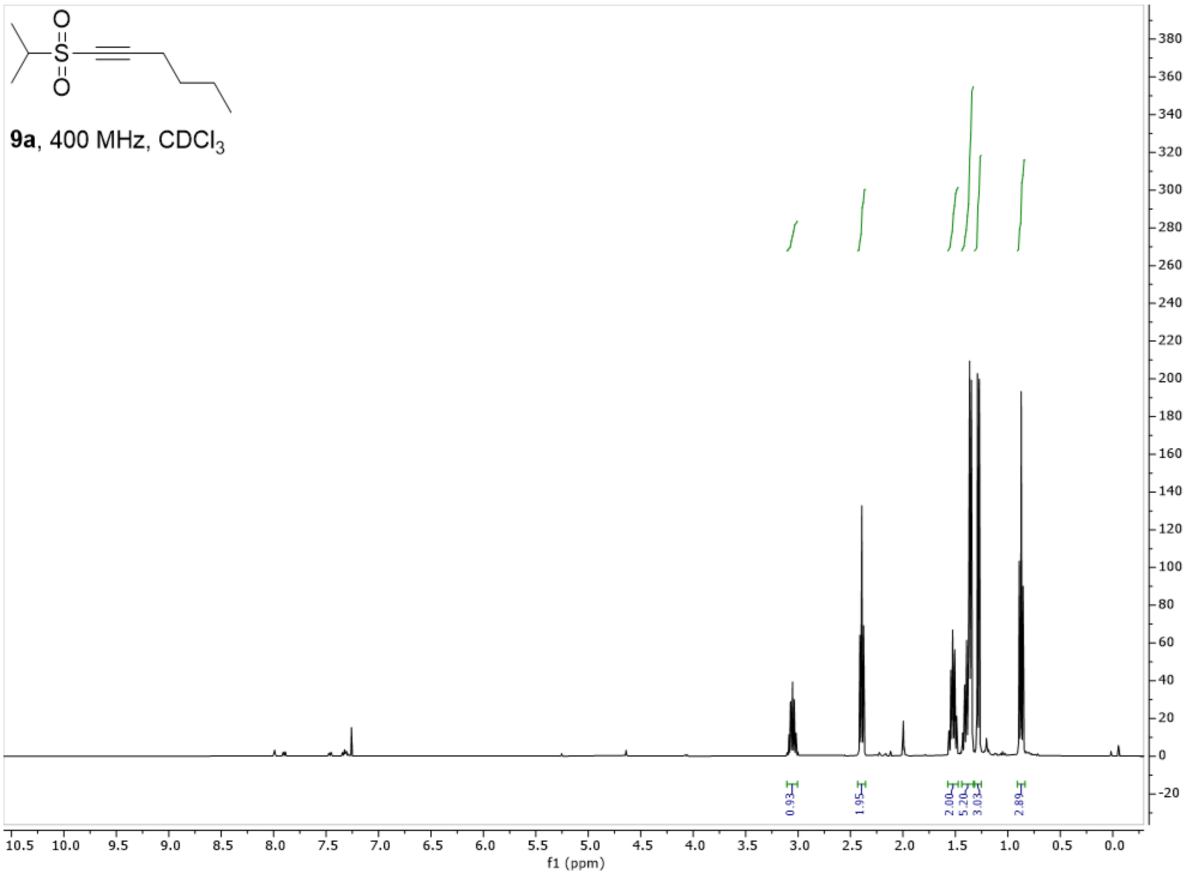

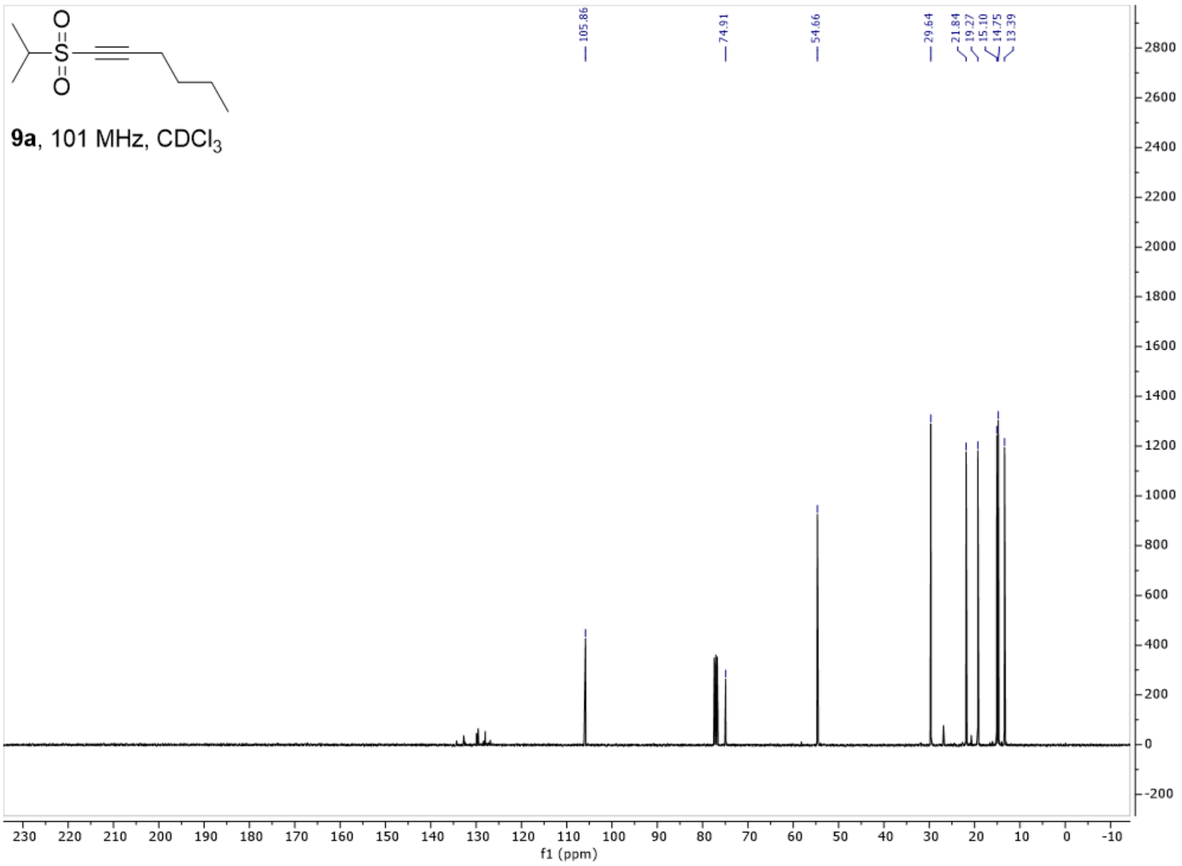

9b: ((Isopropylsulfonyl)ethynyl)benzene

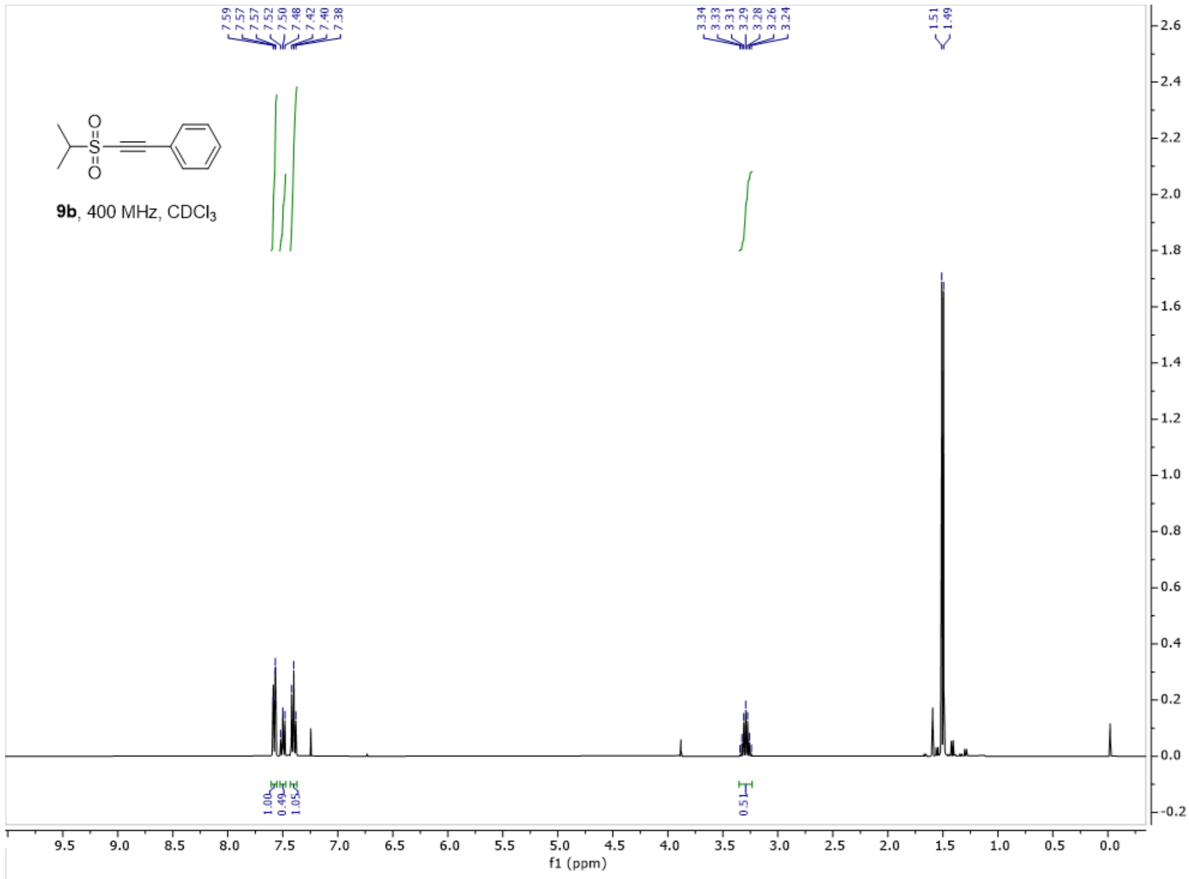

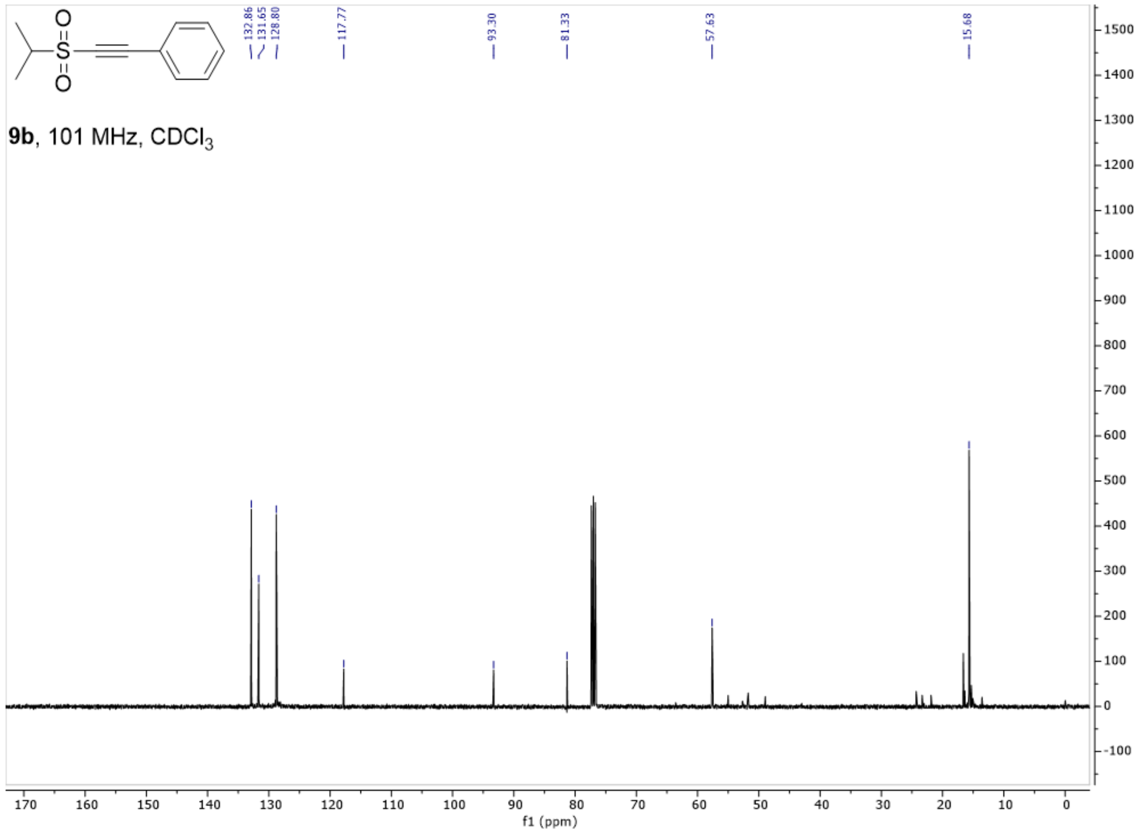

10a: 1-(*tert*-Butylsulfone)hex-1-yne

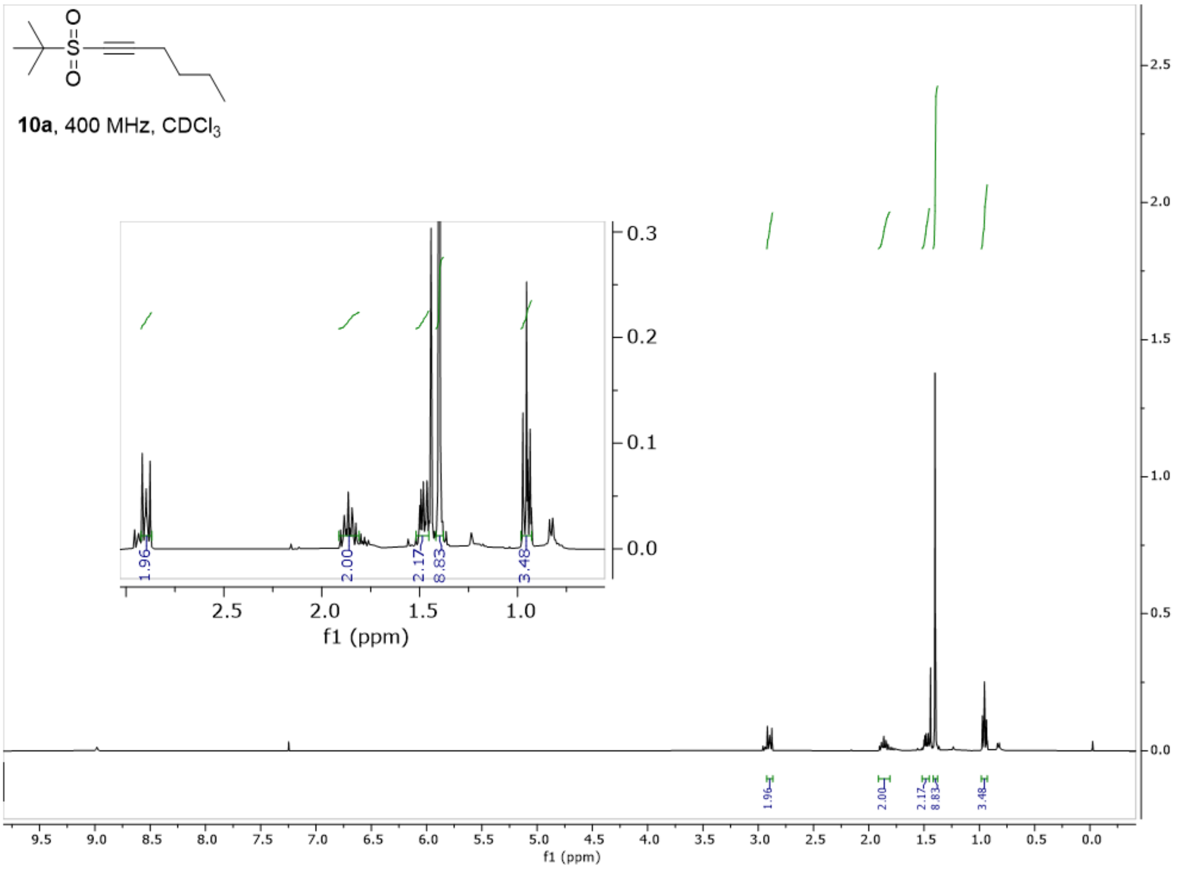

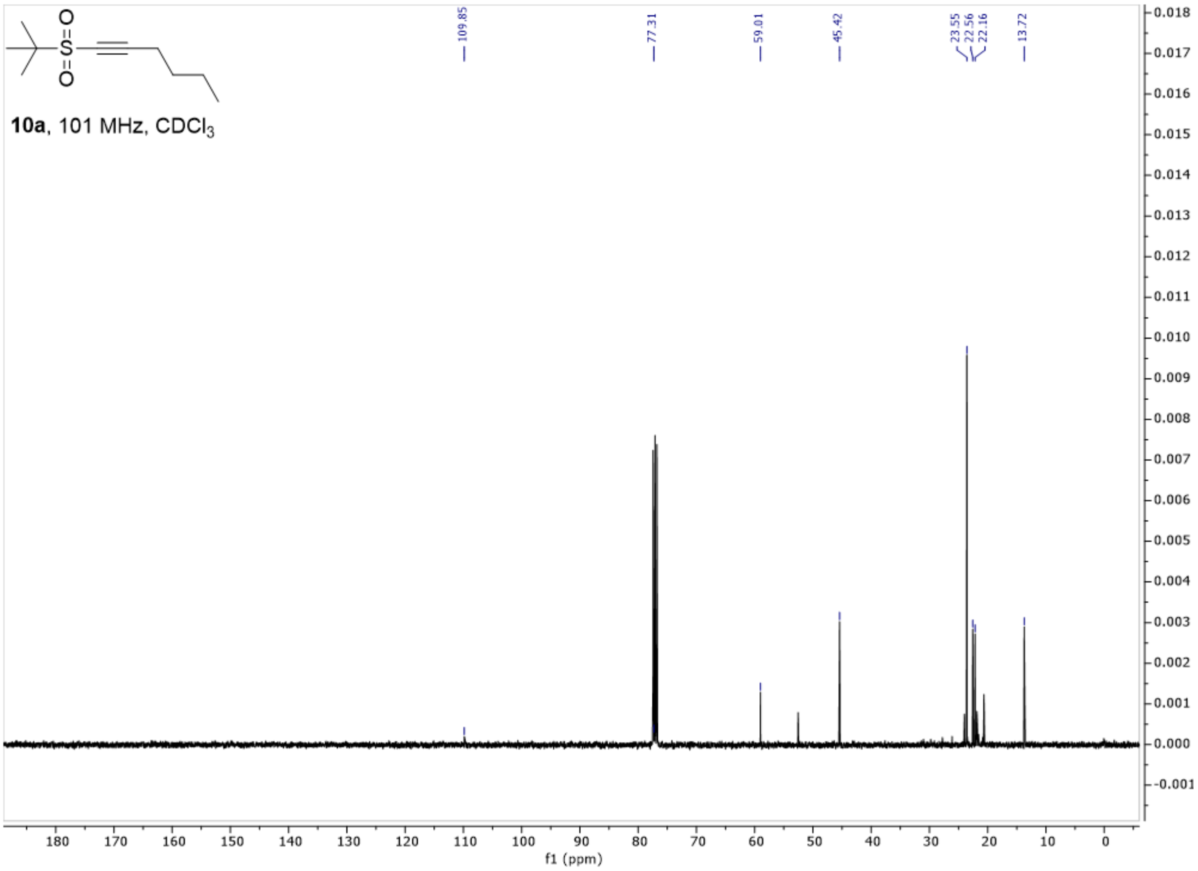

11a: Diethyl hex-1-yn-1-ylphosphonate

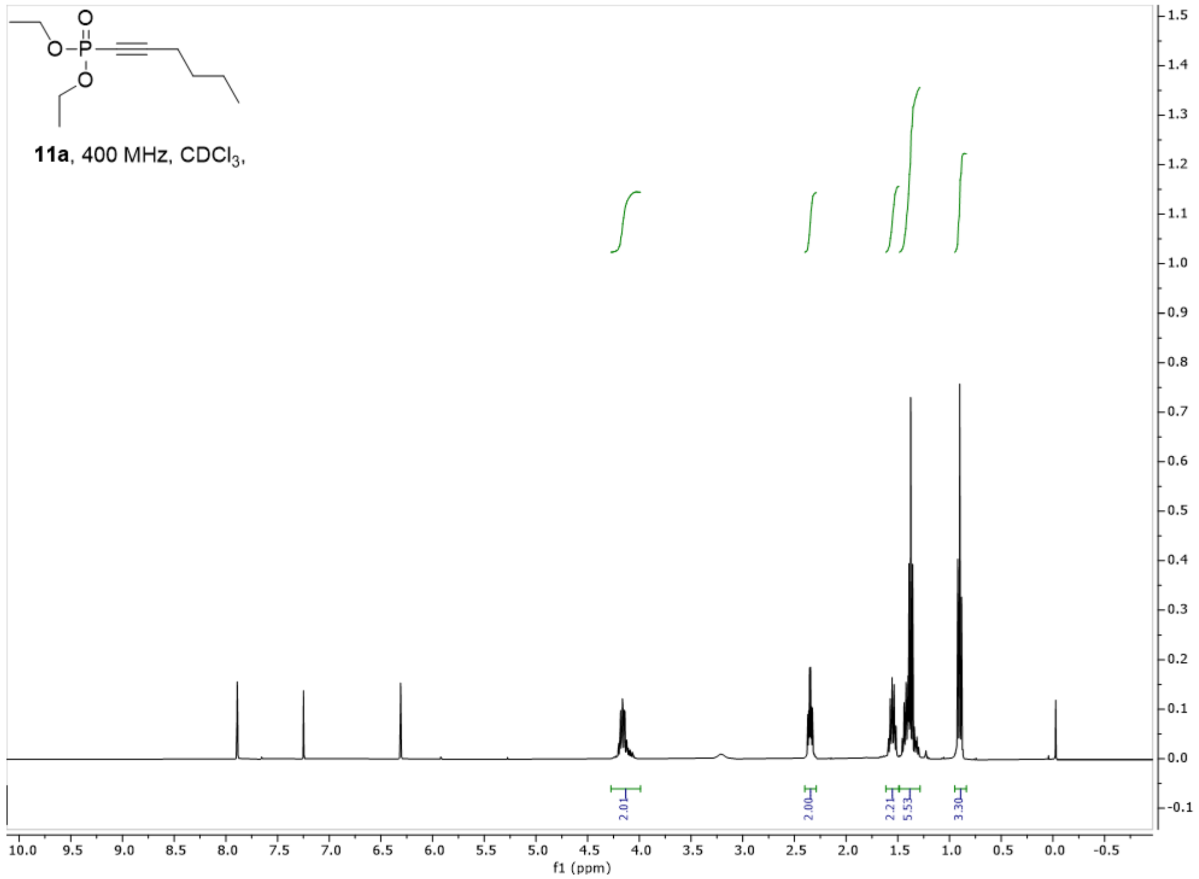

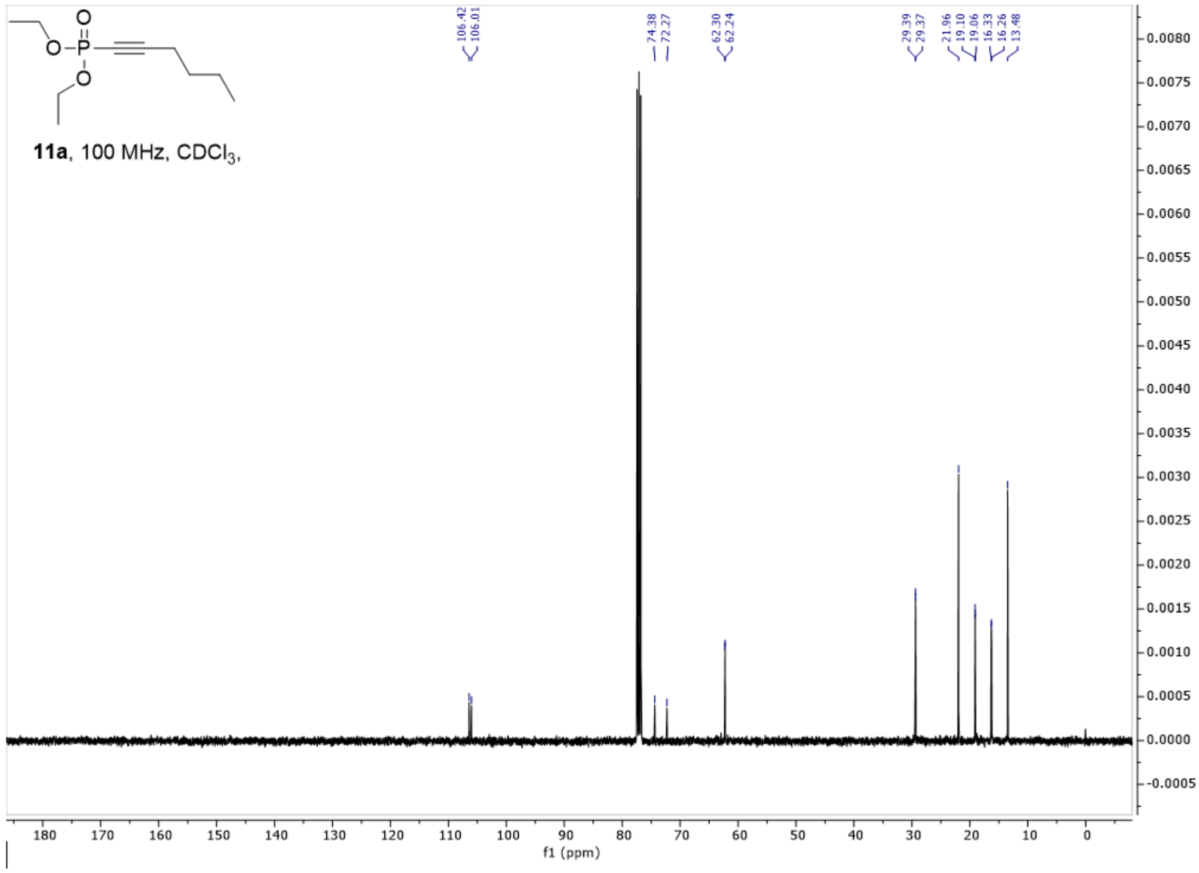

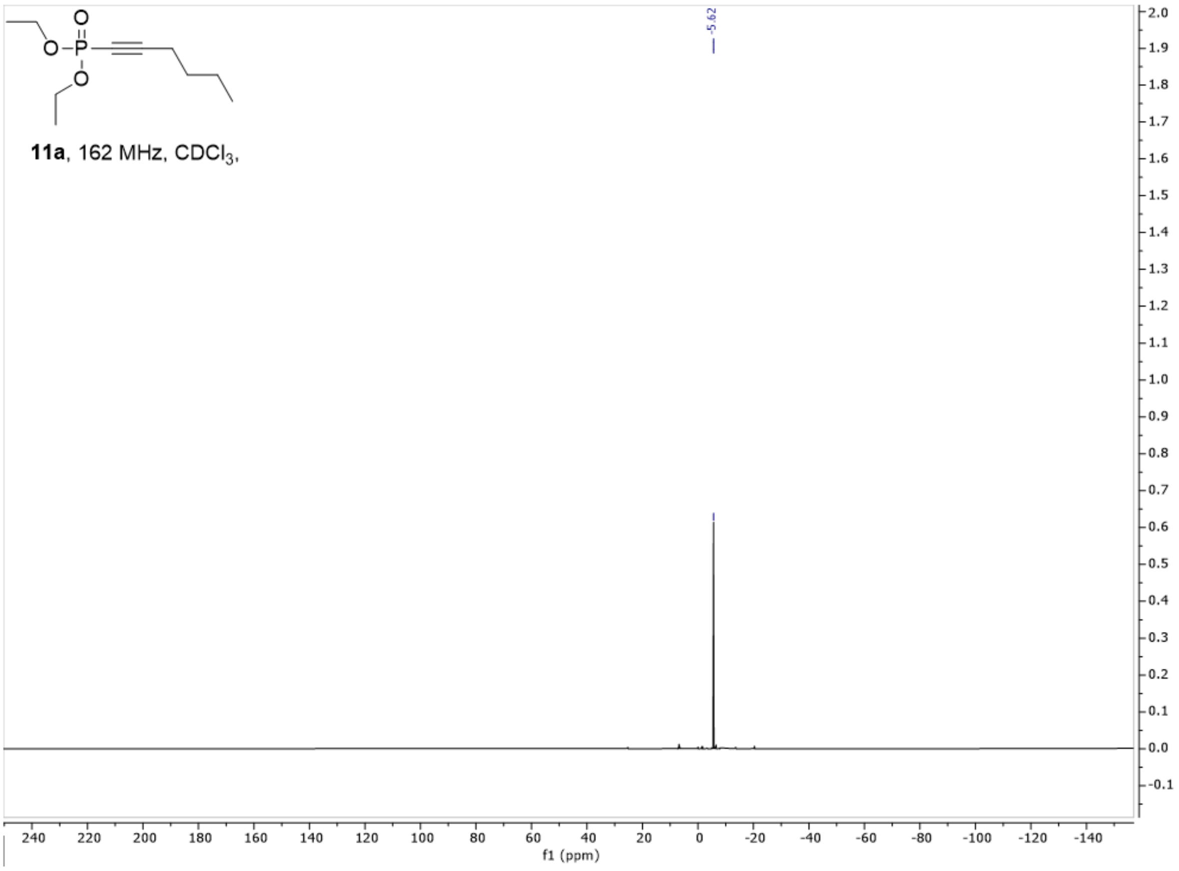
11a, 162 MHz, CDCl3,
-5.62
f1 (ppm)

11b: Diethyl (phenylethynyl)phosphonate

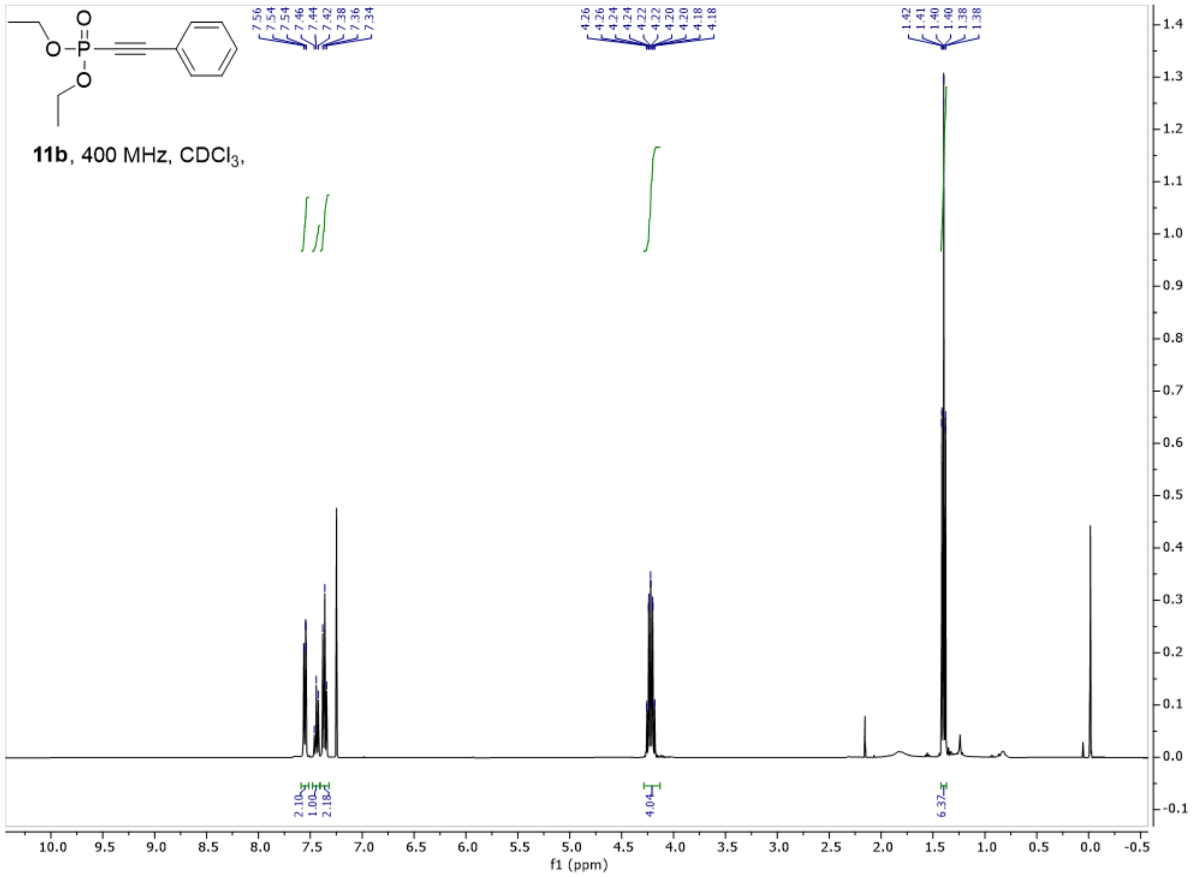

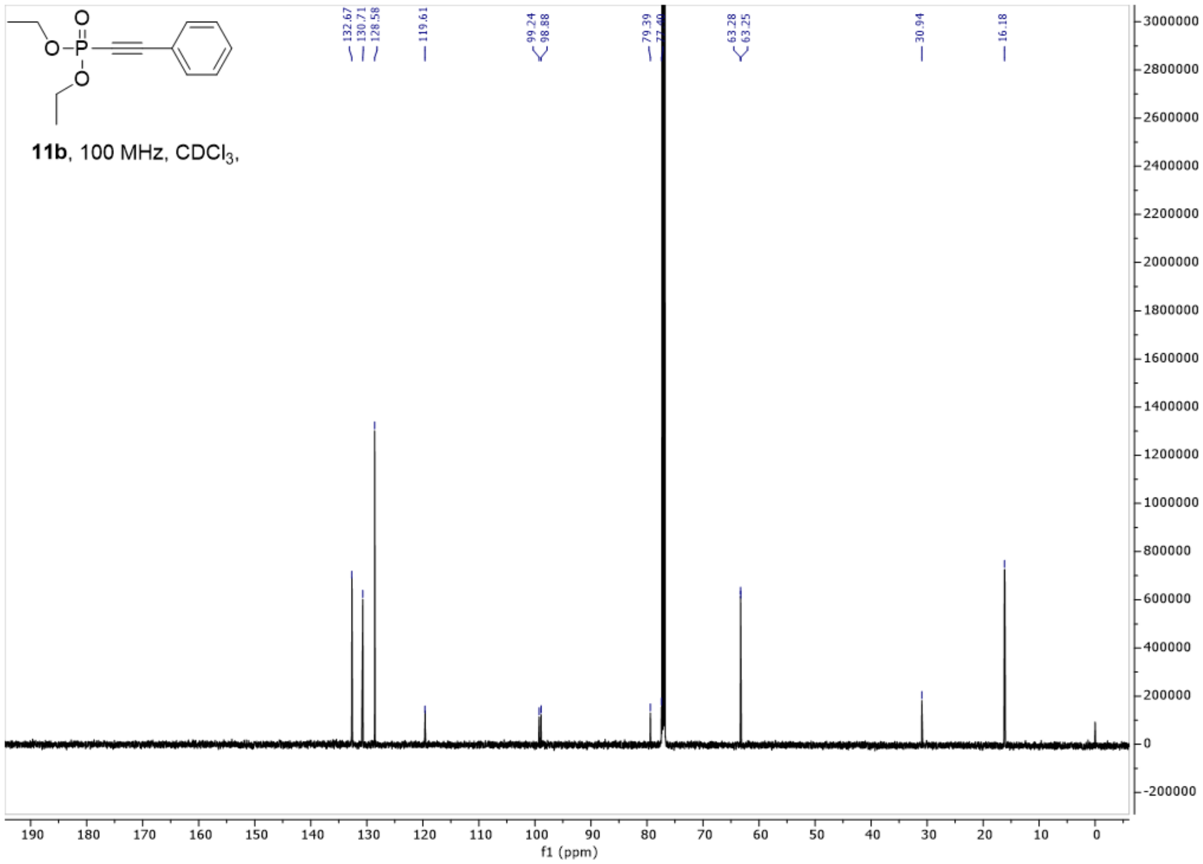

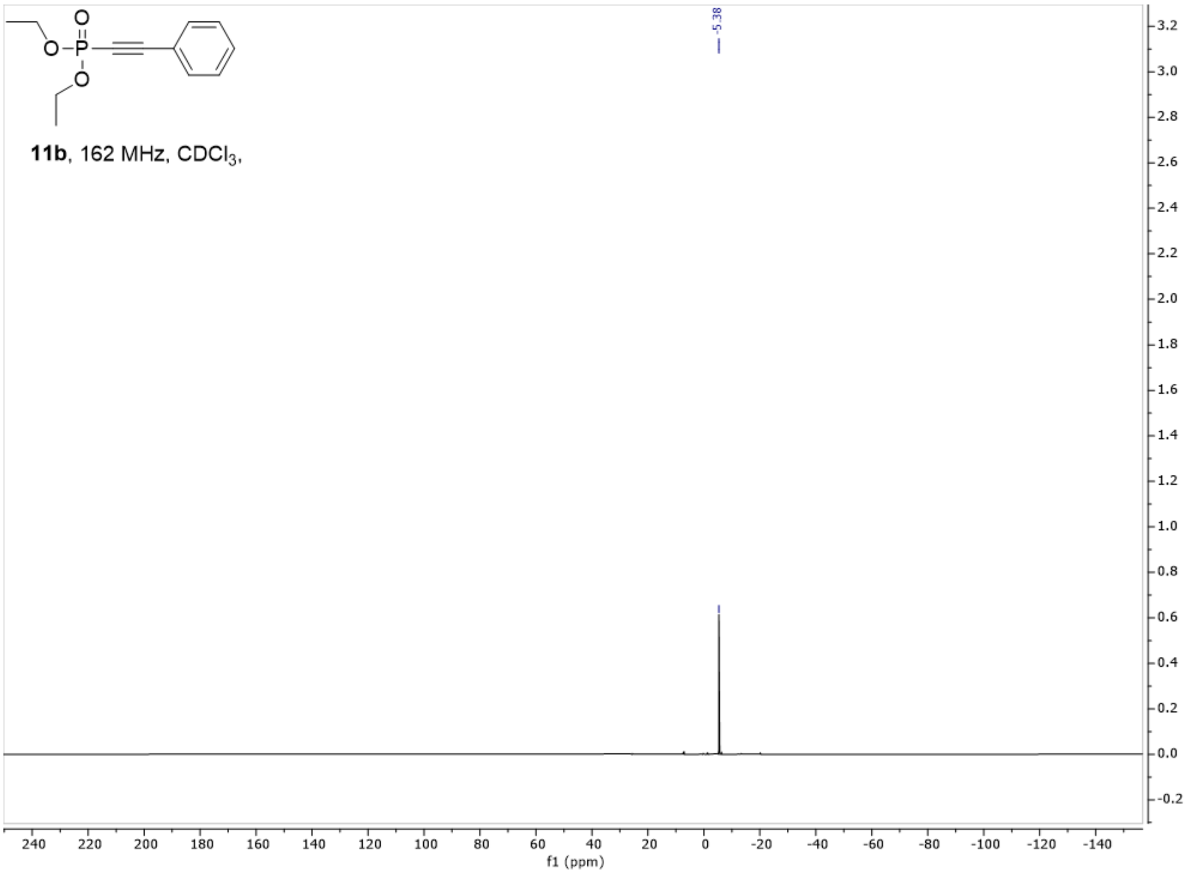
11b, 162 MHz, CDCl3,
-5.38
f1 (ppm)
